# U12 type introns were lost at multiple occasions during evolution

**DOI:** 10.1186/1471-2164-11-106

**Published:** 2010-02-11

**Authors:** Sebastian Bartschat, Tore Samuelsson

**Affiliations:** 1 Department of Medical Biochemistry and Cell Biology, Institute of Biomedicine, Sahlgrenska Academy at University of Gothenburg, Box 440, SE-405 30 Göteborg, Sweden; 2 Bioinformatics Group, Department of Computer Science and Interdisciplinary Center for Bioinformatics, University of Leipzig, Haertelstrasse 16-18, D-04107 Leipzig, Germany

## Abstract

**Background:**

Two categories of introns are known, a common U2 type and a rare U12 type. These two types of introns are removed by distinct spliceosomes. The phylogenetic distribution of spliceosomal RNAs that are characteristic of the U12 spliceosome, i.e. the U11, U12, U4atac and U6atac RNAs, suggest that U12 spliceosomes were lost in many phylogenetic groups. We have now examined the distribution of U2 and U12 introns in many of these groups.

**Results:**

U2 and U12 introns were predicted by making use of available EST and genomic sequences. The results show that in species or branches where U12 spliceosomal components are missing, also U12 type of introns are lacking. Examples are the choanoflagellate *Monosiga brevicollis*, *Entamoeba histolytica*, green algae, diatoms, and the fungal lineage Basidiomycota. Furthermore, whereas U12 splicing does not occur in *Caenorhabditis elegans*, U12 introns as well as U12 snRNAs are present in *Trichinella spiralis*, which is deeply branching in the nematode tree. A comparison of homologous genes in *T. spiralis *and *C. elegans *revealed different mechanisms whereby U12 introns were lost.

**Conclusions:**

The phylogenetic distribution of U12 introns and spliceosomal RNAs give further support to an early origin of U12 dependent splicing. In addition, this distribution identifies a large number of instances during eukaryotic evolution where such splicing was lost.

## Background

In eukaryotes mature RNA is formed by the removal of introns from a primary transcript. Splicing is catalyzed by a multicomponent complex, the spliceosome [1]. Two intron classes have been identified, a common U2-type and a rare U12-type [2-4]. Splicing of U2-type introns is catalyzed by the U2-dependent (major) spliceosome, which includes the U1, U2, U4, U5 and U6 spliceosomal RNAs as well as multiple protein factors. The U12-dependent (minor) spliceosome, responsible for the excision of the U12-type introns, is structurally similar to the U2-type spliceosome. It contains protein subunits and the U5 RNA as well as the U11, U12, U4atac, and U6atac spliceosomal RNAs that are functionally and structurally related to the U1, U2, U4 and U6 RNAs of the major spliceosome.

U2 introns have characteristic properties at the 5' splice site (AG/GURAGU), 3' splice site (YAG/G) and branch site (CURACU, where the A is the branch point adenosine). There is also a pyrimidine rich region between the branch and 3' splice sites. Much of the specificity in the splicing reaction is accomplished by pairing with snRNAs. Thus, the 5' splice site pairs with U1 RNA and the branch site pairs with U2 RNA.

The U12 introns have consensus sequences that are different from U2 introns. The 5' splice site (/RTATCCTTT) as well branch site (UCCUUAACU, where the underlined A is the branch point adenosine) are more conserved than their counterparts in U2 introns, whereas the 3' splice site is more variable. In addition, U12 introns lack a pyrimidine rich region. Whereas the vast majority of U2 introns have the dinucleotides GT and AG at their 5' and 3' ends, respectively, some U12 introns have the dinucleotides AT and AC in these positions [5]. During U12 splicing, the 5' splice site and branch site pair with the U11 and U12 snRNA, respectively.

U2-type introns are ubiquitous in eukaryotes while U12-type introns are lacking in some species, such as *Saccharomyces cerevisiae *[6] and in the nematode *Caenorhabditis elegans *[5]. U12 introns were first reported only in vertebrates, insects, cnidarians and plants [5]. However, they were later discovered in *Rhizopus oryzae*, *Phytophthora *and *Acantamoeba castellanii*, demonstrating an early evolutionary origin for the U12 spliceosome [7].

We have recently presented an inventory of spliceosomal RNAs based on computational prediction from genomic sequences [8]. We found additional support of U12 splicing in *Acanthamoeba castellanii *as we identified the U12-type spliceosomal U11 and U6atac RNAs, in addition to the previously identified U12 RNA [7]. Furthermore, RNAs specific to the U12 spliceosome were identified in a number of phylogenetic groups where previously such RNAs were not observed, including the nematode *Trichinella spiralis*, the slime mold *Physarum polycephalum *and the fungal lineages Zygomycota and Chytridiomycota. The detailed map of the distribution of the U12-type RNA genes supports an early origin of the minor spliceosome and points to a number of occasions during evolution where it was lost.

We have now addressed the question of whether the distribution of U12-type RNAs is correlated with the distribution of U12 introns. If there is such a correlation we also wanted to examine mechanisms of U12 intron evolution. Possible events regarding the fate of U12 introns as discussed by Burge et al [5] include U12 intron loss as well as conversion of introns from the U12 to the U2 category by mutational changes. The database of orthologous U12 introns, U12DB[9], lists examples of changes in the latter category.

A number of methods have been developed for the prediction of U12 type introns [5,10,11]. Most of them make use of weight matrices based on known exon-intron boundary regions and branch sites [5,11]. In addition, AT-AC-type introns with classic consensus features may be identified with a simple pattern-based approach [7].

For this study we have used the methods of Burge et al [5] as well as that of Sheth et al [11] to predict U2 and U12 introns in a number of different species that represent a broad phylogenetic range. The results show that the distribution of U12 introns is consistent with the distribution of U12 spliceosomal components.

## Methods

### Sequence data

EST sequences were retrieved using NCBI Entrez. Genomic sequences were downloaded from WUSTL http://genome.wustl.edu, *T. spiralis *version 1.0, and *Physarum polycephalum *version 3.1), Wormbase (*Caenorhabditis elegans*, http://www.wormbase.org/), JGI (http://www.jgi.doe.gov/, *Monosiga brevicollis *version 1.0, *Phycomyces blakesleeanus *version 1.0, *Chlamydomonas reinhardtii *v 3.0, *Phytophthora sojae *version 1.0, *Thalassiosira pseudonana *version 3.0, *Phaeodactylum tricornutum *assembly 1), from Broad Institute (*Rhizopus oryzae *assembly 3, *Phytophthora infestans *version 1.0), from TIGR (*Entamoeba histolytica *2004 version) and from TraceDB (*Phakopsora pachyrhizi *and *Acanthamoeba castellanii*).

### Identification of U12 introns

Introns were identifying from BLAST searches [12] where EST sequences were used to query a database of genomic sequences. For instance, in the case of *T. spiralis*, a total of 25,268 EST sequences were retrieved using NCBI Entrez and used to query a database of *T. spiralis *genome sequences. The genome sequences contained 15544 contigs with a total of 115,634,429 nt. Only hits with sequence identity at least 98% and HSP length at least 35 nt were considered for further analysis.

Whenever an EST matched to more than one genomic contig sequence we selected for further analysis the contig with the most extensive match to the EST sequence. As BLAST is often not able to unambiguously identify the exact location of the splice site, we considered all possible sites and the most probable one was identified by screening with position weight matrices (PWMs) as described below.

PWMs for 5', 3' and branch sites of the GT-AG U12, GT-AG U2, AT-AC U12 and GC-AG U2 type of introns from five different species were obtained from the Splicerack database http://katahdin.cshl.edu:9331/SpliceRack/index.cgi?database=spliceNew. For the 5' splice sites the PWM covers 13 positions where the first 3 positions are the 3' end of the exon, and for the 3' splice site the window has 17 positions when the last 3 positions are in the exon part. The branch site PWM has a length of 12 and corresponds to a location falling into the range of (-40,-5) upstream of the 3' splice site of the intron. PWMs were available for *C. elegans*, *D. melanogaster*, *A. thaliana*, *H. sapiens *and *M. musculus *but only the first three of these were used for the scoring of *T. spiralis *sequences as these PWMs are more appropriate to nematodes as well as to the other species examined here [11].

We used 5' and 3' matrices from *C. elegans*, *D. melanogaster *and *A. thaliana *to score all possible intron locations as inferred by BLAST. Each possible position of the intron therefore generated three different sets of 5' and 3' scores. We finally selected the intron position where both 5' and 3' scores were the greatest or, in cases where this was not applicable, where the sum of both scores was the greatest.

For identification of U12 introns using the method of Sheth et al [11], pseudocounts of 0.001 were added to the PWMs available from the Splicerack database http://katahdin.cshl.edu:9331/SpliceRack/ and log-odd matrices were then obtained. In the case of the branch site scoring we used only one PWM, as the PWMs of the five different species were identical. For identifying the most likely branch site every segment of length 12 within the range (-40,-5) relative to the 3' splice site was scored with U12 GT-AG and AT-AC matrices.

For prediction of U12 introns using the Burge et al method [5] we used for scoring the frequency matrices of U2 GT-AG, U12 GT-AG, and U12 AT-AC dependent introns from SpliceRack. As there is no matrix for branch sites of GT-AG U2 introns we created such a matrix in the following way. For every U2 intron of *T. spiralis *classified with the method from Sheth et al. we used the branch site which achieved the best score with a GT-AG U12 PWM. All the 14480 branch sites obtained in this way were used to construct a frequency matrix.

The scores for each splice or branch site were then computed as described in Burge et al. [5]. The 5' splice site probability is defined as  where the probability of base j in position i is , U is either U12 or U2 and X describes the sequence to be scored. To score the branch site, the values of  and  are calculated for each 13 nt segment in the range (-40,-5) relative to the 3' splice site and the maximum values of both calculations were retained. The complete 5' splice site scores and branch site scores are  and , respectively. These two values were calculated for every intron found. The corresponding sample mean and standard deviation were determined and these scores were normalized to z scores *S*_5'*ss *_and *S*_*bps *_by subtracting the sample mean and dividing by the standard deviation.

After scoring all introns we tried to separate the putative U12 dependent introns from the U2 dependent ones with respect to their normalized scores. The lower thresholds for U12 type introns were empirically defined with respect to the minimum values of a reference set of minor introns which were used by Burge et al [5]. The test criterion we used was the same as in Zhu and Brendel [10], and as discussed by these authors it is likely to be different from the test statistic  originally used by Burge et al.

We also analyzed all predicted introns with respect to previously known consensus features of U12 introns as referred to by Russell et al [7]. In addition, we took into consideration that for effective splicing at the 5' splice site we require the sequence RTATCCTT where one of the Cs in positions +5 and +6 may be replaced by a T (Mikko Frilander, Helsinki, personal communication).

### Analysis of relationship between introns of *C. elegans *and *T. spiralis*

For identifying introns in *C. elegans *that are homologous to the U12 introns in *T. spiralis *the *C. elegans *genome sequence (sequence number 198, release of January 13, 2009) was retrieved from Wormbase ftp://ftp.wormbase.org/pub/wormbase/genomes/c_elegans/sequences/dna/.

A *C. elegans *protein database (number 198, release of January 12, 2009) with 23962 proteins was also downloaded from Wormbase ftp://ftp.wormbase.org/pub/wormbase/genomes/c_elegans/sequences/protein/.

BLASTX was first used to identify *C. elegans *proteins corresponding to the *T. spiralis *U12 intron genes. Genomic positions of the corresponding gene in *C. elegans *were then inferred using TBLASTN.

## Results and Discussion

We have previously examined the phylogenetic distribution of U2 and U12 spliceosomal components [8]. The results showed that U12 components were lacking in a number of phyla such as Nematoda, Choanoflagellida (Monosiga), Fungi, Mycetozoa, Entamoeba, red and green algae and Heterokonta. We have now examined the occurrence of U12 type introns in these groups.

### Identification of introns

Introns were identified by matching of ESTs to genomic sequences using BLAST [12]. The size distribution of introns for selected species is shown in additional file 1. Mode values vary between 51 and 95 nt for all species examined here. An exception is *C. reinhardtii*, which has a distribution of introns lengths with a mode value which is approximately 195 nt, consistent with previous observations [13].

**Figure 1:**
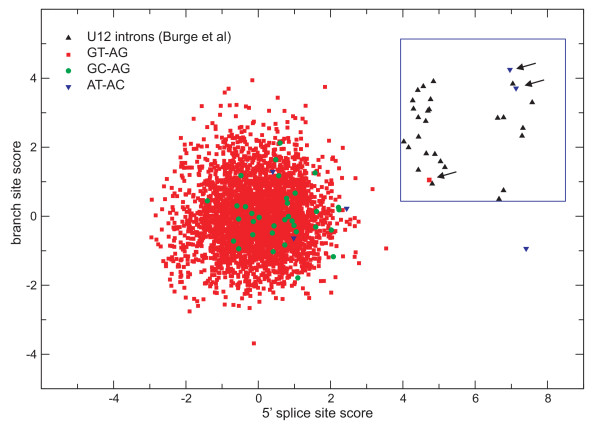
***Plot of splice site scores of introns identified in *Phytophthora sojae**. The scores of 5' splice sites and branch sites are compared to those of a reference set of U12 introns as used by Burge et al [5] (within blue rectangle). Introns in *P. sojae *predicted to be of the U12 type have both 5' splice site and branch site scores equal to or larger than the minimum values of the reference set. Arrows indicate one GT-AG and two AT-AC introns.

In order to discriminate between U2 and U12 introns we used methods described by Burge et al [5], Zhu and Brendel [10] and Sheth et al [11] as described under Materials and Methods. In the Burge et al method weight matrices were used to score 5' splice sites and branch sites. Normalized z scores for these sites were then obtained and used to produce plots like that shown in Fig. 1 for *P. sojae*. In order to discriminate between U12 and U2 type introns we used a cutoff based on a reference set of U12 introns as used in Burge et al [5]. Thus, for an intron to qualify as a U12 type both 5' splice site and branch site scores need to be at least the minimum values present in the reference U12 set of sequences [10]. The plot in Fig. 1 shows that in the case of *P. sojae *three different introns, one of the type GT-AG and two of the type AT-AC, fulfilled these criteria.

### U12 introns in *Trichinella spiralis*

We examined in more detail the prediction of U12-type introns in the nematode *T. spiralis*. Molecular phylogenetic analysis places Trichinella close to the root in the nematode tree, i.e. more deeply branching than species such as *C. elegans *of the Rhabditida branch [14-16] which is believed to be lacking U12 introns.

In *T. spiralis *we identified a total of 15402 introns. Out of these, 14866 were of type GT-AG, 218 were of type GC-AG and 8 were AT-AC type introns. In addition, 315 introns were identified with non-canonical terminal dinucleotides.

Using the method of Sheth et al [11], U12 GT-AG type introns (13) and U12 AT-AC introns (3) were identified (Table 1). Minor introns are thought to have the 5' splice site sequence RTATCCTT where one of the Cs in positions +5 and +6 may be converted into a T (Mikko Frilander, Helsinki, personal communication). There are 5 introns that do not conform to this rule, leaving 11 introns that are stronger predictions.

**Table 1:** U12 type of introns identified in T. spiralis.

EST	Genomic sequence	Intron length	B	R	Relationship to *C. elegans*	*C. elegans *orthologue	Gene function
	ATAC introns						
EX501652.1	AGA|ATATCCTTTC...TTGGGCATTGTTATATTTCCTTAACGGGTATGGTTTAC|GTT	2095	+	+	U12->U2	EEED8.7	SR (splicing factor)
	TyrIleGl nPheGlnGl nLeuLysAspAla						
	Ts TACATAGAAT...ACGTTCGAAGA GCTGAAAGATGCT						

	PheValAr gPheTyrGl nArgArgAspAla						
	Ce TTCGTTAG ATTTTATGAGT...AGACGTCGTGCTGCT						
EX500683.1	ATT|ATATCCTTTC...AATTTCATTTCCTTAACGTTAGATTTTTGTTGTTTTAC|TGA	94	+	+	U12 lost	E04D5.1a	NA
ES570647.1	ATC|ATATCCTTTC...GTATTGTTTGTATTTTCCTTAACTTCATATGTTTTTAC|GTA	182	+	+	U12 lost	Y37D8A.10	Signal peptidase complex, subunit SPC25

	**GTAG introns**						

EX499999.1	Ts GAG|GTATCCTTTG...TTTTGTTTTTCTCTCTTTTTACAATTATTATACAG|GCC	90	-	+	U12->U2	F10F2.1	PH, BEACH and WD40 domain-containing protein
	Ce GAG|GTTTGAAACA...TTTTAATATTGAACTAAAATTTTTGAATTTTCCAG|GCG	64					
ES570692.1	Ts TAG|GTATTGTTTT...TGCTACAAGGAATTTTTTTATTGCTTTGATTTTAG|AGT	617	-	-	U12->U2	F40F8.10	Small ribosomal subunit S9 protein
	Ce CCG|GTTTAGTTTT...AAGATTAGTATCGACTTCAAATTCTTCTCTTTCAG|TGT	291					
ES561213.1	Ts TCG|GTATTATTTT...CATATTAATCGTTTCATTTCTTAATGTATTTTTAG|TGG	54	-	-	U12->U2	ZC395.10	NA
	Ce CCA|GTACGTTTCG...ACATAGAATGAGTCGTAATTCGTAAATTTTCAGAG|GAA	150					
EX500486.1	Ts TCG|GTATTCTTTC...TAATATGTTTTTCTTTTTTTTCAACTTATTTTAAG|ATT	87	-	+	U12->U2	ZC328.3a	NA
	Ce CAT|GTGAGTTTCA...TCCTGAATTTATTCAAGTTTCAACCACATTTCCAG|CAT	758					
ES569928.1	ATG|GTATTCTTTT...ATTTCCATTACAAAATTACAACCGCGTTGTTCTTTCAG|TGC	107	+	+	Not known	Y82E9BR.15	Transcription elongation factor B
ES565768.1	CAG|GTATTCTTTT...CAAATTTTGGAAAAATTCTTTTTTTTTAATCCGAACAG|GTA	94	-	+	Not known	C34D4.4a	NA
ES562099.1	AAT|GTATCCTTAA...TGTATGAGGTTTGGTATTTCTGATTTTAATCATTTTAG|TGT	50	-	+	Not known	R07E5.14	RRM RNA binding domain) containing
ES563059.1	GCG|GTATCTTTTC...TATTTATAACTGAATCGTTTTTATTAATAATTTTTTAG|AGT	54	-	+	Not known	M04F3.4	NA
BQ738460.1	ACG|GTATCGTTCA...TCAATTTTTTTAAAAGTAATTTTCTTCATATATTTTAG|AAC	72	-	-	U12 lost/Not known	Y56A3A.36	NA
ES566079.1	TGG|GTATCGTTCG...ATTAACTAACACTTTGAAGTTGACAAGTGAATGTTTAG|GAT	140	-	-	Not known	M02B7.4	beta-1,4-N-acetylglucosaminyl transferase
EX500543.1	TCG|GTATTCTTTG...TTATTATTAATTTCTGTTTTTTTTGGTTTTCTAAACAG|AGA	86	-	+		None	
ES561535.1	GGG|GTATTATTTT...TTTTCTGTGATTTAATTGCATTTTAATGTTCTATCTAG|TGA	71	-	-		None	
BQ738918.1	GAA|GTATCTTTTA...TGAATTTTGCTAAATTGTACTTAACAGGTTGTTTTTAG|AAA	153	-	+		None	

Table shows all introns identified by the Sheth et al method [11]. Regions with best match to branch site PWM is underlined. For one of the ATAC introns (EST EX501652.1) is shown the shift in 5' splice site observed between *T. spiralis *and *C. elegans*. Rule of 5' splice site (R) is that 5' splice site sequence is RTATCCTT where one of the Cs in positions +5 and +6 may be converted into a T. Burge et al method (B) is described in [5]. NA = not available. For sequences of U2 and U12 introns listed in table, see additional file 2.

In order to study the fate of *T. spiralis *U12 introns we examined the homologous genes in *C. elegans *where U12 introns are believed to be absent. Of the 16 EST sequences that we identified using the Sheth et al method as being associated with U12 type introns, 3 had no matches to entries in protein databases and could not be associated with a *C. elegans *gene. Another 6 ESTs matched only partially to the homologous *C. elegans *gene and for this reason we were not able to examine the fate of the homologous *T. spiralis *intron. For the remaining ESTs we were able to compare the *T. spiralis *U12 intron to the corresponding *C. elegans *intron. Four of these had identical splice sites in the two species as shown in Table 1, and in all these cases the *T. spiralis *introns were changed from U12 to U2 type in *C. elegans*.

For two of *T. spiralis *AT-AC-type U12 introns the intron was completely lost in *C. elegans *(Table 1). It is interesting to note that in the case of the third U12 AT-AC intron, a shift from U12 to U2 is accomplished by a shift of splice site (Table 1, EST EX501652.1), such that the intron is moved a distance corresponding to three amino acids in the coding sequence. Therefore, we here observe yet another mechanism whereby a U12 intron may be converted to a U2 type intron.

Finally, we also used the method of Burge et al [5] to predict U12 type introns in *T. spiralis*. A smaller number of U12 type introns were found using this method; 3 AT-AC and one GT-AG U12 intron (Table 1). Also for other species examined, the Sheth et al method generated more U12 candidates as compared to the Burge et al method.

### U12 introns as predicted by requiring effective branch sites as well as 5' splice sites

**Figure 2:**
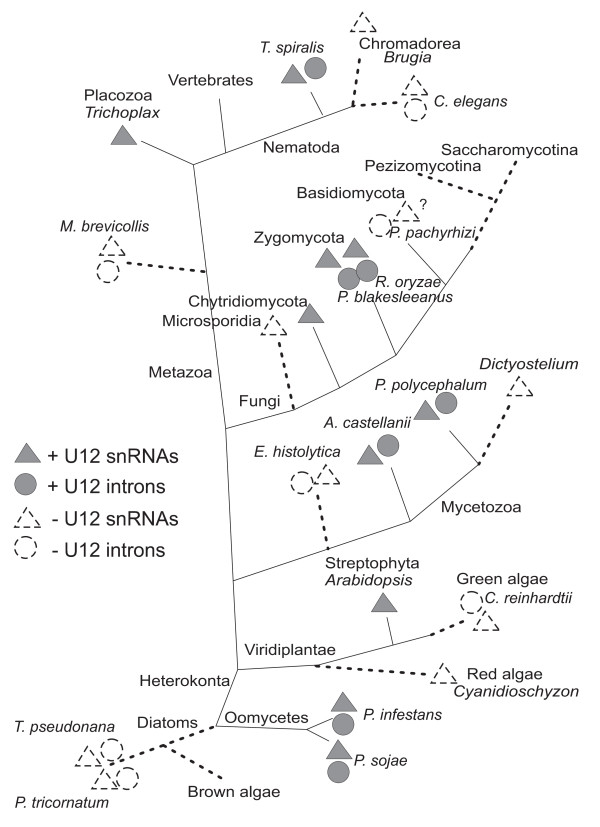
**Schematic phylogenetic tree showing instances where U12 introns were lost**. Presence or absence of U12 snRNAs and U12 introns are shown as well as paths where U12 splicing seem to have been lost (dashed lines). Figure is based on previous information regarding the phylogenetic distribution of U12 snRNAs [7,8] as well as results regarding U12 intron distribution described here.

In addition to *Trichinella spiralis*, we examined the introns in the choanoflagellate *Monosiga brevicollis*, in the Zygomycota *Rhizopus oryzae *and *Phycomyces blakesleeanus*, in the Basidiomycota *Phakopsora pachyrhizi*, in *Acanthamoeba castellanii*, *Entamoeba histolytica, Physarum polycephalum*, in the green alga *Chlamydomonas reinhardtii *and in the heterokonts *Phytophthora infestans*, *Phytophthora sojae*, *Thalassiosira pseudonana *and *Phaeodactylum tricornutum *(Table 2 and Fig. 2). Introns were predicted using the same methods as described above for *T. spiralis *introns.

**Table 2:** Summary of U12 introns identified in a range of eukaryotic species.

	Number of ESTs analyzed	U12 spliceosomal RNAs	U12 AT-AC introns (5' rule)		U12 GT-AG introns (5' rule)		U2 GC-AG introns		U2 GT-AG introns		Other AT-AC introns	
			B	S	B	S	B	S	B	S	B	S
*Trichinella spiralis*	25,268	+	3 (3)	3 (3)	1 (1)	13 (8)	217	217	14697	14685	5	5
*Monosiga brevicollis*	29,495	-	0	0	0	1 (0)	134	134	13327	13326	10	10
*Rhizopus oryzae*	13,313	+	1 (1)	1 (1)	2 (2)	3 (2)	71	71	5520	5519	3	3
*Phycomyces blakesleeanus*	47,847	+	9 (8)	8 (8)	8 (8)	12 (10)	446	446	13504	13500	31	32
*Phakopsora pachyrhizi*	34,394	-?	0	0	0	1 (0)	10	10	561	560	2	2
*Acanthamoeba castellanii*	13,784	+	1 (0)	0	1 (1)	3 (0)	21	21	1232	1230	2	2
*Entamoeba histolytica*	14,388	-	0	0	0	0	0	0	160	160	4	4
*Physarum polycephalum*	25,393	+	83 (14)	27 (15)	88 (57)	218 (109)	34	34	6452	6326	251	307
*Chlamydomonas reinhardtii*	202,044	-	0	0	0	0	532	532	25053	25053	7	7
*Phytophthora infestans*	94,091	+	2 (2)	2 (2)	1 (1)	1 (1)	66	66	6601	6601	9	9
*Phytophthora sojae*	28,467	+	2 (2)	2 (2)	1 (1)	1 (1)	34	34	3351	3351	5	5
*Thalassiosira pseudonana*	61,913	-	0	0	0	0	37	37	5140	5140	13	13
*Phaeodactylum tricornutum*	133,871	-	0	0	0	0	23	23	3815	3815	7	7

Table shows prediction according to methods of Burge et al (B) and Sheth et al (S) [11]. The "5' rule" is that 5' splice site sequence is RTATCCTT where one of the Cs in positions +5 and +6 may be converted into a T. Occurrence of U12 snRNAs is from Davila-Lopez et al [8]. For sequences of U12 introns as predicted by the Burge et al method, see additional file 2.

Previously, U12 introns have been reported in some of these species. Thus, Russell et al [17] reported three AT-AC and two GT-AG type introns in *A. castellanii*, one AT-AC intron in *R. oryzae *and one AT-AC intron in the peptidyl-prolyl isomerase genes of *P.sojae *and *P. ramorum*. It should be noted that Russell et al used a pattern based approach to identify U12 type introns. This method is not expected to be as accurate as the prediction carried out here which is based on position weight matrices. Two U12 introns were identified by Glöckner et al [18] in *P. polycephalum*, although it is not clear what method in that case was used to classify the introns.

In *A. castellanii *we identified three U12 introns (Table 2). One of them is the AT-AC U12 intron in the gene COMM7 previously found by Russell et al [17]. We also identified a GT-AG intron present in the gene for a mitochondrial carnitine acylcarnitine carrier protein. In addition, there is evidence of a GC-AG U12 intron, present in a gene encoding a lipid transfer protein.

**Figure 3:**
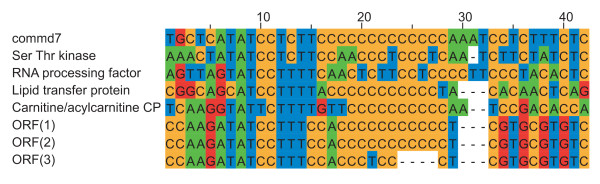
**Conserved sequence elements of U12 introns in A. castellanii**. Position 6 in alignment corresponds to the 5' terminal position of the intron. The majority of these introns were identified by Russell et al [7], whereas the introns of lipid transfer protein and carnitine/acylcarnitine carrier protein was identified in this work.

A multiple alignment of all available *A. castellanii *U12 introns, i.e. those identified previously [17], together with the two additional introns identified here, shows that there is a C-rich region in all these sequences downstream of the consensus 5' splice site sequence (Fig. 3). We do not know if this sequence conservation is functionally significant, but it seems specific to *A. castellanii*, as it is not found in other species with U12 introns such as *Physarum*.

In *P. sojae *we identified a previously reported AT-AC intron which is present in a gene encoding peptidyl-prolyl isomerase [17]. In addition, one other AT-AC as well as a GT-AG U12 intron in the ribosomal protein L31 gene is present in this species. In *P. infestans *we identified the introns homologous to the peptidyl-prolyl isomerase and L31 introns in *P. sojae*, as well as an additional AT-AC intron.

In *R. oryzae *we found the same AT-AC intron previously reported [17], as well as two additional GT-AG introns.

A very large number of U12 introns were predicted in *P. polycephalum *(Table 2). At the same time, this collection does not include any of the introns reported by Glockner et al [18]. Perhaps U12 introns are particularly predominant in this species. As an alternative, our prediction method may give rise to an unusually large number of false positives in *P. polycephalum*. On the other hand, many of the U12 introns may be regarded as strong predictions as they also conform to the 5' consensus rule. It is therefore highly likely that U12 introns exist in this species.

In summary, the phylogenetic distribution of U12 introns is entirely consistent with the distribution of U12 snRNAs [8]. This is illustrated in the schematic phylogenetic tree in Fig. 2. There are at least nine different branches that are associated with a loss of U12 splicing. When more genomic and EST sequences become available even more instances of such loss may be observed.

When comparing the distribution of U12 snRNAs and U12 introns one potential discrepancy is *P. pachyrhizi *where U12 introns seem to be missing but we have identified a U4atac snRNA. However, no other U12-type snRNA was found, and it would therefore seem likely that this species is missing U12-dependent splicing. The U4atac snRNA observed could be a non-functional remnant of the U12 splicing machinery of an ancestral species.

There is also a weak candidate for a U12 AT-AG intron in *T. pseudonana *(data not shown) but as this is the only U12 intron predicted in this species and because we have failed to identify any U12 snRNAs in this species the evidence of U12 splicing is so far very poor.

### U12 introns in ribosomal protein genes

Although U12 introns are very rare they occur in ribosomal protein genes in five of the species examined here, *R. oryzae *(S13), *P. blakesleeanus *(S13), *P. sojae *(L31), *P. infestans *(L13) and *P. polycephalum *(L4).

In the Zygomycota *R. oryzae *there are at least three non-identical versions of the S13 gene. These genes encode nearly identical proteins (see additional file 2). One of the genes has no intron at all and the other two have U12 GT-AG introns towards the 3' end of the coding sequence. In *P. blakesleeanus*, another Zygomycota, we have identified one S13 gene. This gene has a U12 intron in the same position as for the *R. oryzae *gene. More S13 genes might be found in this species once genome sequencing is complete.

By comparison the S13 gene in the Basidiomycota *P. pachyrizi *has two different introns and neither of them are of the U12 type and in the same position as the *R. oryzae *S13 intron.

*P. sojae*, *P. infestans *and *P. ramorum *of the Oomycetes group all have a L31 gene with a U12 GT-AG intron positioned towards the 5' end of the coding sequence. By comparison the L31 genes in the diatoms *T. pseudonana *and *P. tricornutum *seem to be missing introns.

The presence of U12 introns in ribosomal protein genes may be of significance from a regulatory point of view. Ribosomal proteins have previously been reported to be involved in U12 splicing. Thus, there is a U12 intron in the gene for ribosomal protein L1 in *X. laevis *[19-23]. This intron has a low efficiency in splicing, indicating that it might be involved in regulation of L1 expression. There is evidence that splicing of U12 introns is comparatively ineffective and is a rate-limiting step in gene expression [24].

The presence of U12 introns in ribosomal protein genes may also be relevant to the observation that in the yeast *S. cerevisiae*, where introns are rare, ribosomal protein genes is a predominating class of genes containing introns [25,26]. The splicing of introns in *S. cerevisiae *ribosomal proteins is presumably of great regulatory significance [27]. For instance, there is an autoregulatory mechanism of L30 where the protein inhibits the splicing of its own pre-mRNA [28]. There is also evidence that yeast ribosomal protein paralogues are different in terms of splicing regulation [29].

## Conclusions

The presence of U2 and U12 introns have been examined in a number species that we previously screened for U2 and U12 spliceosomal RNAs. In most species where U12 introns are found, such introns are very rare. The phylogenetic distribution of U12 introns is entirely consistent with the distribution of U12 spliceosomal RNAs. The currently available information on U12 introns and U12 spliceosomal components presents strong evidence that U12 splicing is missing in a number of phyla and species; in the Caenorhabditis branch, in Monosiga, in Microsporidia, in Basidiomycota, Ascomycota and Pezizomycotina, in Dictyostelium and Entamoeba, in the red and green algae and in the diatoms *T. pseudonana *and *P. tricornatum*. This would correspond to at least nine different occasions during evolution where U12 splicing seem to have been lost (Fig. 2).

We have examined in more detail the occurrence of introns in the nematodes *T. spiralis *and *C. elegans*. As these two species are relatively closely related by evolution they offer a unique possibility to monitor the process in which U12 splicing is lost. By comparing *T. spiralis *U12 introns to their homologues in *C. elegans *we noted that U12 introns were eliminated using different mechanisms. In some cases U12 introns were lost completely. Other U12 introns were subject to extensive sequence changes including changes in the 5', 3', and branch site regions of the introns. In one case a U12 to U2 conversion was achieved by shifting the splice position only a short distance.

## Authors' contributions

TS conceived of the study and drafted the manuscript. SB carried out bioinformatics analyses and helped to draft the manuscript. All authors read and approved the final manuscript.

## Supplementary Material

Additional file 1 **Intron length statistics**. Upper panel: Distribution of lengths in the size range 1-300 nt for all introns (U2 and U12) of *T. spiralis*, *E. histolytica*, *A. castellanii*, *P. tricornutum*, *M. brevicollis*, and *C. reinhardtii*. Lower panel: Mean intron lengths for all species with U12 introns considered in this work. Click here for file

Additional file 2 **Sequences of introns**. Sequences of introns referred to in Tables 1 and 2. Sequences of *R. oryzae *ribosomal protein S13 genes and introns. Click here for file
