# Supplementary material for: U12 type introns were lost at multiple occasions during evolution
Source: BMC Genomics. 2010 Feb 11;11:106. doi: 10.1186/1471-2164-11-106 (PMC2846911; doi:10.1186/1471-2164-11-106)
Supplement: Additional file 1 — Intron length statistics. Upper panel: Distribution of lengths in the size range 1-300 nt for all introns (U2 and U12) of T. spiralis, E. histolytica, A. castellanii, P. tricornutum, M. brevicollis, and C. reinhardtii. Lower panel: Mean intron lengths for all species with U12 introns considered in this work. [file 1471-2164-11-106-S1.PDF]

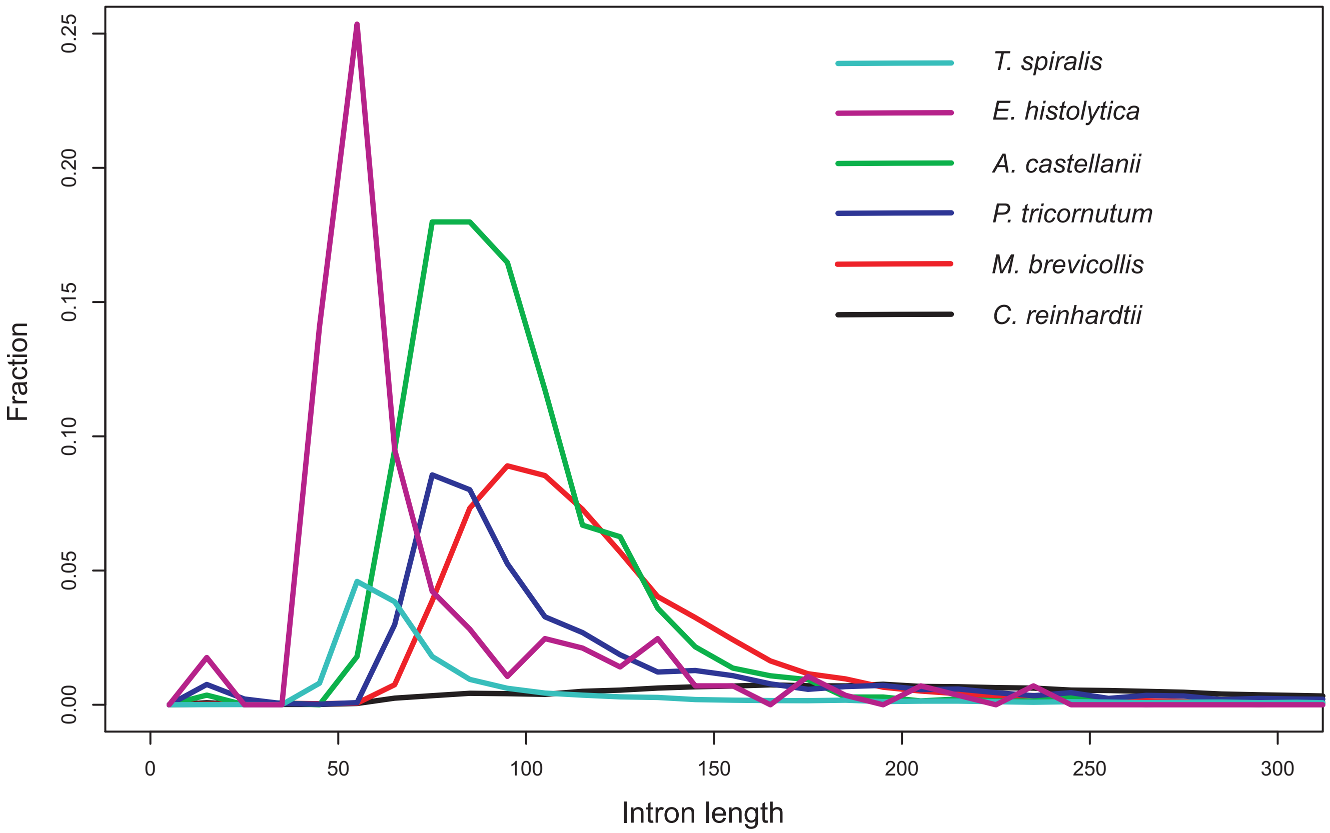

	Mean intronlength	
	Arithmetic	Mode
T. spiralis	253	63
C. elegans	338	51
M. brevicollis	132	95
R. oryzae	103	56
P. blakesleeanus	104	70
P. pachyrhizi	95	80
E. histolytica	139	55
A. castellanii	97	75
P. polycephalum	186	55
C. reinhardtii	266	200
P. sojae	101	80
P. infestans	116	73
T. pseudonana	127	85
P. tricornutum	139	81

Intron length statistics. Upper panel: Distribution of lengths in the size range 1-300 nt for all introns (U2 and U12) of *T. spiralis*, *E. histolytica*, *A. castellanii*, *P. tricornutum*, *M. brevicollis*, and *C. reinhardtii*. Lower panel: Mean intron lengths for all species with U12 introns considered in this work.
