# Supplementary material for: U12 type introns were lost at multiple occasions during evolution
Source: BMC Genomics. 2010 Feb 11;11:106. doi: 10.1186/1471-2164-11-106 (PMC2846911; doi:10.1186/1471-2164-11-106)
Supplement: Additional file 2 — Sequences of introns. Sequences of introns referred to in Tables 1 and 2. Sequences of R. oryzae ribosomal protein S13 genes and introns. [file 1471-2164-11-106-S2.PDF]

## Additional data, Bartschat & Samuelsson "U12 type introns were lost at multiple occasions during evolution".

### T. spiralis introns of Table 1.

Intron sequence with 8 nt of flanking exons.

```
>EX501652.1_Contig0_12048831_12050941_T._spiralis_U12
PWM=thaliana_atac_U12;5'ss_score=98.9937;bsite_score=86.1026;3'ss_score=47.6958
TACATAGAATATCCTTTCAATTATTACATTTTATTGCAGGAGGTTGAACTGATAAAGCATTTATGCTTAGCAATGTAAAT
TGATTATTTTCTTCAAATTTTTTGCATTTTTATGTTCAGATGAAATTTTTTTTTGTTTTCATTACTTCACATTTCATTCA
GATTTTACTTACTTATTATTCCTCAACACTAATAGGATAGAGCGTTTTTATCACAATTTCGATTGTTTAGATAGTCTGCG
CTAGTAATTTCAAATCACTAGGCACTCGCGTAGTTTATGGTCAGTTATTTTTTCGTGCTTTTCATCCTAGGGGCTTTGCG
TGAATCTTCGCATTGCTACTATTGGCTGAACTAATTTTTTGGTTCTAGCAAAAGGGCGTTTAAAAGACGTAAAGCGAGAA
TGAAAGAATTTCTTTCGTCAATTTGTTGGTGTGTAAAGTAAAAGATTCGCTCGCTCGCGACTAGTGACAAAAAAGTCAAG
ATGCAATAAAGTCAAAGACCAAGACTAAAGCATACTTTAAACCAGAAGACAGAGACAAGAAAGATAACTATATTTAAAAG
CTTTTGGCAATCAGGTAGCAGCCCAAAAATTTCTGCTGTTTACTTTCCACAACAAAAGGGGGAAGTGATAATAATTAGCT
TCTTACTGTAATTAGGATAGGTGAATTATATTGATAACATAATAGTGTACAATGTAAATAGTTTACATAAGCCTCGTTTG
GGTATATCGTGCGTTTGGTCGGTTAATTATTTGCTGCAACATTCACAGCAAGCTTGTCTTGTGTAATTTAACTCTATGTG
ATGCATATTCAATCGAAATGTTCATTTTAAATGAAACATCGCAAAATTATGTTTAAAACGAGCAGAAATTAAATGACTAT
TTTATAAATGCATATTCAAATGAAAATTATGAATGGCATTCAATTTTATTAATTTGTATTTGTAATCGAATCACCATAGA
ATGTAAATGTTATACAGAAATTTAAATTGTAGATTGTTTGAGAAACTTTTAGAAATATATTTCTTGATTGATATTTTCTG
ATGAATCTGCTTTTGTAATTGTTATTCGGGTTCCTTTTTGTTTGTGGGTTTTTATTGGTATCAAACATGAAAGCATTGCA
AGATGGTTTATCCGTGATGTAAACAATAAATAGTACTGTACTGTACTTGTGTTTTTCGATTGAATTGCAAGCGGCTGTTC
TTTGAATTGCGTGGGTTTTGATGTTTGTTGTTTTGGCGATATAAAATTTTCTTTTGAAACTTTTCGTTGACAATGTAAAA
ATCGAAGAAATCATTAGGATGACACTAATGATAATTTAATCACCTTTTTATCATTTGTTTGACAGCTGGTGGTGTTTATT
TAATTTCGATAATTTTACGAAGAATCCTGCTACCTGATGCAAATGTTCATTTTACATCATTTTTATTTAGGCCTTTATCG
TTGTTAATAATCATTTTAATCGTAAAATTGCAGAGTAGAACTTATGTAATATGCGGTGACGTCTAGCGGTTGTTTTAAGA
GGTACATAGAGATTGAATTGTGGGATTGAAATGCGGTGAATGGGCGCATATGGAAAAAAGTTTTATGAAATATACCTTCA
TTATATAAAAATTTTAATAAAACAAGTATTATTATGAACGATTTCAACGAATAATTTTCTTTCTTTCCACAAACAAATTG
TAATTGGTCTGATTTATTTTTTAGTTGGTTGTTTTATGTGGTTTTGTAGATTATTAATTTGTTTTAATGTTAAATTCGTA
GAGATTGTAATTTTTTATATTTCAAAGTGTTTTACATAAGCATAGCACATATAATAATTGCCGCTGGAAACATAGAATTC
TTTATCCGTAATGTATACATATGTTATTGCATTTCATTGATCTATTTATGTGATGGGTACAATGTGGAAGTTATTTTTTT
TTACGAAAATACCTGCATTTCAAGCATTATGGTAATGTTTATTACGTAAATTTGAAAAAAGAATCGTGCAGTGGGTAAAA
CCTGAATGAAATGTGTAGTATGAAATCTTTTTTTAATTATTACCTAGCAGTTATCGAGTTAGAAATTGGGCATTGTTATA
TTTCCTTAACGGGTATGGTTTACGTTCGAAG

>EX500683.1_Contig2.221_53422_53531_T._spiralis_U12
PWM=thaliana_atac_U12;5'ss_score=98.5856;bsite_score=86.1026;3'ss_score=66.0626
TGGAAATTATATCCTTTCATTGGAATAATTTTTGTTCTATTATAATTAAGTTTATTATTTGTGAAATTTCATTTCCTTAA
CGTTAGATTTTTGTTGTTTTACTGATGTTC

>ES570647.1_Contig9.9_26835_27032_T._spiralis_U12
PWM=thaliana_atac_U12;5'ss_score=99.4490;bsite_score=100.0000;3'ss_score=80.6784
AGCGTATCATATCCTTTCAATATTTCTTAATTGGTTGTTTTATTTTTATTTAATTTGTAGAGTAATTGGTTGTACAATTT
AAAATTAATTTTGTTAAGTTAATAATTTAATGCATTTTTAATATTTTAATTATATTGATATATTTAATGAACGTATTGTT
TGTATTTTCCTTAACTTCATATGTTTTTACGTACTTCG

>EX499999.1_Contig5.54_18621_18726_T._spiralis_U12
PWM=elegans_gtag_U12;5'ss_score=99.8811;bsite_score=55.4023;3'ss_score=70.9839
AATTTGAGGTATCCTTTGCAATAGTAGCAGTTGCTGTTGTTTCTTATATTTAATAATTTTCCATTTTGTTTTCTCTCTTT
TTACAATTAATTATACAGGCCATGTG

>ES570692.1_Contig20.24_20539_21171_T._spiralis_U12
PWM=elegans_gtag_U12;5'ss_score=86.7650;bsite_score=61.9733;3'ss_score=67.1087
```

CACATTAGGTATTGTTTTAATTATCTAAATTTAATTAGCCTTATACATGATGCGTAGTTGTGTTGGTGTTATGAGATTTT
TCTAATTCTTTTGAGTTCTAGATTTCAAGTTTAATACGTAATTGATTTTCTGCATTTCCACAGTATCTACTATTATAAAT
TTGAACATCAAAAAGTTTTATACTATTGAGAAATTGTGGTTCTTAAAAGGTTTTGCATGTATGTTTATTTAATTTTATAT
TTTTGTCTGCACTGCGTTTTAGTGTCTACATTCTGTGACGAATATTATTTTGAAATTTATCGTAACAGCTAGGCATAATT
TGTTGTGTATACTGTAGAATTTTTTTAGAATAATATTCTTTATTGAATGAAGATTTGTTTTTCAAATTAGCAGTATGCTT
TTAAGTATACTTGTAATAAATGGTGTTTAAGTGGTGTGATTTTTTATTTGAAATGCTATTAAATTGGTAATTGTTTTGTG
TACAGGTAATTATTTAATTTAAATATTTTTTCATTTTGAAGGTAACTATTTTTGTATTTAATAAATTATTGTTAGGAAAG
TTTGATTGCATGACAAATGTACATTAATTTTGCTACAAGGAATTTTTTTATTGCTTTGATTTTAGAGTTCGCC

>ES561213.1_Contig0.115_85128_85197_T._spiralis_U12
PWM=elegans_gtag_U12;5'ss_score=83.7114;bsite_score=66.0636;3'ss_score=53.3456
ACTTTTCGGTATTATTTTTTTCTTAATCATATTAATCGTTTCATTTCTTAATGTATTTTTAGTGGTATTG

>EX500486.1_Contig2_1632610_1632712_T._spiralis_U12
PWM=elegans_gtag_U12;5'ss_score=87.7398;bsite_score=63.9316;3'ss_score=74.5985
TGTAATCGGTATTCTTTCAAGCAAACTTAAATTGTTAATTATTTTTTGTGAAATTATTATTAATATGTTTTTCTTTTTTT
TCAACTTATTTTAAGATTATTCG

>ES569928.1_Contig12__1086033_1086155_T._spiralis_U12
PWM=droso_gtag_U12;5'ss_score=93.0588;bsite_score=62.5919;3'ss_score=45.6795
TGAAAATGGTATTCTTTTTCTTTTTCTTTTTTCTTTTTGCTTTTGACGACATCCTTGATTGTGATTGTTATATTTAAATT
TCCATTACAAAATTACAACCGCGTTGTTCTTTCAGTGCCGGAT

>ES565768.1_Contig0.199_40940_41049_T._spiralis_U12
PWM=elegans_gtag_U12;5'ss_score=91.9080;bsite_score=72.3313;3'ss_score=45.5938
CGATGCAGGTATTCTTTTTAAACTCCTTTTTTTGTTATTAATCAGAAAGCAAACATAATTTCAACAAATTTTGGAAAAAT
TCTTTTTTTTTAATCCGAACAGGTAAACTG

>ES562099.1_Contig9.2_28558_28623_T._spiralis_U12
PWM=thaliana_gtag_U12;5'ss_score=89.8635;bsite_score=53.2352;3'ss_score=66.6291
CCGACAATGTATCCTTAATTTGTATGAGGTTTGGTATTTCTGATTTTAATCATTTTAGTGTCTGGT

>ES563059.1_Contig4.102_38894_38963_T._spiralis_U12
PWM=elegans_gtag_U12;5'ss_score=86.9885;bsite_score=65.0223;3'ss_score=82.8339
TTTCAGCGGTATCTTTTCCATATTTATTTATAACTGAATCGTTTTTATTAATAATTTTTTAGAGTTGATG

>BQ738460.1_Contig4.93_17508_17595_T._spiralis_U12
PWM=elegans_gtag_U12;5'ss_score=78.9252;bsite_score=63.0737;3'ss_score=82.4052
TCTGGACGGTATCGTTCATCTTTGGCATTGTATTTGTTTATTTCAATTTTTTTAAAAGTAATTTTCTTCATATATTTTAG
AACCACTT

>ES566079.1_Contig0_9645545_9645700_T._spiralis_U12
PWM=elegans_gtag_U12;5'ss_score=82.3137;bsite_score=47.2176;3'ss_score=48.3711
TTATCTGGGTATCGTTCGTTGATTTTAGAAAGTTATTAAAATTATCACGCTTTGTTGTGATAAAATGATTTTTATGGCTG
TTCTTTGAGTTTCGATTTTTCTTTTTTCATATTAACTAACACTTTGAAGTTGACAAGTGAATGTTTAGGATTTCAA

>EX500543.1_Contig0_9934034_9934135_T._spiralis_U12
PWM=elegans_gtag_U12;5'ss_score=86.6273;bsite_score=62.1857;3'ss_score=45.5727
CCGGATCGGTATTCTTTGGATAATTTGCTTTATTTATTTATTTATTTATTAGATAGTTATTATTAATTTCTGTTTTTTTT
GGTTTTCTAAACAGAGAAGGAT

>ES561535.1_Contig9.57_957_1043_T._spiralis_U12
PWM=elegans_gtag_U12;5'ss_score=81.2776;bsite_score=60.5457;3'ss_score=44.4623
GCCCTGGGGTATTATTTTTTGAAAACATAGGATAAATTTTGTTTTCTGTGATTTAATTGCATTTTAATGTTCTATCTAGT
GAACAAC

>BQ738918.1_Contig0.155_7427_7595_T._spiralis_U12
PWM=elegans_gtag_U12;5'ss_score=80.6470;bsite_score=73.9177;3'ss_score=62.1737
TATATGAAGTATCTTTTATTTTTTATATGTACAATTTCATTTTTGTTTGCTGAGAGAATTTGAATTTAATGTTATAAAGC
TTTTGTTTTTCGATGTTATTGTGAATTCTGTATTTGCTGAATTTGAATTTTGCTAAATTGTACTTAACAGGTTGTTTTTA
GAAATGCTG

**C. elegans introns of Table 1**

Intron sequence with 8 nt of flanking exons.

```
>ce6_dna range=chrIII:4604977-4605040 C. elegans U2 homologous to EX499999.1
AGTTTGAGGTTTGAAACAACTTTTTAATATTGAACTAAAATTTTTGAATTTTCCAGGCGGAATC

>ce6_dna_range=chrII:11125543-11125833_C._elegans_U2__homologous_to_ES570692.1
PWM=elegans_gtag_U2;5'ss_score=78.9040;bsite_score=44.8683;3'ss_score=73.6861
CACATCCGGTTAGTTTTTGTTTCTGTATAAAAATATATTTAAAAATGAAACAGTTTTTAGAACTATCATTTTAACATTAA
ATTCATCTTTCGGAAAATTGATTGTTCTGGCAGTTAATTTTTTACAAATGTTTTTCGATTTAAACCAGTTAGGTATTCCT
GCGCTATTTTGAAGACTGATCCAGTAGTCATTGTCCGGAATATTTCATTAAAAAATTTATTTCACTGAAAGATGACATGA
CAAATTACAAGATTAGTATCGACTTCAAATTCTTCTCTTTCAGTGTCCGCA

>ce6_dna_range=chrIII:5274455-5274604_C._elegans_U2__homologous_to_ES561213.1
PWM=droso_gtag_U2;5'ss_score=61.9188;bsite_score=49.4161;3'ss_score=70.1089
CACTTCCAGTACGTTTCGGATTAATATTTCGATTTCTATTTTAAATGTTTAAATACGATATGAAGCATATTTCTTGCTTC
CTAAATATTCACAGAATGTTCATGATGGTACATAGAATGAGTCGTAATTCGTAAATTTTCAGAGGAAGCA

>ce6_dna_range=chrI:6414790-6415547_C._elegans_U2__homologous_to_EX500486.1
PWM=elegans_gtag_U2;5'ss_score=74.2514;bsite_score=73.9261;3'ss_score=74.1659
TTTAGCATGTGAGTTTCAGAACAATTTTTTATTTTATTTTTTCAATTGATTTAACAAAAAAATGGTTTTCCATTTCTTCA
TATTTCGTTACTGTAATTCTAAAGGAATCAAAAGAACACTCAATATTTTTTTTGTTATTCGCAAGGATACAACTCTCTTC
AGAAATATCCAATTATAGACAGACAGTGGAAGTCACTGCCTGGCACCAAAGCACAAAGAATCAGAATTAAAGTCTAGTTG
GAAATCTTGTAGTATTTTGAAAGTTTAAATAAAAAAGAAGCAAACATATGAAAATTATAAACCCATCAAATTACCCTAAA
AGTACCTAGGCATACCTTGGAAATTTGTAGTAATTATTCGCACGAACATTTAAAAATTATCTTTTTTCTGAAATCCTGAA
AAAATTTAGATATGTAATAACCAGACGCTGACCGCGCCTCCGGCGCATCCAACTGGCTGCAATAAATTTCAAATACCCCT
TGAAAATAACATTGAAATTTTGAAATTAATTTGATTTTTTTGAAAATGAAAACTTTCAAAGTAACGGTAATTTTGCGTGT
TGGCATAGCCTACATTGGACGGAAGGAAACTCTACTTTATATATAGTAAATGATTTTTACAAGAATTTTTACAAGAAAAC
AAAATGATACCTTTTTGGAATAATATACGTACCGACACCCTTAATTGTACCTTTAAGAGAATTTACGACATAATTTCCTG
AATTTATTCAAGTTTCAACCACATTTCCAGCATATTCC
```

**Introns of Table 2 predicted by the Burge et al method.**

Intron sequence with 10 nt of flanking exons.

>p.sojae_AT_AC_scaffold_6_323930,324051_2
PWM=homo_atac_U12;5'ss_score=6.9526;bsite_score=4.2511
CGGCCTGGGGATATCCTTTTCACCAAATACTTTTGTGTTGTTGGTAGCTGCATTTTACTAGAGTAAGGACACTTTTGATC
CATGCTGCTTCCTTAACCGTGACGTTGTGTACAATTCAGCCG

>p.sojae_AT_AC_scaffold_1_919762,919889_1
PWM=homo_atac_U12;5'ss_score=7.1443;bsite_score=3.6854
GACCTGTTCGATATCCTTTCACCTCCATTGTTGCATGCGTGTACCTGAAGTGCATGTCCAAACACCCTGTTGATTGATAC
TGAGGTTCTCCTTAACCTGTATTTGTTCCGTCTAACACGAATGTGACG

>p.sojae_GT_AG_scaffold_82_122198,122382_2
PWM=droso_gtag_U12;5'ss_score=4.7303;bsite_score=1.0509
TTCACGGATGGTATCCTTTTTCCACCCCCTTTTGCATATATCGCACTGCATACTGCAGTGCCTGCATATTTTCCCCCACT
CGGTTCGCCGTAGCTCCCACCTTGGAGACTTCCTTGACTTTCTCTCTGCTCTGCGTTGCTCCGTGGCTCCCGCTTGCTAT
TGCGTGACCTCACAGCGTGTTTAAG

>r.oryzae_GT_AG_supercontig_3.5_3082913,3083084_1
PWM=droso_gtag_U12;5'ss_score=5.0250;bsite_score=5.2044
CCTGGAAATAGTATCCTTTTTTGATTATACACTAACATTTCTTAGAGAGAGAGCGTGTATCCATATTTATTGCCTATATT
GGACATTACTTGGAGTTGCTTTTTCGAAGAAAAACTGCTCGGGTAACTGCCCTCCTTAACCCAATTCTTTGTTTTTTTAT
AGTGAGTCTGCT

>r.oryzae_AT_AC_supercontig_3.3_2725639,2725736_2
PWM=homo_atac_U12;5'ss_score=7.5652;bsite_score=4.0268
GGTTATAAACATATCCTTTTTTTTTTAAAAAAAATGATAAATACTAACAAACTATGAGACTATGATTCCTTAACTGTTTA
ATATATACTGAGGAAATG

>r.oryzae_GT_AG_supercontig_3.10_1679086,1679252_2
PWM=droso_gtag_U12;5'ss_score=5.0250;bsite_score=5.2044
CCTGGAAATAGTATCCTTTTTTGATTATGTACTAACGCTTTAGAAAGAGAACGTGTATTCATTTTTTTATTGCCTATATT
GGACATTACTTGGAGTTGTTTTAGAAAAACTGCTCGGGTAACTGCCCTCCTTAACCCAATTCTTTGTTTTTTTATAGTGA
GTCTGCT

>p.infestans_AT_AC_supercont1.2_722204,722309_1
PWM=homo_atac_U12;5'ss_score=6.7999;bsite_score=3.6955
CGGCCTGGGGATATCCTTTTCCGCCAATTAGTTGCATGAATGTGGGGTGTGCGCTTTAATAATGTCGTGGGTTCCTTAAC
TGTGTGGGATGTGTACAATTCAGCCA

>p.infestans_AT_AC_supercont1.9_3719549,3719679_2
PWM=homo_atac_U12;5'ss_score=7.4344;bsite_score=3.5086
ACAACTATTCATATCCTTTCTTCCTTCACACTGTCTAAGTTGCAGCTTATGGACTGTCAAGTGCATTTTCAGATGTGAGT
TAAGGACTTGGATAGCTTTCCTTAACGAATGGAGGTTATACGTTTTGCAGA

>p.infestans_GT_AG_supercont1.28_1588557,1588728_2
PWM=droso_gtag_U12;5'ss_score=4.5735;bsite_score=0.7654
TTCACGGATGGTATCCTTTTTCAACCCCTTTACATGCATATGCTGCACGTTATCTGCGTATTTCCGCACTCGGTTCGCCG
TAGCTCCCACCTTGGAGACTTCCTTGACTTTTTCTCTGCTCTGAAATTTGTTTACAATGCGGTTCCTCTTGTGACCCAAC
AGCGTGTTTAAG

>p.polycephalum_AT_AT_Contig7165.2_996,1271_1
PWM=homo_atac_U12;5'ss_score=2.2721;bsite_score=0.8366
GACCAACCTGATATCCCAATAAAATGAGAAAAGGGTAGCGATAAATAGATATAGCGAGGAGGAGGATGATTATGTACTAG

TTAGTCCCATTCTTCTATGCTTGGGCAGGACTACGCCGTAAGTTGTGCGCATCCTTCCAAGAGTGGATTTGACTAGCTGA
AAAGTTCTGCTGGTTTGATCTTCCGCGCGAGGAAGAGAGCGCGGCGTGGTCCTGGTAAGGCATAGAAAAAATTTGCATCG
TAAGTTAGCCATTTGACAAACCATATATATCAAGGC

>p.polycephalum_AT_AA_Contig5987.3_1834,1912_2
PWM=mus_atac_U12;5'ss_score=3.2587;bsite_score=0.6911
TTATGCGAGAATATCCTCTTGACAAAAATACATTCATCCGTATTAAAATTGAGATTGACGCATCCTAAAATATCCATCC

>p.polycephalum_GT_AG_Contig5319.4_1661,1883_2
PWM=droso_gtag_U12;5'ss_score=2.0919;bsite_score=0.6583
TTGCTAAGATGTATCCTTTTTTCACATACACATTCCTTGCGTAACATAAATTTTATTTGCATATTTGCGCACTTATAATG
TTTGCCTACATTCAATGATGATTTTTTTATTCGAGCGTTTTAACCCTGAAACCACTGATGAAAGAATTTCTGAGCACAAT
TCATCACACCTTACACCCACCACATCACCATCTTGTTCTTTGATAGCACGTAGCCGCGTTGTC

>p.polycephalum_GT_AG_Contig3281.6_599,704_2
PWM=homo_gtag_U12;5'ss_score=1.6318;bsite_score=0.5496
CCAATTGCGTGTATCCTATTATTTGTTTCATGCATTTTTTTAATCCATTTATTAATATAAATTTCCTCAAATTTAAATTT
TTTTAAATACTATAAGGCATTGATCC

>p.polycephalum_AT_AA_Contig2162.8_1197,1631_1
PWM=homo_atac_U12;5'ss_score=2.9364;bsite_score=1.8456
TTTATTGATGATATCCTATACAAATAAATTAATGAGAAGATGGCGTACTTACTAACTTAGATTTGAGCTAAAAATATGTT
GGGCGAGCCGAAAAACAATAGTCGAGACGAAAATGAATTAATTTTGATAAATTGAGGTAATTTCAAACGCAGAAAACTAA
CTGGCCTTAACCCTTAAGAGTACAAAAGGTGACAAAAGCCCAACAAAAAATTCGGAAAAAAATTGCGCGAAACGTGCCTA
GTGACAAAGGGCCAGTTTGTACTTTTCGCGAAAAGTTCGTTTGTTGTACGCTTAAGGGTTAAGTTCTAGGCCTAGAAAAT
GGGCACTTACGCCTTGAAATTACCTCATTGAAATAGCGAATTCAAGAGAAAGAGGAACAAAAAAAGTTTAAGTGACTTTA
TTATTTTTCCTTTAACTCTACAAAAAGTTCAACAA

>p.polycephalum_GT_AG_Contig3184.3_2480,2795_1
PWM=mus_gtag_U12;5'ss_score=1.9906;bsite_score=1.4381
TACCAAATCAGTATCCTTTGTTCTTTCGTTATTACCTCTTTCCAATTGAAGGGAAAAAAATAAATGGCCGAATTTACTTT
TTAGAATTGCCTACGTAGTCTGCCGCAGGTATTTGTGCTAAATATGCGGCTGAAATTTTTCTTTGCAATTCGCAGATTGT
CAATCTTCCCTTTCTCCAAACGATTTAAGCGCGAGGAGCATAGGTGCGCTTTTATGCGCTAGATATGGATCTTATCTCGT
ATACTTTCCCTACGCCTACCCTCCTCCTCTACATTCCTTTTTTCCCCTCTCTTTGATCATATTCAGTTATGGGGGC

>p.polycephalum_GT_AG_Contig1208.4_1862,1949_2
PWM=homo_gtag_U12;5'ss_score=1.7090;bsite_score=1.5393
TTGTCCTTATGTATCCTTACGCCACTTTTACGTTCACAACACCTAAACAAACACAAATATTTCTATCACCCTACATAGAT
ATCCAATA

>p.polycephalum_AT_AA_Contig4.11_11020,11417_2
PWM=homo_atac_U12;5'ss_score=2.8649;bsite_score=0.8166
CAAGTAATGAATATCTTTACTTCACATATACCTACCGTCATATACCTTCATACTTCACAAACCCTCAATAAACACTCAAT
CACTATATTATGTGAGCCTCCTGCACTGGAATTATAACTATATTTTCAAAAGAATCTAGTTCAAAATTATCATTCCGCAC
TTCCTTTCCACTACCCCCTCCCTTTTTCACACTTTCATTGCTAATTACGTTCACTGAAACCTCATATGCTCTTCGACAAA
TCATATATACACCTATGGATTAAATAGAAGCCCAAGAGCAATTGTAGAAGTGGATATAAGCCCTGATCTGTTGTGCTCGC
CAAAATTAATATTCTTTCATTTAGTTATATTTTCCCCAAATATATTTATTTCTTTGACCTGAATTTAATCACATATAT

>p.polycephalum_AT_AC_Contig3199.3_3567,3707_1
PWM=homo_atac_U12;5'ss_score=3.6794;bsite_score=0.8960
AAATAGTTGCATATCCTTTTTTTTTTTTTTTTTGTTTTTTTTTTGTTTGTTTTTTTGCTTATTTATTTCCTTTATTTGCTT
ATTTATTTCATTTATTCCCCTCGCTCTTTCATTATCGTTGACGTTGCACACATTTCAAGAA

>p.polycephalum_AT_AA_Contig4964.3_2075,2154_1
PWM=mus_atac_U12;5'ss_score=3.4470;bsite_score=0.6609
GTCAACTTCAATATCCTTGCAGCTACATAGAAAACGTTTACTGTGCGTAGTTCCTTTTGATAATGAGAAAATATTGCAAA

>p.polycephalum_GT_AG_Contig1013.10_1594,1934_1
PWM=droso_gtag_U12;5'ss_score=2.1451;bsite_score=0.5403
CGGAAAACCCGTATCCTTTTACGATCGTGCGCAAATCGTTCCATTTTTGACAGATTTCGCTGCAAAAATTCGTCCTACAA
AATTTGCGTCTTTCGAGAATCACGTCTACAAAATATCAAAAATCGTGTACTGCGAAAACCGTCCGTCAAAAATCGCGTCC
TACAAAAATTACGTCCTACAAAAATCGCGTACTACAAAAATTGCGTCCTACAAAAATCCAAAATTCACGTACTACAAAAA
TCACTGCCGAACGGATAATTGCGTTTTTTGCGAAATTCCGATGCCTCTTTTTCTTGTACACTATGCATATAGGATTTCTT
GATAAAATTAGGTTTTCAACT

>p.polycephalum_GT_AG_Contig1169.4_3736,3855_1
PWM=homo_gtag_U12;5'ss_score=1.9032;bsite_score=0.8062
TAGGTCACCTGTATCCTTCCTTTTATCACAGTTCACCATTAATCCTTTAATCTTTCTCCATAAAAATTTTCGTTCTATTA
ACTCCCCAATTTTGGTTAATATTACCGTAGAAATATTTTA

>p.polycephalum_AT_AC_Contig4230.3_2202,2338_1
PWM=homo_atac_U12;5'ss_score=2.1547;bsite_score=2.2187
GAAAGTGTTGATATCACTGGGTTTTTTTAAATCATTTATATTGCGGTTGCTATTTTCATTGTTATTTGGTATTTTTTTAA
AATTCATTAATGATCTACACTTTCCTCTTTACCTTTGACTGTAGAACATGCACAATT

>p.polycephalum_AT_AC_Contig2873.5_138,522_2
PWM=homo_atac_U12;5'ss_score=2.9724;bsite_score=1.8183
GGCGAAGTATATATCTTTTTTAAAAACAAGTTTGTGCATTTATCGGTAGAGTGTGTTTATAATACTTTACTTCGCTGTTA
TTTTGGCTCAGTTATGTCCGTTTTTGTTGCAATTATTATTTAGTAATTTATTTGGTAAATTTTTTGGTAATTTTTTGGTA
AATTATTGTGGGTAAATTTTTAGTTACTATATTACTAACGTTCACATAATTGCCATAATAGCTGTTTACATAATTATACA
TTAACATAATAATTACATGAACATAATTTATAAGGCAAAGCAATTGTTTGCTTTTAATCATTTTAAATTATTACTGACCC
ATCTCCCTCCCGCTCCCCTCCCCATAATCCTCTGATTTCATTTGATTTTGAATACATATGTATAA

>p.polycephalum_GT_AG_Contig5363.3_932,1228_1
PWM=homo_gtag_U12;5'ss_score=1.5169;bsite_score=1.0239
TTTCTTCCTTGTATCTTTTTTAATTCAAATATAAGGTGCATAAGCTGCAAGAGCGATTGCGTGGATCAAAAAGAAAGACG
AATTTATTACATTATGTAACGTCTGTCAATCAGGCGTCAACTATCGCTTTATCCCGACAACCAAACATTTTATTATAATG
AAATTTTTAAATAAGTTATCAAATTGTTAAATTAATCTTTAATCACGCAGGACACAAAATGATGCGTGGGTTACCGATTC
AAAAAGAAACTTGAGCCGAACTCTAACGATATATTTGATTTAAAAAGATATAACGGC

>p.polycephalum_GT_AG_Contig23.12_318,388_2
PWM=homo_gtag_U12;5'ss_score=1.8252;bsite_score=0.6039
TGATAGTGGTGTATCCTTATAGTCTTAATTTTGTTCAGTTCCTTTTCTTTTACACCTTCAGATCACACTAG

>p.polycephalum_AT_AC_Contig4714.2_631,757_1
PWM=homo_atac_U12;5'ss_score=2.6813;bsite_score=1.5987
CAACGAGATAATATCCCCCCTCCCCTTTCATATTCCTCTTTTCCTTCGTCCCTTTCCCTTTCCCCTTCCCTTTCTTTCCT
TTTCCTCCCTTATAACAAGTAAATTGACTAAGTTTACGATTCAAGTG

>p.polycephalum_AT_AC_Contig1693.3_1769,2040_2
PWM=mus_atac_U12;5'ss_score=2.2972;bsite_score=0.7371
TTATGATAGGATATCCAAAATAAAATTTTGCTTTTTCTAAATATTATTTATTTTTTTAGTATTCGGTACTTTCTCGAAC
CCCCCCCCCCCCCCCCCCCAGCAAAATGATAAGAATAATTCAAAGCACTTAGTAACTTGGTTCTGTGGTATTTATTTCAA
TTTGAAAAACTTGCCATTTTGAAAATTGGTATCAACACTTTTTTCTTAAAACGTCCATCTCCTTTTTGATTAAACACACA
TTATCATTTGATTTTTTTGTACGTATATCATT

>p.polycephalum_GT_AG_Contig1013.10_2668,2740_1
PWM=droso_gtag_U12;5'ss_score=1.5070;bsite_score=0.7409
AAGATAAAGCGTATCATTTTATATCCCCTCCTCTTTAGTTAGTTATACTTTGATTTTGATAAGGGCAAATCTC

>p.polycephalum_AT_AG_Contig3341.5_1625,1703_1
PWM=homo_atac_U12;5'ss_score=3.0368;bsite_score=1.4075
GCGAGATTTAATATCCTAATTACCCTCCACCTTCCTCCTTTCTCGTAGCGGATTTTTTGACCAAGTCAGATATTTTATT

>p.polycephalum_AT_AT_Contig10882.2_908,1073_2
PWM=mus_atac_U12;5'ss_score=2.6387;bsite_score=0.6051
ATTTCAATCAATATCCAAATGATGAATAATAGTTCTCATTTCAGTGTTTAGTAACATCATTCACGTGATCCTACGCTTCT
TCTATTTGCGCTTCAGCATGAATGAAAATTTCTTTACTTCAATATCTATCTTGCGGGAGCTTTGATTATTACAAATACAT
TGTGCT

>p.polycephalum_GT_AG_Contig3900.8_125,516_2
PWM=homo_gtag_U12;5'ss_score=1.7702;bsite_score=2.1532
CGCCTTACGTGTATCCTTGCCAGCCCTCTTTACTTCGTTTCTTCTTTACTACTTGGTATCTGTAATTTTTCATTCTTTCT
TCCTTTCCTCTTTTCTTTCATGATGTACCTGCCTTAATCCCGTGGGTATGAAAGGAAGTGACAATGCATTATTAAAGTAT
TCCTTTGGCTTGTTTTTGTTGCAATTATTTCTTTTTTTTGGGTTGTTCTTTATTATCTTTATAATAGTTCGCCTTCCTCT
CCTGATAACTCTTTAACCTACAAAAAGAGAGACATGGATGCTTCTTAACCCTTTATCTGCTCTCTTGCATCTTTCTCTTC
CACCCCTCGATTCCTCTTACTCTCCTATCCTCTTCCTCCCAACTTTCCTTGAGGCAGCCAAGATATACCCCT

>p.polycephalum_AT_AA_Contig2411.4_1619,1760_1
PWM=mus_atac_U12;5'ss_score=2.2834;bsite_score=0.5213
TCCTTTGGTAATATTTTTATTATTATTATTATTATTTGTTGATTTTTTTTAATAAAAATTTTCTTTATGTAAATTTTCAT
TTCCCTTTTATGTTTTTGTTTTTATGTGTTGGCTCACTTATTTTGTAGGTAAAATGGTGTCC

>p.polycephalum_AT_AT_Contig8550.1_1182,1391_1
PWM=mus_atac_U12;5'ss_score=2.6187;bsite_score=1.2076
TCAACAAAGAATATCCAGAATATAACATTATAAACAATTTCCTGATATCCAATGCACAGCCTTATCTATACAAGACCTCT
TCCCCCCCCCCCCCCCCCCCCCACCGTATATTGCACACATTGTATTGGCACAATGTTTGCACAAGCAAATATATTAACCA
TTCACACTAGATTGTCCTAAGAACATTTGATGATATGTATGAAGAAAAGG

>p.polycephalum_GT_AG_Contig345.2_296,486_2
PWM=mus_gtag_U12;5'ss_score=1.5791;bsite_score=1.3835
TGGAGAAACAGTATCCTACTTTCACCTTCCTACGCATAGATCTCGTATGTTACGTAAGAGTTTTAGTTTATTACTTAAAT
AACAAAATCCTTCAAATACACAGAAAAACGCGTTTGATATCAAACTAAATAATGAAAGTTTATTCATAGCACCCTGATAT
ATTACTATTGTGACTATGAAGATATTTAAAG

>p.polycephalum_AT_AA_Contig915.10_61,419_2
PWM=homo_atac_U12;5'ss_score=2.6434;bsite_score=0.8795
TATACATCAAATATCCCCTTTTTCTCTATCTCCCCCTCCTTTTTTCTCTATCTTCCCTCCTTTTTCCTATCTCCCCCCCC
CCCCCCCCCTTTTTTTTTTGGACGTGAACCCCCTGGTACCTGTAACACTGAGAGATACAACTAACTCGACAAATTATACT
CAAATATCCTCTATTCTCTCTTTTTTCCCTATCATCTCCTTTTTCTAATATCTTAAGAGTGCAATATAGTCTATATTTTC
CCTATGAATTATTATTAAATTCCCTATTCTATCTCCCATATCTCCCCTTTCATTTTCCCTACCTATCTCCCTCCTATGCC
CTTTTAAATACCGTTTGATTTTTTGGTAAATATTTATTC

>p.polycephalum_AT_AC_Contig21377.1_841,1018_1
PWM=homo_atac_U12;5'ss_score=2.8741;bsite_score=1.6467
CACGTAGTTGATATCCTCAATCCGTCTGCATACTCCTACCTCCTATTCTAGTTCTCCTTTGTCTCCGTTCTCCTTCCTCG
CTTTCTCCCTCCATCCTCTTCTCCCTTCTCTTTCCCCTCCCACTTTTCCCCTTCCTCCTTATTGCAAAGCCATTCTGACT
CTGCGCACAAGTACCACC

>p.polycephalum_GT_AG_Contig1359.3_3334,3456_1
PWM=homo_gtag_U12;5'ss_score=1.6153;bsite_score=0.8990
TAGGGATTGCGTATCTTTTTTTTTTTTTTTTTTTTTTTTTTTTTGGTTATGAATTTTTATTTATTTTATTTTATTTGATTTAC
CCTGCCAAATCTTTATTTTGACAATTTACGTAGATATAGGGGA

>p.polycephalum_AT_AC_Contig1726.4_3336,3496_2
PWM=homo_atac_U12;5'ss_score=1.8356;bsite_score=1.1880
CTCATGCGAGATATCAGGCTATCTGTTTTCTTTTTTCTTTTTTTTTTCTTTGTTAGCGTTTTTTTTCAATCCAAACGGAA
AATATATACGTCAAAATTGCGATTTTTTTTAACAAAGACAGCTTTATCCTAATTTTATTGACCAAAAATACACGAGAGAT
T

>p.polycephalum_AT_AC_Contig3281.6_130,314_2
PWM=homo_atac_U12;5'ss_score=3.1563;bsite_score=1.1397
ACCCGATGCGATATCCTGTCTTTATTGTATTTTTCATTGTAATTTTCATTGCTTGGATTTTTTTATTATTATTGTTTTGC
ATTGTTTTATAATTATTATTAACTTTGCGCCGCTTTTTTCATTGTATTTTGGGTTATTTCTTAGTTGTATTATTTTGTAT
CTTTGATTTATGAACTTATCTCAAT

>p.polycephalum_GT_AG_Contig510.13_1093,1185_2
PWM=homo_gtag_U12;5'ss_score=1.5645;bsite_score=1.3279
CTAGTTCTTTGTATCATTTATTCTAGAGATCATCAATTAACTGATAATATTTTAGGATCGAAGTTCTTTAACTTTGTCGA
AAGGTTCTTCGGA

>p.polycephalum_AT_AT_Contig3775.5_2041,2159_1
PWM=mus_atac_U12;5'ss_score=2.5155;bsite_score=1.4218
TTAAGAAAAGATATCCACTTCTCTTCTATTTTATTTTATATATTTTTTTCCAATGAGCCTTGCACATTTCATTCCGCAGT
AAAATAATATTTTAGTTGATTGTGAATATATACCCAGAC

>p.polycephalum_GT_AG_Contig5735.1_2640,2998_2
PWM=mus_gtag_U12;5'ss_score=1.5237;bsite_score=0.5400
CACTTAAAAAGTATCCTTAAATAAACTTTGTACAAAACATAGCTACCCACTTTTGTTTCTTTTTATATCAAAATGTTCAT
CACAATGATCATGGACGACTCAATTTCTTTTTTTTATGGTCAATATTTGTCATCGTTACTATATCATCTTATTTGGTCGC
ATCAACAAATAATCACTAGCTTATAGTAGTTTAGTTCTAATTTAAACTACATCTCCACAAGTAATATTATTGTGAATACG
CAAAATTTCACATCACCAACTCGCCTCGCGCATCTATGTGTACATCGCTTGCCATATGCCACACGTTCACCATATATATG
CAAATTAAACATACTTTGATCAAGGAAAGCGACCTCAAC

>p.polycephalum_AT_AT_Contig1248.6_1395,1550_2
PWM=homo_atac_U12;5'ss_score=2.9370;bsite_score=1.8958
GAATTATTTGATATCTTTTCTTATCATGTGGTAGAACGAGACTATGTTTTTGCACAAAAAAATGCAAATTTAGTCCAAAA
AGTGTTTGAACAAAAAAAAAGTCCTATTTCCCTTAATGTATAAAATTACACTTTGATTTTCAACATAAACCTTGAA

>p.polycephalum_AT_AC_Contig5831.2_713,810_2
PWM=homo_atac_U12;5'ss_score=2.9739;bsite_score=0.7377
ACATTGATCGATATCCTACTACTTCACCGTCTATTCCCGCTATTCGTCTCACATATACAGTTACCATATTTTCTTTAATT
CAATCCACCTATTTGTCG

>p.polycephalum_GT_AG_Contig635.8_1521,1587_2
PWM=mus_gtag_U12;5'ss_score=1.4923;bsite_score=1.3367
GCATACAACAGTATCCATCCTTATCTCCCTACCATTGAAAAAGCTTAACTACCGAAGATATGATCGA

>p.polycephalum_AT_AA_Contig915.10_1572,1719_2
PWM=homo_atac_U12;5'ss_score=3.1827;bsite_score=0.6711
GCAGACCTCAATATCCTGATCTTCTTTATTTTCTTGTTAAACAAGAATTGTCTTATTTTTTTGTAATAATATGGATATGT
ATTACCTACGCTCATATGGAAACATACCTCATTTAGTTATTTTATTGACACTCCGAAAATATGTTTAG

>p.polycephalum_GT_AG_Contig1006.8_373,619_2
PWM=homo_gtag_U12;5'ss_score=1.4842;bsite_score=1.9363
CAAGAATTATGTATCTTTTTTATTTTATCTTGAATTATTTTTTTTATTTTTTTTTATATAATTATATAAATTATAAAACG
AAAAATATTAAAATGCCGTTTTTAATTGGACGACTCTAGAAAGATTTTTTTTCACGAAAAACAGACAGTAAAAAATTGTA
GTTTTGCACTCCTGGTGTTCCCCTTTCCCCCCCCCCCCCCCCGTTTACAATTTTCCCCCCGCTTTGACAAAAATCAGCCT
GCTCTCG

>p.polycephalum_GT_AG_Contig1968.6_752,824_2
PWM=homo_gtag_U12;5'ss_score=1.5999;bsite_score=1.1610
AATTGGATTTGTATCATTTTAATTGTGACGGACTCAAATCTACTAAACCACTTGACTAGAAAGATATACCGAT

>p.polycephalum_GT_AG_Contig1931.3_1767,2198_1
PWM=homo_gtag_U12;5'ss_score=1.5290;bsite_score=2.1590
AACAGCTCTTGTATCCTCACCCCAATACCCCATTTCTTCTTTTCGCATGCTCTTTTTTTGTTGATTATACTGTTAGACTC

AAATCTTGATTAGATAAGGGCTACACCCTACACTTTTACCCCACTTTTTTCCTCATTGTGTTCTTCAACCTAAAATAGCT
CTATTAGTGCCCACCCTCCCTTACTGCGGTGGCTGTTTCCCTCCTTTTTCTTTTTAAGTCAATTTTTGTAGATTGCCGCT
TTGACCCAAAAATAGAGCCTCCTGGAGACAAGGAGCATGGGTGACGGCTACAATCTACACTTTAGCGACCTCTTAATAGT
GCCTACCCTCTTCTCCATAAATGCGGTGGCTGTTTCAAATCTTTTTCACTTCCCTTTCTTTTAAGGAAATTTTTGTAGAT
TGCTGCTTTGACCCAAAAATAGAGCATCATGG

>p.polycephalum_AT_AG_Contig3918.5_2250,2362_2
PWM=homo_atac_U12;5'ss_score=3.2253;bsite_score=1.0171
AAACTAAGAGATATCCTTTCCCTCCAATTTCATGTCTTATATCTCAAAATGATATATGATCTCATCATTCTCTTTTAACT
TTACCAATCTTTTACGTAATCAGATAAGGGCGT

>p.polycephalum_AT_AC_Contig5903.1_1474,1541_2
PWM=homo_atac_U12;5'ss_score=3.1853;bsite_score=1.1139
AAAAGAAAAAATATCCTTACTTCACTAAACTTTGACATTTTAATTTGATTCACTCCACCAATCTTGCT

>p.polycephalum_GT_AG_Contig20382.1_443,649_1
PWM=homo_gtag_U12;5'ss_score=1.9516;bsite_score=1.4059
ATGCTGATGTGTATCCTTCTTTTTACAGCAACACTTAACCTAAATCTTTCGATTCGGAGTTCGATTTTGTGAGGACTGTT
GTGTGCAGGTGTAGTGTGTGGTACGTGCACGGCGGAAGAGTAGTGGGGAAATGTCCACAAATTACTCTTCCCAATTGTAT
ATTCAATGTTTTGAATACATTCCTTGAAAAGTCATAGAGCAATCAAA

>p.polycephalum_AT_AC_Contig2178.9_438,615_2
PWM=homo_atac_U12;5'ss_score=3.3099;bsite_score=0.6254
CTTCGGTAACATATCCTTATTGCATTTATTTTTATTTGTGGCTTATTTTTATTTAATACATTCTCCACAATACACGTTCC
CCAAACACGTTCCCCAATACACTTAACATTCTTTCACTACGCTCGTAGTTTATTTTTCTTTTTTTATTATTTCTTTAATT
ATACATACATGTGCCGTG

>p.polycephalum_GT_AG_Contig91.2_15347,15488_1
PWM=homo_gtag_U12;5'ss_score=1.7345;bsite_score=0.5868
CGCCATCCTAGTATCCTTCCTTCTTCTGTTTTTAATATCTCCCCTCTTTTCCTCTCCCCACACCTTTTCCCCAACCCTTT
CTCCCCCTCCCCCCGTCTCATCTCTCTTTTAATTCTCTTTTGACATACAAAGATATATTGTT

>p.polycephalum_GT_AG_Contig6283.2_2974,3144_1
PWM=mus_gtag_U12;5'ss_score=1.5800;bsite_score=1.3101
ATTTCCCGCAGTATCCCTTTCCTCTTCTCCTCCCCCCCCCCCATTCCTCCCTTTCTGTCTCCCTCTATCCCCCCCCCCCA
CTTCCCATTTATGTTCTTTTATGCCCTTCCTTTCCGCCCTGCTATCCCTCCTTGTACCCTCATTCCGTTGAAACCGTAAA
GATATGTGGAT

>p.polycephalum_GT_AG_Contig9489.2_313,396_2
PWM=homo_gtag_U12;5'ss_score=1.6667;bsite_score=0.7116
TTGTACACGCGTATCCTCTAAACCAAGCACAAAAGCTCACCTCGTTAATTTTAAAGCGGAATTGACTTCAGAAGATTCTG
GGAA

>p.polycephalum_AT_AA_Contig2981.4_1589,1833_2
PWM=homo_atac_U12;5'ss_score=3.1916;bsite_score=0.9967
CTGTGGCTCGATATCCTCTTTATTTTTTTTGTTTTTTTATTTTTATTTTTTTGTTTGTTTTCTTTTTTCTTTTTATTTTT
TTTTTATTATTTTCTTTTTATTTTTTATTTCCCATGCCACACTTTATTTCATCCACGCTTCTCCTCAAGGAGCGTGGGCT
AGATATCCTCGGTTTTATTGTTTCTTTTAGTGAGCTTTCTTTTATTGTACCTCATTACTCTTTGACACAATTAAAATATT
TTGAG

>p.polycephalum_GT_AG_Contig3762.4_2592,2840_2
PWM=homo_gtag_U12;5'ss_score=1.4828;bsite_score=1.7741
TTCCACTTCGGTATCCTTACAATAGAGAATTGCATAGGAGCGTACTTTTTTCCCCTTTGGTCCACTTATATTGGATTTTG
AGTATTCTTACTACTTCAATCCACTCGCGCTATGTATTTCCATTTTCCCTTCGAAATTTCAACAATTTAAACAAATAGCG
CGATAGTTTCAGAGAGATATTTCGGAAAAAATCAAAAGAATCAATATAGTGTCATACACGCAAGCTTGACCAGAGGGAGA
TATGGAGCT

>p.polycephalum_AT_AG_Contig1532.10_1249,1477_2
PWM=mus_atac_U12;5'ss_score=2.1892;bsite_score=1.2339
AAAGAAAAAGATATCCAAGTACATTTTTAATGAGTTTTGCAAATTTTTGTGCTCAGGTTAGGAGAGGAGGGAGGGAGGAA
AGGGGGGGGGGGGAGAGAAGGGGAAAAGGGGGAGTAAGAAAAGGGAGCCATGAAACCCAGTTTTCTTATGGGATGTCAGC
ACGGTTATTAAAATTTATTTTGTTTGGATCCTTATTTGAATTTATTTGAAAAAATGCAGATATTAGAGC

>p.polycephalum_AT_AG_Contig3961.2_2452,2695_1
PWM=mus_atac_U12;5'ss_score=2.7751;bsite_score=0.7050
AAAGGAAAAGATATCCATTTCTTAATAAGGAAAGAAAAGGTATAGGGGCGTAATACAATCTCCTCCTGTCGGCTAAATCA
AAACCATATTCCCACATGGCTCCCTTAATGCTTCTCAAAAAGGGCGCAAGGATCTGCCTCCTTCCCTCCTCTTCCCTTTT
TTCTTTTCTCATTTTCTCCTTTCTCTCTCTCCCCTCCACACTCCCCCTCCCCCCCTTTTTTTGACCTTTAGCAGACCAAA
AAGT

>p.polycephalum_AT_AT_Contig8641.2_1387,1485_1
PWM=homo_atac_U12;5'ss_score=3.1728;bsite_score=0.5856
GTTTGTTTTGATATCCTGTTTACAAGGAAATGGACATTAATAAGAGGGAAAAAATCCCAATCTTTGCGTTCTTTTTGACA
ACATATTATATATAACGGG

>p.polycephalum_GT_AG_Contig9514.1_1657,2007_1
PWM=homo_gtag_U12;5'ss_score=1.7155;bsite_score=0.6139
GGTAGCTAAAGTATCCTTTCGTTTCGACTACCCGTAAATAGGCGCGTTTGTCGGTTTCTTGTCGCTAGTTCGTTTTATCT
GTATGTTCATCTCATCTCGCAATATAACGATGTACCACGTGATATAGTTTTCGTTTTGGTATGGCATGTAGTTACTACGC
AACTCTTTACCCAAAGTACAAGTGGAAGAAAATTGGGGGGGGGGGGGGGGTGTTGGGCTTTTATTTTTGCTTTTAATTTTT
CTCTTGTTTATGTTTTGTGGTTCTGCATTTCTTTCTCTTTATAGTCCATAGTATTATTATCAACATTTTGCACTACTCGA
ATTTGCTTTGATCACTAAAAGATATGCCAAC

>p.polycephalum_GT_AG_Contig3363.2_4780,4860_1
PWM=mus_gtag_U12;5'ss_score=1.5866;bsite_score=2.8794
ATAAAACACTGTATCCTAAAAAAACAATGAATTAATTTGTCTGTTTCCCTAACATTTTGACCATTACAAAGGTATGGAAG
A

>p.polycephalum_GT_AG_Contig1657.8_842,915_2
PWM=homo_gtag_U12;5'ss_score=1.8349;bsite_score=1.4119
GTTACAAGTTGTATCCTTATTTTATTAATTGTGAACTTGTGCTTCATCTCTTTGACTAATATAGTGGGATACAG

>p.polycephalum_AT_AG_Contig2569.2_8106,8605_1
PWM=mus_atac_U12;5'ss_score=2.6157;bsite_score=0.9226
CATCAAGAGCATATCCAAAAAAAGGCAATGCCAACAAAGAAAACACCCACTCCCATTTTCAGTAAAGTTTCCAATAAGAG
GAGAGAGTAACAGCAATAACAATAATTATTTTAAGCAAATCATTTGTCTTAAAAAGGAAATTTCCTTTTTAGAAAACAGA
TAAAAATATATAAAAAGTATTTTTTAGTTGTGGGTGTTGAAAGATCTTCAATCACAAGCTTTGATACTGAAGCAGGAACA
GGGGAGTGGTCTGTTATATTGTTAGTGAGGCTTTCAGTTTTTGCAAAATTCAAAAGCTAATTAGTCAGCAATAATTGACT
TGCTAGGGCAGATAAATAATATTCACTAAAAATATTTTATATTTTTATCTGTGACAGGCTTTCCTAAGTAAATTTTCTTT
TTAGGACAAACAGTTTTGCTTACAAAAAACCTAACTCCTCAAAATATTTGTACCAAACTCAATTTTCAATATTGGCTTTA
GTGTAAAAAGATAACATGTA

>p.polycephalum_AT_AG_Contig4148.3_4377,4475_2
PWM=homo_atac_U12;5'ss_score=2.0649;bsite_score=0.8014
CTGTGACAAAATATCAACCTCAGATCTTAATCTGCAGATACAATTCCATTTGAATACCTAAATAAAAATACAAATTGACA
TAGACAAAGATATATGCAT

>p.polycephalum_GT_AG_Contig801.11_62,335_2
PWM=mus_gtag_U12;5'ss_score=1.6787;bsite_score=0.8816
ATGAAATAGAGTATCCTTATTTTTTTTAATCGTTCTCTTCAGTCCATTCCTTCCTTCCCTCCTTCGTTCTTTTCATTTGC
ACCCAGCACATAAGACAAATACGCTAATTGGAAAAAATGAGGCTTTTAATTTTATATTTACTCTTTGTCCATCGTGTATA
GTACGTTTTTGGGCATTTCACAAAGCGCTTTTTTTAAATATATAAAAAGATACTTCTCCCATCTGATCTCCTCTTGCCTT
AGAACACATTTTGACAACAATTAGGAGTGCAGAA

>p.polycephalum_GT_AG_Contig9145.2_382,504_2
PWM=homo_gtag_U12;5'ss_score=1.4805;bsite_score=1.3799
TGGCTTCTCTGTATCATTGAGTTAGCTCTTATCATTCCTAGACTCACTTTCTCCTCTCCTACCACTATCTTCTCACACTC
TCCCTACTCTATTGACTTTGATAATGAGATTAGCAATCATATA

>p.polycephalum_AT_AC_Contig703.7_769,900_2
PWM=homo_atac_U12;5'ss_score=3.5083;bsite_score=0.6851
CCTCTCATTCATATCCTTATATAACTGATTTTTATGTTACATGTCTCGTTAAAAGATTAGTACGTGCTCTAAGTACCTTG
CAATTTAGTTTACTGAACTAAACTGTATTTGATTTACAATACACCGAAAAGA

>p.polycephalum_AT_AC_Contig1659.6_4075,4163_1
PWM=mus_atac_U12;5'ss_score=3.0091;bsite_score=0.8191
ACAAGTGCTAATATCCTCAAATGCAAAATCTTTCGCCTTTCATTCTATGCCCGTGAACATACTATGACTTGTTGCTTACG
TGCCCCACA

>p.polycephalum_AT_AA_Contig1112.8_286,451_2
PWM=homo_atac_U12;5'ss_score=3.0234;bsite_score=0.8175
CAAATTGTCCATATCTTTGGTACTCATATTTCTTTTTTTGTATTTTTAGAGGTAACATAAGCTAGTGAGTTATATCCCTT
AAACAAAAAGCCTAAATGTCTTAAAAATTGAATCTTCGAACCACTCGAGGCTATTCATATACTTGACCCTTGGTAAATAT
ACCTCC

>p.polycephalum_AT_AG_Contig527.1_2292,13922_1
PWM=homo_atac_U12;5'ss_score=3.6189;bsite_score=0.9047
ATGCTACAGCATATCCTTTTTCTTTTCTTGTTCTTTGCTTTCTTTTCTTTTTCTTTGCTTTCTTTTCTTGTTCTTTTTCT
TTTCTTTTCTTTTCTTTTCTTTTCTTTTCTTTTCTTTTCTTTTCTTTTCTTTTCTTTTCTTTTCTTTTCTTTTCTTTTCT
TTTCTTTTCTTTTCTTTTCTTTTCTTTTCTTTTCTTTTCTTTTTCTGTCTTTTCTTTCTTTTTCTTTTTCTTTTCTTTTT
CTTTTTCTTTTTCTTTTTCTTTTTACCACACAATTCATAATTATATAACGACAACATAGGGCAATGTACAAGCTTTAATT
AATTGGTGTACATATTTACCAGGTCCCTGAGAGCTACAACAAGCTCCTGGCCCATGTTGTGTGATACACATGATGAGCCA
CACCCATACAAGTTTTTATAAATGAAGAATCGCAGAACTGGATTTATCGACCCATTACACCCCATACAAGTGCATACATA
CGCAGGGAAGTTCAGTCAAAACAATCTGGCAGCTTGCCCTCGTTCGTAAATTGATCTTCGAAACCAGTCAGAGTAACCAA
AGAACTAGGAGGTCCTATTTATAAAAAAACAAAGGTTGTGGCTCATCATGAAAGAACGAAAAAATGTTCCCTGTGTGCTT
TTGTGTGTCTGTGCGTGTGTTCTCTCCTTTTCCTAATGTGTCTTCCTTTCTTTTTTCCTCCCAATTTGAGGGTGGGAAAA
CACCCCAAACCGTGCCCATCTCTACTCCATCACCACTTCCCAAAGTCTATGTCCATGCAATGCTTTGTCTCTCTTTTGGT
GCTATCCCCTCCACCTGATTTGCAGTTTGCCAGCCAGCTAGTAGGTATCCATCCAACACTCATACATCCCATAACTTGCT
TCCATAACCACTACATCCCATTCCACGATTTGTTCCATGTCCCAGTATGCACTCAGATTCAGAGAACAAGAGAACCAACA
GTTATGATGTCCAAAGTTGATGTCATTTGAATCATTACAATTCCCACAGAAACTAGAATGTCCAATTTTGCAATCATATT
CCAGAATCTATACATCTTCCTAGTATCTCCGTTGCCCACCCGCAATGTGTAAACCTTGGGCTGGGTTCCACTAGTGCGTT
GTTTACCACACAATTCATAATTATATAACGACAACATAGAGCAATGTGCAAGCTTTAATCAATCAGTGACATATTTACCA
GGTCCCTGAGAGCTACAACAAGCTCCTGGCCCATGTCGTGTGATACAAAAGAACGAAAAAATGTCCCCTGTGTGCTTTTG
TGTGTCTGTGTGTGTGTTCTCTCCTTTTCCTAATGTGTCTTCCTTTCTCTTTTTCTTTCTCTTTCTCTTTTTCTTTTTCT
TTCTCTTTCTCTTTTTCTTTTTCTTTTTCTTTTTCTTTTTCTTTTTCTTTTTCTTTTTCTTTTTCTTTTTCTTTTTCTTT
TTCTTTTTCTTTTTCTTTTTCTTTTTCTTTTTCTTTTTTTCTTTTCTTTTCTTTTTCTTTTTTTCTTTTCTTTTCTTTTT
CCTTTCTTTTTCTTTGCTTTCTTTTTCTTTGCTTTCTTTTTCTTTGCTTTCTTTTTCTTTGCTTTCTTTTTCTTTGCTTT
CTTTTTCTTTGCTTTCTTTTTCTTTTCTTTTCTTTTTCTTTTTCTTTTTCTTTTTCTTTTTCTTTTTTTTAATGCACTGTGGTGCA
GGGTTTAAATTGGGTGGAGTGAATTTTTCACTTCCTTTGTGCACTTCTCCTCAACACCTATCTCTTGCACCCTTCCCTAC
CATCCCACCTTGGACATGGCTCTGGTGAGCTCACTATTTTGTTCTCAGTTTTTAAGAATAATTCAAGAAGTACAATTTAA
ACTTTTTCTTTTTCTTTTTCTTTTTTTTCTTTTTCTTTTTCTTTTTCTTTTCTTTTTTTCTCTTTTTTCTTTATTTTTCT
TTTCTTTTTTCTTTTCTTTTTTCTTTTCTTTTTTCTTTCTTTGTGTTTTGTTTTGTTTTGTTTTGTTTTGTTTTGTTTTG
TTTTGTTTTGTTTTGTTTTGTTTTGTTTTGTTTTGTTTTGTTTTGTTTTGTTTTGGTTGTTTTGTTTTGGTTGTTTTGTT
TTGTTTTGTTTTTGTTTTTGTTTTGTTTTGTTTTGTTTTGGTTTGGTTTGTTTTGTTTTGTTTTGTTTTGTTTTGTTTTG
TTTTGTTTTGTTTTGTTTTGTTTTGTTTTGTTTGTTAGTGAGAGATTGGCTAAGTAACTATAGCAGGTAGAGGACCTTAA
GCAAAATCATGTGGTTTTTTCACCCATACAGGTGATTTCTCCACTTGTGCATTAGGGTAAATAAAATTGTTGCAGTACTA
TCTTGGCAAATGATGATTATGCAAAAGTATGGGGGAAGCACCTAGAAGTGGCAGCAACCCATATCTGAAGATCTAGGCAG
GTCTTGCCTACAGTGCAACCCCTGGGATGCTCTACAAACCAATTGAGTAACAGGGAATGGACTGAAAAGCCTAAGCATTG
TTTTGTCCCAGCTGCAAGTTAGCATCCATCTACTGAAGCACAATCAAGAACCTAAAGTACCAGCTCTCTACAACTACAAG
AAAGAACAAGCAGAGGGTAAGAAGAGGTGGAGTGAACAGTAGCACTAAATCAACACAAAAGGAATAGCTACACATGGTGA
CAATGGCATCTTAAAGCTGATAGCACAGGTAGTACAAACCTCAACTCTCTCTACAATCCAAAAGCAACTCAAGGCATTAC

CTTCATCAAAGACTAAATAGCACCCCTAAAGACCCAGCAGGTATCTTCTGCCTTGTGAATGACTGGAAAACAAGTGTAGA
TCTTGATCGAGACACATACTCTTTCCAGCTCACATCACACAAACCTCTCTCCACCCAGACATCCTCATCTGGTCAGTTAA
GGTGAAACATATTCTCTCTTGAGACACATACTCTTCCCAGCAAACCTCTCTGCCCAGACCCTCATCTGGTCAGATAAGGT
AAAGCACATTCTCTTCCTTGAGCTCACAGTTTCTCAATAGGAAAACACACCAGGGGCACAAATATGAAGCTCAAAAGGTA
CCATGATCTAGAGCAAGCCTGTAAGTGTATCAAACATTCTCCCTTACTTTGGAAGTAGGGAGCAGGGGGTGGGTAGCACC
ATTAATTGGGATGTGTATGAGGAAACTGGGTATTTGCAGGGCCAAATCAAATCCCTTCTCCACCACTCTCAAGTACAGTC
CTGTGTGTGAGATATCTAATCTATGCTATCAAGAAAACCTCTCCTGGAAACCATGAAAGTGGAAAGTCTCTCTCCTTGTG
GGGACCAAGTAACAAATTGGAGTCTTGGACCAATGTATACAAGGTTGGGCAGCAGTTTGTTTGTTTGTTTGTTTGTCAGT
AAGAGACTAACTCATGGACCCTCCTGGCACTCTTCACACCTAGGTGACCACTGGGGGGGGGGGGGGGGGGGGGGATGCAT
GAGGCACCAAGCACCTCACCTGTTTTCTGCAGTACTAGTAGCATGTACACATGCATAGCAATAGCACATTGCTGATTTGT
GGCTAATAGTACATATTGTATACTTCTGTACTTAATATATTTTGTTTGTTTGTTTGTTTGTTTGTTTGTTTGTTTGTTTG
TTTGTTTGTTTGTTTGTTTGTTTGTTTGTTTGTTTGTTTGTTTGTTTGTTTGTTTGTTTGTTTGTTTGTTTGTTTGTTTG
TTTGTTTGTTTGTTTGTTTGTTTGTTTGTTTGTTTGTTTGTTTGTTTGTTTGTTTGTTTGTTTGTTTGTTTGTTTGTTTG
TTTGTATAGTGTGTAAAAAGAGGCCCAGTCCAGCTCCCCACGCCTAGGCAACACTGGACTGGTGGGACGCGTGGGGCCGC
GAGCACCCATTTGCTTTCTTTTCCCTTCACATTATGCAACCAAGTGGAAATATATCATTCGACACCATATGACCGTTGTG
TGCACTGCTGTGGAAAGCAGCTGATATCGTGGTGTGAGTGTGCTAACTCTTCTTTTCTTCTTGACTAGTACTGGCAGAAA
ACAGGTAGAGGTGGGAGGTGCTCAGTGCCTCGCACATCCTCCCCCTTCCCAGCAGTTGCCTAGGTGTGAGGAATGCCAGG
AAGGTCCTCTACCTGCTATAGAAAGTGTAGAAAGTACAGAAGCGTACAGATACATACTAGTAGCCACAAATCAGCAGCGT
GCTATTGCTGTGTACGTGTACATGCTACTAGTACTGGCAGAAAATAGGTAGAGGTGGGAGGTGCTCAGTGCCTCAGGTGT
CCTCCCCCTTCCCAGTGGTCGCCTAGGCATGAGGAATGCCAGGAAGGTCCTCTACCTGCTATAGTTAATGAGCCAGTCTC
TCACTAACAAACAAACAAACAAACTGCTGCCCCACCTTGCAGAAGTTGGTCCAAGACTCCATTTCATTACTTGGTCCCTG
TTTCAAAGTGTCCCCAAGCCAGGGGGTTGTTGAGAGTTAAGAAGATAATGCAGGTTGTTCACCCACAAGAAGAGAGACTT
TCCACTCCCATAGTTTCCAGGAGTGGTTTTCTCGGTTGACATAGATTAGATAGCTCATACGCAGGGCTGTATTTGAGAGA
CTGCAAATAAGGGATTTGATTTGGTCCCTGGGAATACCCAGTTTCCTCATACATGTCCCAACTGATGGTGATACCCACCC
CCTGCTCCCTACTTCCAAAGTAAGGGAGTGTGTTTGGTACCCATTTGCCTTACATGCTTGCTCTAGGTTGTGGTACCTTT
TGAGATTTTGTATTTGTGCCCCAGCTGTGTTTTCCTCAAGTGGAACTGTGCTCAAGGAAGAGAATATATTTCACCTTATC
TGACCAGATGAGGATGTCTAGGTGGAGAGAGATTTGTGTGATGTGAGCTAGGAAAGAGTATGTGTCTCAATCAAGATCTA
CACTCATTTTCCAGTCATTTGTGAGGGGAGGATACCCATTTGGCCTTTAGGAGTGCTATGTGGTGCTTTCTCTCTTGCTT
TGATAAAGGTAATGCATTGGGTTGCTGTTGGATTGTAGAGAGTTGAAATTTGAGTCTGCTATCTTTCCTATCAGTCTTAG
GCACTGTGTGTAGCAATTGTGGTGTAGTGTTGTAGAGAGCTTGCACTTTAGGGTCTTGACTGAGCTCAATAGGAGGAACT
TGCAAACTTGCAACTGGGACAACAATGCTTGACTTTTCAGTCCAATTCCCAAGAACTTGAAGGCGTACTTGAAACTAAAA
TTACCTCCATTGGTGAGCTGTGGATTAATGTGTGCTAGCTCTTATATTCAAGACCTCTACAAGGTGATTGTAACCCTTTT
CCACACTTGCCTTAGTTAGGCTCATACACTTGGATGGGTGGGCTGTTGTTTCAGTTTGTCAAAGAAAACCCCTGTGATAT
AGGAGGGTTTGCTTTCTTGCTAGTGCCATGTGACAAGGTGGTGTCATCAGCAAAGAAGAGGCATTTGACTTCCAGCTCTG
TCTTCTTACTATGCAACATTAAGGGGGGCCAGCCACTCTTCTAGCATGTTGTAGGTGATATTTAATAAGATGGTGGGGAG
TGTGCAATCCTGAAATAGGCCACTTGAAGTTAAAGTTGGAAGTAACTACTTATGTTATAAGAGGTCATAGAAGGAAAGAA
TAACCTCAGTATAGTGTGAGGGAAGTAATAACACTCAAGAGCATGCAATAAGAGAATGGCACTGTTCTATAGGATTCTTC
AGATCTAGAAAGGTGACAGTAATTGCTTTGACTCTAATGGGCATCTTGTAGAGCTGCCATTTTGGAAAGGGCTTTCTAAA
GTGAGGGGATGTATGGTGCCTGATTTTGACAAAGACTGACAGGGAGTGTCACCTGTTTCAAAGCATGGTTTCAGGAGCAG
TTTTCTTGGTTGACATAGATTCGATAGCTCATATGCAAGGCTGTATTTGAGAGATTGTGAAGAAGGGATTTGATTTGGTC
CCTGGGAATACCCAGTTTCCTCATCCATGTCCCAGCTACCCACCTCCTGCAATCAAACAAACAAACAATCAAACAAACGA
GGAAGAAGATTGAAAATTAGCAGAGTTGGAACACAAAACAAGCCAATTATCGCAGCACATGCACTCTATCCTCCCCTACA
CAATGCTTGTGGCAGCAACAAAGGAAGCAAAGGGGTGCTTGTAGCCACATGTGCCCCCACTTAATATCACCTATGGGCAT
GGGGAGTCAGGTGGGCTTCCCAAATTGCTACCACAAACAAACTGCCCCTCCTCACATAATGCAATCCAAGTCACTATTGA
TTCACTCGGTCCCTTGCAATCATCAGCCCAACAAATTATGCTATACATCCTCCATCAGTTTCCTCAGGACCTATGGTTGT
TGTGTTAAATCCTTTCTTAGCAACCACAATTTTGGTTGAAAGCTCACTCCAGCAGTGGTGAATGATGATGATATTGGCAA
CCTATTCCTCTACTCAGAGCTTTGTGTAATTCATTGTTGTTGAAGTACGTGAGGCTGTCTCTCAGAAGATTTTAGTTTAC
ACTATGGCCTAACTAGTCCTCATAGGTACAGGGGTTTTGTATTGATGAATGTGGGGTGAAATAGGTTATGTGCTCCCTCT
TACTGACAAACAAACTGCTGCCCCATCTCACAGAAGTTGATCCAAGACTCCATTGTTATTGGTCCCTATAAGATAATGCA
AGTTGTTCACTCACAAGAAGGAGACTTTCCACTCCTATGTGTACAGGAGCAGTTTTCTCAGTCAACGTAGATCAGATAGC
TCATACACTGGGCTGTATTTGAGAGATTGCAAAGAAGGGTCCCTGGGAATACCTAGTTTCCTCAGACACATTTCAACTGA
AGGTGCAGAATGTAGTCTAAACCTCCATGTTGCTGCTTGGTCCCTTGTAAATGAATGAAAATTGTCCACTCTATTCGTGA
CCCTATTCCAGTTCCCTAATCCCACAGGTTTCAGTGCGCGTGCCTGACAAAGCTTCATTGCAGGTTGTGAGGTCCTTTGA
CCTCCAGTCCGTATAGACAAGTCCATCCCAGGTACCTCCTCCCACGGTGTGGCTACAGTTGTGTTGCACTCTATAGAGTT
TGGAGCTCATGAGATTGAGAAGTTTCCAGGTTACTAAGGTAGAATGTTAGAGAATTTCTGGTTTGAGTTAGGCACTGAGA
CTGTGATGGTACTATCGACCCAGTATTGCCTGCCAGCAACTTATGCTGTGCTGAACTGGATCTCAGGTTGGTTGTCCTCT
GGAGAAAATTGCCAAGTAGAAGGTAGCTAAGGGCATAATGTGAAGAGAAAAGAAAACAAATAGGTGCTTGCAGCTCCATT

CACTAACAAACAAACAAACAAGCTGCTGCCCCACCTCGCAGAAGTTGGCCCAAGACTCCATTTCATCTTGGTCCCTGTTT
CAAAGTGTTCCCAAGCCAGAGGGTAGAGGGCTTGCACTTTAGGGTCTTGATTGTGCTTGAGCAGGTGGAACTTGCTAACT
TGTAGCTGGGACAAAACACTGCCAAGGCTTTTCAGCCCAAGTCCCTTTTGCTTAATTGGTTGGTAGAGCATCCCATGGGT
TGCACTGTAGGCAAAACCTACCCAGATTTTAGATATGGGTTTGCTGCCTCTTCAAGGCGCTTTCCCCATACTTTAGCATG
ATCGTAGATCATCATTTGCCACGATAATACTGCAACAATATAGTTGTTATAGATCCACAACTTCATGGCACCACTGAAAT
AGACCAATGCTATAGTGTACCCACTTGGAGCTAAAGTTAAAAGTAACTAGTAGGATAGAATGATTTTTAGTATAGAGTGA
GGGAAATGGTACCACTTCAGAGCATATGCAATGAGAGAGTGGTGCACTGTTCTATAGGCATTCTTTAGATTAGAAAGGTG
ACAGTGATTGCTCTTTGACTCATCTCATAGGGCTGAGACAAGGGTTTGTGTATGTTCTACACAGCCTGCCATTTTGGGGA
TAAACACTTGTGGGTGGTCCAAAACGGAAGTATAAACCTTGACACTGGCAGGGTTTGTGCTAGCGATCACTAGTGTCAAA
AAAATTAACAGCACTAGCACACTTGCCAGCGAGGGTTTATACTTTTTTGTACGGGAAATTTTTTCCCCCCTGTACAAAAA
AGTATAAACGTTCGCTAGCAATAGCACTAGCGACAGGTTTTTTTTTTACGCTAGCGTTCGCTAGCGACCGCTAGCCTAAC
CCCGCTAGCTCAAGGTTTATACTTCCGTTTTGGACCACCCACACTTTCTGAACTGAGATGTCAAGATAGCTGTTTTGCAG
GACATATTCAGTGGTTCTAGCTTGGAATAGGGTGAAGAAGAGCTTTCCTAGTGTGTTTCCAAGGGCAATAGGGCAGAATT
GGTCGGCCTTGCTGGTATCTTCAGTTTTGCTGATTAGGATGATATAAGCAACTTGTCCCCTTCAGAGACTACTTGTTCAT
CCCATAAGCATGTAAAGAAAGAGTGGATGTATCTTTGCAATATACAAGATAAGGAATGGCATTCCATTTTGGGCAGGAAT
TATTGGGCTTTTTGTGGAGCAAGTATTTAACATCACCTAATGTAGGTGGAGCAAGGTTGAATGGGCTTTTTGGAGGGGGT
GGGACTTTTTCAAGCTGTGGAGGTGTAGTATAGGACTTATTTCTGCCTACATCCTTATAAGTAGCTGAAAAGAAAGTGTC
AGTATGACTTTTATCAAACTGGGGGATCTGCTGCTCTGTGTTCCTAAACTTGAATATTGACCCAACCTGGATTCACAGAA
TTGCCATGGATCTCTTTTGAATTCTCTATGGGAAGTGGATGCTATCAGCTCCTTTTCTACTTGTTGCTTTTGTTTTCTCA
ACTTGTTGAGGCAGTGATCACTTTTCAAAATTTAGCTCTTGCTTCCTTTGGGCCCCTTATCTTTGGTTTGCTTGGCCTTC
TTCCACTTTTTGCAAATTTCAGCTTAGCTTGCAGAGCATGTCTAGTCTCTATGTCGGGTTTTATCTGCTTGTTGTCTCTC
TTTTTTTGTGTGTGCAGGGTTCACTGTTTCACATTTGATACTGATGAACTAGTGCACAATGCTAAGGAATGCATTTACAG
TGGCTGTGATGCCTTTGTAGTGCAACTCTTTGAGATTGAGTTGCTGATGTAGGACTGCTTCCAGTTCCTTATTAATTGAT
GACCACTGTATGTGATTGTTTGTCTTGGGGAGTTTGAGCTTGGGTTGTGGAGTGTACTTGTTGAAATGGTCTTGGTCTAG
GGTATGGTAGGGTGAGAATGGTTGATCTTGGTCAAAACAATTAGGTGAGGCTGTATCACTGGGACTGTGAGGGTTTATAC
TCAGCTCAGTATTGGCCTCCAGAGACTTAGTTTGTTGCTCTGGCATAAGAGAGTCTTTGCAAGATGAGCAGCTTCTCATG
TGGAAAATGACACGATGGTGAAACTTGTGTGAGTATACACAAACACAACTGTGGGTGCCAGTCTGTTTGTTGGTAGGGAC
AGGGGCATCCACATAACTGGTGTGTTGCTCTTGAGCCTAGAGCACTGAGGTTTCACTTTTTTATCCTAGGGCTTACTTGC
CTGTGGTAGCTTTTGGCGCCCAGCATCTGAGGTCATTGATCATGTCATAACCATAATGTGTTGTTGGTAATCCAATGTTG
AAGAAAGTGTAGAAAGTACAGAAGCATACAAATACATACTAGTAGCCACAAATCAGCAGTGTGCTATTGCTGTGCACGTG
TACATGCTACTAGTACAGGAAGACCACAGGTGACTGTGCACTCTGTCACACCAAAAACATCACACTCCTCCACATCCTCA
ATTGCTGCTGCTATTCACTGCACCACAATTGCTACACATGGTGACACAATGGCATCTTAAAGCTGATAGCACAGGTAATA
GAGTCAAACCTCAACTCTCTCTACAAACGTCTTGACATACTCTTTCCCAACACACATCCAAATCCTTTCCACTCAGACAT
TTTCATCTAGTCAGATAAGGTAAAGCACAGTCTCTTCCCTGAGCTCACAGTCCTACTTGAGGAAACCACATCAGGGGCAC
AAATATGAAAGCTCAAAAGGTACCATGACCTAGATCAAGCCTAGGCAAATGGATATTAAACACTCTTTTACTTCAAAGTA
GAGAGCAGGGGGTGGGTAGCACCATCAGCTGGGATGTGTATGAGGAATTGGGTATTCTTAGGGAAAATCAAATCCCTTCT
TTGCAATCCTCAAATACAGACCTATGGATGAGCTATATAAATTTGCAGATTAAGTTAGGAAGGGGGAGGATACGTGAGGC
ACCAAGCACCCCATATCTACCTGTTTTCTGCCAGTACTAGTAGCATGCATATGTGCACAGCAATAGCACACTGCTGATTT
GTGGTTACTAGTATGTATCTGTGTGCTTTTGTACTTTCTACACTTGTTTGTTTGCGATGTAATCCTAAGAAATATCTATA
GATGAAACCAGGGAAGAGATACATGGAGCTAACAAGCACTCCTCACCTCTTTGTGTTGATATCATGAACAGTTGTGTGAG
GATATCCACATATTGCATGCATTGTGATAAAAAGCAATTGATTTGCGTGTGTTTGATCATCTGTTGGCCACTATTTACTT
TTCTTGATAGGAAAGGTGCATAAGAATGGGGCTCCCATCACATTCACATGCTTTATTCCTCCTAGTCCTGTCAACTACCT
TTTGCACAATAACAACAACTACTATTACATCACCTAATCCATACACAAAACTAAGACTCTACATCATGCTGACTATTCCA
TCACATACCTGGCAAGTTGGATTCTGGGCTTCCTTTCTCTCCCTGGAATTTCCCAGAGTGCACTACCCTAGGCTGCAGCT
ACAACCACAGTGATAAATGGGAATCTTCTGGGAGTTGGTGCTCTTTGGAGGAAATTTGAGTTCTCAATATTGATACTCTG
CAATCAAAGTCCTTAAGTAGCTCTAGTTAGAGGCCCTCTTCTAAGGCAAATGCTCCTACACTGTGAGGATTTACACTACC
ATGGCCCCAGGAACCTTCATGAACTAATCTGCTAGAGTGGTTTAAACTAGTAGGCAGGCAGTAGTGACCCTACATGTGAC
CTAGGAGTTACACACTATGAGCTGCTATTTCCAATTTCCAGCTTCCATTAAGGGACCAAGAAGATTAATGGTGTCTTAGA
GCAGGTTATTTGTTTGTTTGTGATCCCCCATGTCTATAGACAAAACCAGGTGAGGAGACACATGGATGTACTCCTTGCCT
CTTTGTGCTGATATTGTGAACAGTTGTGTGAGAGGATATCATCCACATAATGTGTGCATTGTGATAAAAAGCAATTGATT
TGCATGTGGGTCATCTATTGGCCCCTATTCATCTTTATTGTTTGATTGTTTTTGTTTTGTTTGCTTGTTTGACCATTTGT
TTGACCATTTGTTTGTTTGTAATATGAGCAATCAAAAAGACATGGTGTGTACAACCAGCTTTATTAGAGTAGACCAGTCT
CTAAATGAGCTGTTTTCAAACAAATGTGTGACATGTGTAAACTAAATGAAACTAACTTGGTTCTACTGCAAAAATTACTT
GTAACTTGAAAAGTGGCTACCTTTTTTGATGTGGTGACACAACTGTTTATGTTTAAGTCCCTCCTGTGCCCCCAAATAAT
TACTATTTTACATCAAAATAGATACACAGGG

>p.polycephalum_GT_AG_Contig1135.9_1420,1534_2
PWM=droso_gtag_U12;5'ss_score=1.6390;bsite_score=0.8481
CTACTCTAAGGTATCCTTTGTGAACTTAATCTGTCGTTAAATTTTAATGTCTATTTAATATACTTTCTTCGTGTCGCTCT
TCCCCGCTCTAATATATTCTTGTAGTCTTCATGAT

>p.polycephalum_AT_AC_Contig4658.3_379,630_2
PWM=mus_atac_U12;5'ss_score=3.2199;bsite_score=0.5520
CAAAATGCTCATATCCATTTCTTTATTCTTCCTTCCATCACCACACCCCTAAAAATACACCTCCCCATCACCCCAATCAC
CTCTTTGCAACTCCTTCCCCAATCAATATCACGCACTCCCTCCTCTCTACGCATCCCCTCCATCACCCAGGACTCCTCCC
CAGCTTTTTTTTTTTTTTTTTTTTTTTTTTTTTTTCTTCCCGTCCCGCAATTCCTATCTATTCTTACTTGACTGCCATCT
ACCTCTCTTCCT

>p.polycephalum_GT_AG_Contig29955.1_296,491_1
PWM=droso_gtag_U12;5'ss_score=2.1916;bsite_score=0.9506
GCCCATGAGCGTATCCTTTTATTCTTGAACATGTTGCCATATTTTGTCAGCTATCAAGCAATAAACGCCACTTTAAGCTA
CAATTCTTACCCATATCTAGCTCTCCATCCCTTATCCAATAAAACACAACATATATATATATATATTGGTATATTAACCT
GCACAATTATCTTTGATCTTTTGAAGTTGTTTTGCT

>p.polycephalum_AT_AC_Contig4108.6_3074,3145_1
PWM=homo_atac_U12;5'ss_score=2.8108;bsite_score=1.2099
CAATTTACAAATATCCTAATATTATCATCCGTAATTGTTATATTCTTCTTTAATAAGTCTACATATGGAAAC

>p.polycephalum_GT_AG_Contig4083.3_619,686_2
PWM=homo_gtag_U12;5'ss_score=1.5311;bsite_score=0.7993
GAATAGCTGCGTATCCCTCCTTTATCGTTTCACAAGTATCTATTCATTGACAATTTAGATATTCCAAT

>p.polycephalum_GT_AG_Contig2347.2_3068,3137_1
PWM=homo_gtag_U12;5'ss_score=1.5073;bsite_score=1.0238
CCTACAACTCGTATCTTTTTGTAATAAGTTCACAAGTTCCATTACTTTGATACTTTGCAGATACCAAAAT

>p.polycephalum_AT_AC_Contig7069.1_3015,3271_1
PWM=mus_atac_U12;5'ss_score=2.7751;bsite_score=2.7931
AAATACCAAGATATCCATTTCTATAGCCCCCTCTCCTCTATTTCTTCCCCTCTCCCCTCTCTACTCCCTCTCCTCTACTC
CCTCCCCTCCCCTCTCCTCTCCTCTCCTCTCCTCTCCCCCTCTCTACACTCCCACTCTCTACACTCTCCCCCCTCTCTCT
TCCCCTCTATACCTCTCCTTCTATCCTCTCTCTCTTTTCCTCTTCTCCCTTCTCTCCTTATCTTTTTTGCTCCTTGACCT
CTAGCACGTGCTCATTT

>p.polycephalum_AT_AT_Contig534.6_1377,1463_1
PWM=homo_atac_U12;5'ss_score=1.9087;bsite_score=0.7625
CATTCTTCGAATATCACAATAAATATTAGGAGTGAGAAGAGGTGGGATGCCCAAATACCTTCTTTGATCTACACTATAGA
TATACAT

>p.polycephalum_GT_AG_Contig153.2_716,798_1
PWM=mus_gtag_U12;5'ss_score=1.5664;bsite_score=0.7911
CAGACAAACAGTATCCTACACGACATTTTTGAAAGACAGGATTTTAAATTACTTACTTGATCAAAAAAAAAAGAATTTTT
TCA

>p.polycephalum_AT_AG_Contig3375.4_1962,2030_1
PWM=homo_atac_U12;5'ss_score=1.8686;bsite_score=0.8622
AAAACGGCAGATATCAAATTGCTGCAAAGCCACATAAAATTTTACTTGATTTAAAGAAGACATTTATGA

>p.polycephalum_GT_AG_Contig11462.2_128,446_2
PWM=mus_gtag_U12;5'ss_score=1.5060;bsite_score=1.2508
CTTTGTCATTGTATCCTAACCCTTGGGTTGTGCATTTTGACTCGCTAAAAACAATATACTCTACATAGAAATATTGAAAC
ATATCTTCAATACCGTTCCACAGTTCTCACTTTTTTTTTCTCGTGTCTTGTATTTACAATATTTACAACAATAAATTTAA
CCCGTAAAAAAAAAAAAAAACAAAGTCTGCCACCTGCCGTGCGCGGTGTGCGAGGTGCGCAAGAGTCTGTGAGGGTCCGC
GAGAGTGTGCAGGGTATGCGGGGGAGGGTGCGAGGAATTATTGCTTCGTGATCTTTGACAAATTTTTAGGGCATCGGCT

>p.polycephalum_GT_AG_Contig9337.2_896,978_2
PWM=mus_gtag_U12;5'ss_score=1.5137;bsite_score=3.3422
GACAAGCTCAGTATCTTTCTTCCTCTTGTCGTGTGATGGACTAGCTAACAAAATCTCCTTGACCCTTGCTAAGATATTCC
TTC

>p.polycephalum_AT_AG_Contig5425.3_1341,1418_1
PWM=mus_atac_U12;5'ss_score=2.8131;bsite_score=1.0512
TCTGGATGCAATATCCAATATGCCGCAGGCCTTTCCTCTTCATTTAAAAACACCTTGACCCACTTCAGGTATCTCTCG

>p.polycephalum_GT_AG_Contig1185.6_1148,1249_2
PWM=homo_gtag_U12;5'ss_score=1.6435;bsite_score=1.8707
GGTCATAAGAGTATCCTCTCTTCTCTTCACCGTCCCATCCTCTCTCTCTCTCTCCCCCCTACTGCTTGCATTATTAGACT
TGACCTAATTAGACATGTCAGC

>p.polycephalum_GT_AG_Contig4309.1_1784,1871_1
PWM=homo_gtag_U12;5'ss_score=1.5685;bsite_score=0.7588
CCTTTGATGCGTATCTTTTGTTAATCCTCTTCTAAACACATTTCACAAACACTCTAACTTTCGTTGAGATCTCAAAAGAG
ACTCCGTA

>p.polycephalum_GT_AG_Contig2496.9_1414,1590_1
PWM=homo_gtag_U12;5'ss_score=1.6153;bsite_score=1.7056
ATGCAGATGCGTATCTTTTTTCCCTTTGTTTTATCATTCCAATCCATTTGTTTTCTTACGCTCTCTTCTTTTCCTTCTTC
ACATCTCATATTTACAGCTTCTATTCCTTATAAATTTATTAAAGCTGCGAGTAAATCATAAACATGCTCTTTTCCTTGAT
ACAATAGAAATTATTAT

>p.polycephalum_AT_AA_Contig1390.4_1137,1604_2
PWM=mus_atac_U12;5'ss_score=2.8887;bsite_score=1.1148
AGAGCCAGGAATATCCACTTTTACTATGTCCATTCTTATTCCTCTATCGTTCTCACCCTTTCTTTTAGCTCCCTCCCTCA
CGCCTCCCACCTCTTCTCACCCCCTCTTCTCGCCCCCTCTTCTCACCCTCTTCTCACCCTCTTCTCGCCCCCTCTTCTCA
CCCTCTTCTCGCCCCCTCTTCTCACCCTCTTCTCGCCCCCTTCTCGCCCCCTCTTCTAGCCCCTCTTCTCACCCCCTCTT
CTCGCCCCCTTCTCACCCTCTTCTCACCCTCTTCTCGCCCCCTCTTGTCACCCCCTCGCCCTCTTCTCACCACCCCTTTT
CTCGCCCCCTCTTCTCGCCCCCTCTTCTCGCCCCCTCTTCTCGCCCCCTCTTCTCGCCCCCTCTTCTCGCCCCCTCTTCT
CGCCCCCTCTTCTCGGCGCCCTCTCGCCCTTTCCTCTCATTAGTTTGACTATTGTCAAATATTCGACA

>p.polycephalum_GT_AG_Contig8371.2_309,494_2
PWM=droso_gtag_U12;5'ss_score=1.5476;bsite_score=1.4882
CATGGAAGTAGTATCCCTTTCTTTTCCTTCTCTTTCTTTTCCTTCTCTTTCGTTTCCTTCTCTTTCGTTTCCTTCTCTTT
CGTTTCCTTCTCTTTCGTTTCCTTCTCTTTCGTTTCCTTCTCTTTCTTTTAATTTCTTTCCCTCTTTACGGTGCTTGTTT
CTTTGATATTTGTCAGCGAGTCCGCC

>p.polycephalum_GT_AG_Contig1562.1_8055,8165_1
PWM=droso_gtag_U12;5'ss_score=2.1998;bsite_score=0.5594
AAACTGTGGTGTATCCTTTTCTCCTTTTCTCATCTCTCCTCCCCCCCCCCCCGCCACTTTTTTGATTTCTTTTGACTTT
TTTTTTCTTGATTTGCTTCAGTTTCTAGACG

>p.polycephalum_AT_AA_Contig3734.4_885,963_1
PWM=homo_atac_U12;5'ss_score=1.9119;bsite_score=0.8362
CAACAACCACATATCTCAAATTGTATCGCGAAATTTTATAATTTGTTAATTTTCTCTTACTTTAACTAAGAAAAGGCTG

>p.polycephalum_AT_AG_Contig801.11_497,593_2
PWM=homo_atac_U12;5'ss_score=1.9710;bsite_score=1.1154
TTTCGAGGTTATATCTCAAGTCTTTGTTTTTTTTGTTTGGTTTCTTTTTTTTTTTTTTTTTTTTTTTTTGTTTTTTTAGCA
ATGGAAGATATCCTACA

>p.polycephalum_AT_AA_Contig128.13_5639,5738_2
PWM=homo_atac_U12;5'ss_score=2.7465;bsite_score=0.5893

CATTGGCCAAATATCCTAAAATAATTGAAGTTGAACTTTCACAAAAGTAAAAATTACCTCTAGTCACGAACGGAGAATGA
CATTCACCAAATTTATCGCA

>p.polycephalum_GT_AG_Contig8018.3_942,1134_1
PWM=homo_gtag_U12;5'ss_score=1.6474;bsite_score=1.4781
AGAGTGCCCTGTATCCTGCCCGACCCCCCCCCCCCCCCCCCCCCCCGCCCCTTCAAAAATTGACATTGCCGCAATTTTT
TCTTACGAAACCTAGCGCACGTACGTGATTTGTTGTTACGGTTCGCCCCCCCCCCCCCTTTTTTTCCCCCCCCCTCTCCT
TTTTGAATTTTGATACACAATAGATTTTCCACT

>p.polycephalum_GT_AG_Contig7639.2_3234,3606_1
PWM=homo_gtag_U12;5'ss_score=1.5226;bsite_score=1.6080
AAGTGGAACCGTATCTTTCTGCAATATTTCTTGCTATTTTTCTTGCTATTTGTTGTTTGTTTCTTGGACCTCAGCGTTAT
TGTTTGCATTTTATATCATATGTATGTTCAGTGATTCACATTTCTAAATACTTGCCAGTTTTCGTGGTGTTGCATACATA
TTTTGCAATTGTTTGCATGGCGATATGCATCACCTACACTATTTTTCTTGTTCTAAATGCTTTTCAATTTGGTTTAAAAA
ACAATCGCACATTTATTACCACCTTTATTGTCCTAAATAAATCACTGCAAGTTTTCCATTGCCCATTGTATTTTTATCCT
TTCTGCCTTTTACCTTATTGATATTATTTAGTTGATTATTTAGAAATGCCACG

>p.polycephalum_AT_AG_Contig3219.5_2023,2095_1
PWM=homo_atac_U12;5'ss_score=2.9983;bsite_score=0.5687
TTTAAAAGAAATATCCTGCAAAAAAATACGAATGTATTTACGAATATCTTTTACCTCAAGAAGATATGCTAAA

>p.polycephalum_GT_AG_Contig2714.5_1969,2048_1
PWM=mus_gtag_U12;5'ss_score=1.5020;bsite_score=1.0904
GCAGTATTCCGTATCTTTAATATAATTTCTCTACTCTTCACAAAAACGCATATGTTTTTGACTACATCAGACAATTTCCC

>p.polycephalum_GT_AG_Contig10303.2_392,556_2
PWM=mus_gtag_U12;5'ss_score=1.7630;bsite_score=0.6330
CGCACTATTTGTATCCTGTAAACCCAAGACTTCTTTGGGTCCGTTCGAAAGTAGGCTAGGATTAGGCCATAGATCACGCA
GACCATTTTGATCTGCCTATTTTGCCTATTTTTCAAATGGACCCCTTCTTTTCTGTTCTTCTTTGATTCCTGCAGGTACG
TACGA

>p.polycephalum_GT_AG_Contig464.3_320,519_2
PWM=mus_gtag_U12;5'ss_score=1.5787;bsite_score=1.2479
TCGAGGGAGCGTATCCTGCACTCTTCATCAATGCCCTGTCAAACATTACCATTGCCATGCACCAACAGAATCGTTGCTTG
ATGCGAAGTACTGGGAGGGAGTATAACAGTACCACTTTTTTATTTATTTATTTAAACACACTTTAACGTTCTCCTTTCCT
TTATATGCTAATGTCTATGACGAAAAAAAGAGACTACGCC

>p.polycephalum_AT_AT_Contig4447.4_2524,2624_1
PWM=homo_atac_U12;5'ss_score=2.8044;bsite_score=0.8592
TATGGTGCGAATATCCCTCAAAGTGATGCGGCCAATGGGTAGTATAAGTATGTTAAAAATCGGTTATTACATTCTCTTAG
ACCACGTGCATAAATTCGGTG

>p.polycephalum_AT_AG_Contig7696.2_2200,2306_2
PWM=homo_atac_U12;5'ss_score=1.8709;bsite_score=1.1904
ACATTATAAGATATCTAATAAGCACATCACCAAATGATCGGTGTCATTTTATTACTTCTTGAATGATTTGTCATCAGCAT
AATTTGAAAGATCAAAGATATAGCATT

>p.polycephalum_GT_AG_Contig3219.5_2146,2471_1
PWM=mus_gtag_U12;5'ss_score=1.5852;bsite_score=1.3032
GGATGTTTCAGTATCTTTTAAAAATGAATGGGGGAAGGGAGGGTCCGCGGGACTCGTTGAAGGTCTGCGAAGGTCTGCGA
AGGTTTGCGAAGGTCTGCGAAGGTCTGCGAAGGTCTGCGAAGGTCTGCGAAGGTCTGGGTCTGCGAAGGTCTGCGAAGGT
TCGCGAAGGTTAGTAAAAGTCTTCTATCATTCGTAACGTACAAAATTCCTCAAACGTACTCAAACGCCTTCGTAAGTCTA
GTGGCGAAGGTCCAAAAATGTGGGGTAAGGGAAGGGAAAGATAATATCCATACGTGACGCTCTTTTACACAAATAGATTT
GCCAAC

>p.polycephalum_GT_AG_Contig10563.1_1167,1292_2
PWM=droso_gtag_U12;5'ss_score=2.1493;bsite_score=0.8276

CGACACCTTTGTATCCTTTTGCATACTAGTATTTGGAAATCATCTCAAAGTATACAATTATACACAATTTCAAATCTCAC
GACATATAACATCACCATATCGTTGACACGTGGTAGATATAACTCC

>p.polycephalum_AT_AC_Contig4108.6_1657,1853_2
PWM=mus_atac_U12;5'ss_score=3.1186;bsite_score=0.8904
CCTTATACAAATATCCTTAAATCTATCTTTTTATTGAATAAAAACATATAGAAATTAGCGCGGCCTTAAAAGCCTTTCAG
CGTACGTGAGGTAGATAGCACAAGGCAAGTGTAATTAGTGCATATATTTCTACAGATTCCTCTCTCCCTCCCCTCCTCTT
TTATGTACTTCTTTTACCACCCCATACTTACATACCT

>p.polycephalum_AT_AA_Contig1341.4_1331,1456_1
PWM=mus_atac_U12;5'ss_score=3.4350;bsite_score=1.9407
CCTTGTCTGCATATCCTTAATTTGATTTTCCTTCCTCCCCCCCCCCCCCCTTTTTTTTCTCTCCCTACAGGAATCATTTT
TTTGTCCCTAGTTCTTTTTTTTTTGACAATAATAAAAGATGTGCGA

>p.polycephalum_AT_AG_Contig564.5_356,516_2
PWM=homo_atac_U12;5'ss_score=1.5145;bsite_score=2.5216
CTTTAGTTCCATTTTTTAAGGGCCCCAAAAAAAAAAAAAAAAAAAAAAAAAAAAAAAAAAAAAAAAAAAAACTATCTACT
TGTCCATATGTCTGCGAAATAGAAATTGTGCACAAAGGTTAACCTCTCCCTAGGCTCTTTTATAGTGGTAGATATTCCAG
C

>p.polycephalum_AT_AC_Contig1997.2_2175,2351_1
PWM=homo_atac_U12;5'ss_score=3.3626;bsite_score=0.5679
GTCGAGTTTTATATCCTTATAACTTAATTTGATTTTAATGTGCATCCTTAAAAGCCACAATGGCCATCTGTTATAGCTAA
AGTTTAACTAAATTGCCGAAAGCTAAAGTTTAACTAAATTGCCGAAAATCCCAAAATTAGATCCTAAATGCCTTTGATCC
ATTTAACATATCAGAAA

>p.polycephalum_AT_AC_Contig5233.3_2886,3113_1
PWM=homo_atac_U12;5'ss_score=2.9095;bsite_score=2.2956
TGTTGTAGAAATATCATTTCACTTTACCTTAAGAGCGCAACACCCAAAATGTTGTGAAAAACTACAAAAGCCATTGGTCG
TATAGGATTAGTCTCAGCAACGAGCGCTACGTGTGAATATTTTGTTTATTATACTACACAGGCGTTTGGTAAAAAAATCA
AAAAAAAAAAAAAAAGCACATCTCCCCCACCTTTTTAATACATTTTGACTTTGGAGACATAATGACAA

>p.polycephalum_AT_AA_Contig10068.3_184,418_2
PWM=mus_atac_U12;5'ss_score=3.3703;bsite_score=0.8863
CTCCTTTAAAATATCCTTTAAATAAATAAATAAATAAATATAATATAATATAGTATACATGTTATTATATTCATGGCTAT
ACGCGTCTACCTTGCGTCTTCTTATCATCAATTCTATTATCCCCTAAGCTCATGATTCTGAAAATTATAATTTGATTGCA
TTGCGTATTTCGCTTCTGTTCTATCACACTCGAGTCCTCAAATAAATTCATCATGACAAAAGCAAGTATTACTCA

>p.polycephalum_GT_AG_Contig1752.6_996,1539_1
PWM=homo_gtag_U12;5'ss_score=1.9298;bsite_score=1.0228
TCATCGAAGTGTATCCTTTATACTGGTGATGTTACTTGGTTAATGCAATTGTAATTCATTGCATAGCAGAAAAAAAGAAC
AGTTAGCCATCAAAAATAATTGCAGCTGTGAAAAGGTGTTTACGCTAGTTCTACAATTTGATCACTCTTTTTTGCAATTG
ATCAATGGGCATAAAGTAAAATTTAGATTATATGTTATATGGACCTAATTTCACAGACGAAGATGTAAGACTTTTCGGGT
CTTTTTTTCGAAAAGCATGTGTCACCCACATTTTTCCTTTATAGTTCAGAATTTCGCCTGATCTTCATGCATTACACTTT
CACACATTACCTTCATTCCAATATACTATCGCACAATAGCAAGAAAGACACTAAACTCACGGGAACTGCTTGTGTTCCAC
ACTCAACACTATTCCTTTAAAATGCCGTTTCAACAATTCACCTACAAATATCTTAGCATCCCTCCCCTCTCCCAACAAAT
GCACTCCCCAACTAGTTCCTCCTCTGCGCAGAATTGCACATGACCCCTAAAAAGATACTAGAAT

>p.polycephalum_GT_AG_Contig2078.2_984,1122_1
PWM=homo_gtag_U12;5'ss_score=1.8562;bsite_score=0.8108
GAGGTAAAGAGTATCCTTTCTTTTACCTTCTTGTCCTTAGTATCTCTTCCAATCCTTCTACTGCCCTCTCTTTAGTTTCC
TTTCTTTCTTTGCCTTTAAGTTTATTTTGCTAAATTTGAGTATATATAGAAATTTTATT

>p.polycephalum_GT_AG_Contig7559.2_876,998_1
PWM=homo_gtag_U12;5'ss_score=1.5953;bsite_score=1.1738
GTTCGTCGTCGTATCTTTTTACATGTTTTTTTATTCACTGCACATTCACACCCCACCCCTCCCACGCACGTTTGATCATT
TAGTATTTTATTTTTCTTCTTTGATCTTGGAAGGTATGATGAT

>p.polycephalum_AT_AA_Contig325.6_2626,2732_2
PWM=mus_atac_U12;5'ss_score=3.3544;bsite_score=1.4529
ATGGTATTTGATATCCTTTATTGCCCTTTCCTCTCTCCTCTCTTCCCTCCTCTCCCCCCCCCCCTCAAATTAGAAATGTA
ATTTTGATCATTACCAAATATGGTTGG

>p.polycephalum_GT_AG_Contig14690.2_820,911_1
PWM=droso_gtag_U12;5'ss_score=1.9498;bsite_score=2.7174
TTTAAGTTGGGTATCCTTTTTTCTGCCCCTTTTCCACCCCTTTTCTATTGCTAGCTTTTGTGGGACCTTGACGGTGCTAT
AGATATTAGAAA

>p.polycephalum_AT_AC_Contig6117.3_1542,1731_2
PWM=homo_atac_U12;5'ss_score=2.8311;bsite_score=1.1214
ATGGGTTATTATATCTTTATAATTTTTCTTTTTGTCTTTCTATTTTGAAGGGGAACTCAATATAGTTTATAAGCAGGGAA
AATAGATAAGTTCGTAACTAAAAGTTCTAACCACTCGTAATCATCTTTAATGTTTATTTATCATCTTTAGTAACTTTCGG
AATCACTTTGATTTGCTAACCATCTCAAAG

>p.polycephalum_GT_AG_Contig5111.2_489,783_2
PWM=homo_gtag_U12;5'ss_score=2.0250;bsite_score=0.9809
AAATATCACCGTATCCTTTCATCAGCTTCGCCACCCGTCTAAGGCTTGTTGTAACAAATTCAACCAAATACAACTTTGTG
GATTTAAAATTGGTTCTTAAAAAACCAGTTGCATTTCTAGTTGTAAAAATTGTTGCACTTTGTTTGAGCTAGCCGGTTTG
GTTAGATAAAAGCTTGTTTATCAAACTGGCCACAATTACTTCTACAACCTTAGCAGTTGTAAAAAAGCCCAGCAAAGTGG
GCATGCCTGTTCCCTTTCCTTGGTTCATTAGTTTGAATAAATTAGATTTGTAGAT

>p.polycephalum_AT_AA_Contig1448.8_808,919_2
PWM=homo_atac_U12;5'ss_score=2.5484;bsite_score=1.1566
ACTCGCTATCATATCCCAAAATTTCTGACCTCCATTTTCCCTTCTTTCTTTTTCTTCCTCCTCCCTCCTCCTCCTCCTCC
TCATTTTTTTGACCTATGCTAAACATTTGCCA

>p.polycephalum_GT_AG_Contig4091.3_2125,2209_1
PWM=mus_gtag_U12;5'ss_score=1.7642;bsite_score=0.8842
CCAATTCAGTGTATCCTTAATTTAAAAAGAGAGATCGAGCGAAAGTTGCTTGGTGACAGCGTTGACTCTTCTCAGTTCTA
CTGGT

>p.polycephalum_AT_AC_Contig2584.3_1518,1611_2
PWM=homo_atac_U12;5'ss_score=2.8154;bsite_score=0.9152
CGATATGGACATATCATTATTGAACTTTTTTGCTCGTCTCATTTTCCCTCTTATTCTCTTACTTTTTTCTTTGATTTCTG
GTACCTGTGACACC

>p.polycephalum_GT_AG_Contig2980.4_4247,4380_2
PWM=homo_gtag_U12;5'ss_score=1.9259;bsite_score=1.0827
GGCGGAATTTGTATCCTTCACTTCAGTTTTTTTTTTTTTTGAAAGCAAAGGGTACATGGTTTGTTTTCAGTGCTTTTTTT
CTGTTTTCCTTCATTTTGTTTCTTCTTGTTTGATACGTTGTAAGATACTTCTGG

>p.polycephalum_GT_AG_Contig108.1_318,447_2
PWM=homo_gtag_U12;5'ss_score=1.5144;bsite_score=0.7329
ATAGGATGTTGTATCTTTCTTTTCATTGTTTTCCTTTCGATCTCTTTGTGTGTAAATTCTTTTTGCACTACATTTTGTGC
ATTCTTTCACAAAAAGAATACACACATTTAACTGTCACAGATATAATTTA

>p.polycephalum_GT_AG_Contig1739.2_2200,2569_1
PWM=mus_gtag_U12;5'ss_score=1.4749;bsite_score=1.4220
TACACTGGCTGTATCCCCTCCACCCTCCTCCCCCCCCCCCCTTCCCGTTCAAGATTTTTGAGCGAAAATGGTTACCTGGG
TAGCGCACCTTTAGTGCGAGGTTAATTAAAGAAAAAGCAGCTTTACCCAAGGAGGGGCGAATTTATCTTTTTTACTAGAT
ATTGGTTGGTTCCATGGTGCTCCTGTGGCGCTCTCCCCTCTCCCGCGCACCACATTTAGTGCGAGATTTATTAAGGAAGA
AGACCAATGCTCCAGAAAGCCCTCACCCAAGGACTTTTTCAGCCGAAAATGTTTGTTGCGTTACTTTAGCAGCTTTTTTT
TCTTATCTCTTGCTCCTTTATTTTTCGCGACTTTTACCAGATATTGGATG

>p.polycephalum_AT_AC_Contig2122.2_373,446_2
PWM=mus_atac_U12;5'ss_score=2.6868;bsite_score=3.7252
AGAAAGAAATATATCCACTAGATTAGCTTCCCTTTCCTTTCCTTACCCTTTGATTCATTCTCACATTTTAATCA

>p.polycephalum_AT_AC_Contig3022.4_5940,6211_2
PWM=homo_atac_U12;5'ss_score=3.5287;bsite_score=1.5678
TTTTGCCAACATATCCTTTTTTTTTTTTAAAAATAATTTATTTACTTGTACCATCATATTATCATATCTTTTAGAGTGCA
AGTATACCTGTGCATTTCTTTCTCGATCCCATGCCAAAAAAAAAAAAAAAAAAAAAAAAAAAAGGATTGGCGTGAATGGTT
ATAGTTTGAATGTAAAAACAGTATGCACCTTTTTATTTTATATTTATAAAAATATTTTAATTTTGTTGTGTTCTCCTGAT
CAAAATCTTTGACACAGCAAACAAATATTCGT

>p.polycephalum_AT_AA_Contig3933.4_800,938_2
PWM=mus_atac_U12;5'ss_score=2.1789;bsite_score=0.9983
CCCAAAAAAGATATCCGACAAAAAAAGCTATTTTTATTTTGTTTTGAGTTTTTTTATTATTATTTGTTTTTTTTTTTTTT
TTTGTTTTTTTTTTTGTTACCTTTAATTAAATTTTCGTACCTTTAATAAATTTCGACCT

>p.polycephalum_AT_AC_Contig28085.1_289,407_1
PWM=homo_atac_U12;5'ss_score=2.8277;bsite_score=0.7366
TCCATGATTCATATCCCATACTTTCCCCCCCCTCTCGTTTTTTCATCTATTTCTTTCTGTCTTTTCCATTCTCTTTCCTG
AAAACGAACAAGGATTGACCCAAGGTGACATATTTGGAA

>p.polycephalum_GT_AG_Contig224.14_437,605_2
PWM=droso_gtag_U12;5'ss_score=1.7053;bsite_score=1.3525
TCGATGAAGGGTATCCTTTAATTATGGCTACCATGGGGAAGGTAGAGGGTGGGGGGAGGAGAGGGCGAGGAGGAAGGAGA
TGATAAGGGAGGAGATAAGGGGGGAGGGGAGAAACATGATAATAGGCACTGCATAACCCTTATTTGATTCAGAATATAGA
TATGGAACG

>p.polycephalum_GT_AG_Contig6414.1_1558,1638_1
PWM=droso_gtag_U12;5'ss_score=1.8388;bsite_score=0.6749
GTTTAAGGTGGTATCCTTTGTCTCTATGAATACAAGTTTGCTTTGGCACGACTATCTTTTACTCTATGAAGTTCACAAGC
C

>p.polycephalum_GT_AG_Contig12108.1_1165,1266_1
PWM=mus_gtag_U12;5'ss_score=1.6914;bsite_score=2.2274
TGATGAAGCAGTATCCTCCTCTATTATGCGTTTTCACTTTTCCTTCCTCCATGCTTTTTTGACTTTCCTATTGTCTCCTT
GATATCTTGAAGATATAATTTA

>p.polycephalum_GT_AG_Contig5381.5_957,1116_2
PWM=homo_gtag_U12;5'ss_score=1.6198;bsite_score=2.3693
CTACAATTTCGTATCTTTTCTCCAACCCTTCCTCCGCCTCTGCCCCCGATTCATATCCCTCCTTTTTTTTTCTTTTCTTT
CCTTACTTCCCCTCCCCCACCCCTCCCCTCCCCTCTTTACCAGGAAGAAAGCAGGTTGACCTAAATGTAGATACTTGGAT

>p.polycephalum_GT_AG_Contig3453.2_1313,1397_2
PWM=homo_gtag_U12;5'ss_score=1.4751;bsite_score=0.9774
TTACAATGGGGTATCCTTCCGGTCATTGTTTTAATCTTTATTATGTTTGTTACAAAACGCTTTAACACTACCAAGAAAAG
CTTCC

>p.polycephalum_GT_AG_Contig6963.3_1518,1606_1
PWM=mus_gtag_U12;5'ss_score=1.4932;bsite_score=1.3327
TGTTAAAAGTGTATCCATCAAATTAATCAACTAACTTGCCGTCACCACCATGTTTTCTTTGTTTTCTTTGACAATCCAGA
TACCAAGTG

>p.polycephalum_AT_AC_Contig9044.1_791,998_2
PWM=homo_atac_U12;5'ss_score=3.2319;bsite_score=1.2841
GTAATCGATGATATCCTTCTAGTAATTAATGACCAAATAATAATGATTTTAATCGCCCTTTTGAAAGATTGCAATCGCGT
GAAACATATGATTCAGCTTGTCATCGTGATAATTAAAATGAGTACAAGTACACTTCGGCTTCCAATAAATATAGTACAAC
GAAATCGTTGCCTCAGAATGATTCTTTAATTGTCATACATATAGTTAG

>p.polycephalum_AT_AG_Contig1739.1_6767,7153_1
PWM=homo_atac_U12;5'ss_score=2.8976;bsite_score=3.2328
AACTTGGCCAATATCTTTCACCTCTAATACTTGATTATTCCTTATCAATTCACTTTTATGAGCACATTTGAATCGGAAAT
TATATAGCTACCGATTTCTGTTGAAATTTAAAATACGGGATTATTAAATTAAGGTGGCACAACGTTCATTCAATAAAGGG
GAAAAAAAACACGTGCGTAACATTCGAAAAGGGAATTTCGCACCCGCGCGCATGCGTAACTTCCCATAAATGGAGTTTCG
CACCCGCGCGCATGCGTAACATTCCCATAAATGGAGTTTTATCTCCAAAATAAGGTGGCACGCTTGTGCGTAACATGAAC
CGTTCGTGTTACGCCTGTGCCACCTTAACATCAAGAGCGATTGTTGATTAATCGCAGATATCATAAT

>p.polycephalum_GT_AG_Contig1197.7_1847,2354_1
PWM=homo_gtag_U12;5'ss_score=1.5577;bsite_score=0.9985
AACATGAATTGTATCTTTTTTGCATTTTCTATTTTTCAAAGCCATGAAAATTTTGAGGATTGAATTAGTCTTTTTTGCAG
AGACCAATTTGAAATTCAATTTGCAAAAAAAAACTCGAAATTTTGAGTTTGATATTGTATTGTGCACGTTTTGGTCATGAA
AAAGCCTTCCTAGAAATACTAGCACAGACTGGTTTTGTCATTTTAGGCAAAGTTTTTGCAATTTGAACATGAAATTTGGC
ACGGTTGACCGTGAAAAAGAATAAAGGATAAGGAAGTGTATGTACTAAGTCTCTCTCTAGGAAAAAAAATGCCAATAAAA
AAAGTTATGTTCAAGTGCATTTTTTTGGCACACAGATGATGGTCTCTAGTTACAACTTTTGCACCCAATTAGCAAACCTT
CAGCTTTTTTTTTTGTTGCTATTTTAGTTTTTTTTGTGGTTGAAATTTTTTAAATTCATTTTTCATCTAGTTTTTATTTA
TTTCTGTGACCCTTTCAGACTAATTTGT

>p.polycephalum_AT_AC_Contig1169.4_5120,5227_1
PWM=homo_atac_U12;5'ss_score=2.6249;bsite_score=0.8547
CCTGATGCTGATATCCCTATCTCCCCCCCCCCTCTCTCTCTCTCTCTTTCAACCCTTCGTTATTTATTTCTTCCTGATTT
TTGTTTGATGGTATTTACCTCTCGATTT

>p.polycephalum_AT_AG_Contig13269.1_1175,1456_2
PWM=mus_atac_U12;5'ss_score=2.8445;bsite_score=0.5723
ACTAGTTTCTATATCCAATCTATTCATTGGGTTTATTTGACGTCAGAAAAATAGTTGTTGTTTTCTCATTTTCTGATGTT
GTCCGTAAGCAGTAAAGTCCATTTTAAGTTTTATTAGCATTTTGTGAACGATAGTTGACATTTTTAATGTTAAAAACTAA
GACATTAAATTATGGAAAATTATCCTCTACATCTTCTTCTCTCTCTCCTCCCCCTTCCTATCTTTTCTCTTTTCTCTCCT
TCTCCTTTTTTCTTTTCTTTGATGTTGTGCAGGTTTAGATAT

>p.polycephalum_AT_AA_Contig1327.4_7252,7592_2
PWM=mus_atac_U12;5'ss_score=2.9635;bsite_score=1.5135
GAAACTGATAATATCCTAGCACAAAAAATAAAGAGATTCTCTTTTCATTTCTTTTCATTCCTTTTCATTTCTATTTTTTC
TTTTCTTTCTTTTCTTTTCTTTTCTTTTCTTTTCTTTTCTTTTCTTTTCTTTTCTTTTCTTTTCTTTTCTTTTCTTTTCT
TTTCTTTTCTTTTCTTTTCTTTTCTTTTCTTTTTTTTTTATATAGTCGTTTTAAACAACTTCCGCACATTTGATCAGTTA
ACGGTAAGTTTTGAGTTTTCTCAAAATAATTTTTGGAAACCTATTGTAAATAAAACGAAGTTTTTTGCCTAGCATCTTTG
ATGTAATTCAACCACAAAACG

>p.polycephalum_AT_AC_Contig969.16_887,998_2
PWM=homo_atac_U12;5'ss_score=3.1877;bsite_score=0.7575
ATAAGAATTGATATCCTTGTACAATTCATCGTTTAATTCTGCAAAATAATACGGAATTATTAGATAATTTGAATAACACT
TATTTTCTTTAATACTGCCAACGTATGATAGG

>p.polycephalum_GT_AG_Contig781.9_4125,4296_2
PWM=homo_gtag_U12;5'ss_score=1.7878;bsite_score=0.9371
GAAGTGGATTGTATCCTTATTTGTTTTCTCTCCCTCCTCTCTCCACTGACCTTCCCGCCATTCTCTCTCACCTAACCCCC
CCTCCCTTTCCTCCTTCCTTCAGCTTCTCACTCTCCCTTCTATTCCGTCTACTCTCTTCCTTTCTGTCGTGACACTTCAA
AGAGATGTTGCT

>p.polycephalum_GT_AG_Contig58551.1_879,1532_2
PWM=droso_gtag_U12;5'ss_score=1.7737;bsite_score=0.6796
GTAAATGAAAGTATCCTTTGTTCTCTCTTCCCTGTCTCTCCTTATGTCCCCTATCCTCCTTCTATGCCATCTATCTCCCC
TTTTGAGGTACGAATTTGGCTGATTGGTTGTAATTGGCTAATAAAGTTGGTTTTGGGCTGTGTAACCATTTTTAGCTAGT
AAAGGTGGTTTTGGCTGTAAAATTTGGTTTTGGGAAAAAAACTAATTTTGGTTAATTAGATGTTTTTGACTGGTTCAGCC
ATTTTTAGCTGAATCATTTGGTTTTGGTATAAAGTATTTATTTTTCAGGGTTAGAATTATTCAAAATACAAATAATTTTT
TGTTTTTACGTTGAATTAAAAACCAAAAACAGCCGAACCAGCCGAAAATGGCCGAACCAACTGAAAACGGCCGAACCAGC

CAAAAACGCCAACCAGCCAAAAATGGCCAAACAGTTGAAAACGGCTGAACCACCCAAAAACAGCTTAACCAACCACAAAT
GCCTCATGTATCCAAAACCAAATTTGGTTTAATAAACCAAGCTTGCTTAAATAAAGTTAATTTGGCTTAACAAAACGTAT
TTGCTAACATAACCGCACATGGCCAAGTTTTGTCCCTCTAGCACTCCTTCTGATTTGATTCATATTTCCTTTGAGTGAAT
GTAGATATCATATC

>p.polycephalum_AT_AA_Contig1244.9_547,627_2
PWM=mus_atac_U12;5'ss_score=2.7684;bsite_score=0.8300
GTTCCTTTTCATATCCGCACTCCAAAGCATTCTTCTACTCAACACTAATAATAAATTTTGACTCTCCGTAAATATGCAAC
A

>p.polycephalum_GT_AG_Contig9650.1_422,490_2
PWM=mus_gtag_U12;5'ss_score=1.6611;bsite_score=1.4680
CAATTAAATTGTATCCTATGTTCATCAACGTAGTCTACAATGTTCTTTGACAATCACAGATTTGACTAA

>p.polycephalum_GT_AG_Contig2629.5_4627,5410_2
PWM=homo_gtag_U12;5'ss_score=1.5100;bsite_score=1.0933
TTGTCGATGGGTATCCTTCTTTTCCCTTTTTCTTTTTCTATTCCTTTTCCTTTCCTTTTCCTTTTCCTTTTCCTTTTCCT
TTTCCTTTTCCTTTTCCTTTTCCTTTTCCTTTTCCTTTTCCTTTTCCTTTTCCTTTTCCTTTTTCCTTTTTCCTTTTTCC
TTTTTCCTTTTTCCTTTTCCCTTTTTCCTTTTTCCTTTTTCCTTTTTCCCTTTTTCCTTTTTTAACGCACCGTGGTGCGC
TGGTTTTAAGAGCGCTGCGCACGGTTTTTTCCTGCGCTCCCACTTTTCCCTCTCCCCCAAGCATCCACATCCCCCAAGCT
TTGCCCTACCCATCTCTCCCCCCATCTTCCCCCATCTCCCACCCACCCGCCCTTAAACAGTCTCCTACCTCAGCACAGCT
GCAGCGAGTTCTGCACAATAAACCGGTGCAAAGCGCTCTTAAAATTCCTTTTTCCTTTTTCCTTTTTCCTTTTTCCTTTT
TCCTTTTTCCTTTTTCCTTTTTCCTTTTTCCTTTTTCCTTTTTCCTTTTTCCTTTTTCCTTTTTCCTTTTTCCTTTTTCC
TTTTCCTTTTCCTTTTTCCTTTTCCTTTTCCTTTTATTTTTATTTTTATTTTCTGTTTATGTTCTGCTTCTTTTTCTTTT
TGCTTGTTGCCGAAATTCAAAGACTAGGAAACTCGATGTAAAAATGTTCGGATCGCTTTTTTTTTTTTTTTTTTTTGTTT
TTTTTTTTTTTGTTTTGTTTTTTCTCCCCATATCTTTTTTTGAAAATTCAACAGAAATGTTACT

>p.polycephalum_GT_AG_Contig11916.1_1070,1408_2
PWM=homo_gtag_U12;5'ss_score=1.7693;bsite_score=2.0357
GATGTGTGTAGTATCCTTGTATACCACTCTTTTCTTCTTCTTCTTCCTCTCTTGCTCTTCTTTCTAAGCCTACGTTGCCC
CATCGCCAAGTTCTCAAAGGAGTCCTCTTCTTCTTTATTATTCTGCTTTCTTTTCTCCACCCCTCTTCCCCGTCTCGACA
AGTGGCAGGTTATCTATGTTGTAAAAGGTGGGTAGTATCCTTTTCCCAGCTATTCATTCCTTCCTTCTCTATATTTTCTT
GTTATAGTTTCTAATATCTGTGCCACCCCTACAATCCCATCCACCACGTTCTCCCCTCCCCTACATTAGTTTTTTTAATA
CATTGTAAGATATATCGCT

>p.polycephalum_AT_AA_Contig2495.2_2193,2596_1
PWM=mus_atac_U12;5'ss_score=2.6581;bsite_score=0.6612
CAATACACTAATATCCGCCTCCCCCTCCCCCAAATTACTTTGTCTGCTTTGGTTAAAAAAGTTTACATTGTACATTTTTT
TTTTCCGTTCTAAATTAACCCAAGTATGTCCATTTTTGTAATAGGTGATAAATCACATTGCAATTTTTTTTTTCTTTTCT
AGTTAGGTAAAACAATTCTTAAAGTTACGAGGCTAAGGACGACGGTGAAAATTTTCAAAAAAGTTAACTACGTTATGGTG
AAATGTGAATGGCAAATTTGATATAAAATGAACAAATGAAGAAAATTCTATTTAATATTACAGTACACACTCCTTTTTGT
GTTCCGGCCAGTGCGCTCTCTGGCATTCTTGACAATGATATCTACTCTAGTAAAATTCAATTTATGGCACACAAAAAAAA
AAAA

>p.polycephalum_GT_AG_Contig188.4_3430,3502_1
PWM=mus_gtag_U12;5'ss_score=1.6279;bsite_score=0.6531
TATTAATGCCGTATCTTTTAGCATTTCTATTTTTGTCATTTGCCGTACTTTTACTCCATTTAGAAACTAAGCT

>p.polycephalum_GT_AG_Contig4437.4_230,303_1
PWM=homo_gtag_U12;5'ss_score=1.9697;bsite_score=0.6548
CAAAAGAATTGTATCCTTTCATGGTTATATTTGTCAAGTACTTCCGAATCTTTGATCACCAAAGTTTGCATCAA

>p.polycephalum_AT_AG_Contig15381.2_214,394_1
PWM=homo_atac_U12;5'ss_score=2.1245;bsite_score=0.9451
AAATTAGTTCATATCAAAAAAAAAGAAAAAAGAAATATATGCTTAGTTTTGTTTTGTTGATTTATTTTGTGTGGCCTGAA
AATTTACGAAAAGTTGATGTAAAAACTATACTTTGTGCTAAGTTCACCAATTTTAAACATTTATCTATGCCATAAGATTG
ACAATTCACAGATATCCCAAT

>p.polycephalum_AT_AA_Contig8413.3_461,542_2
PWM=homo_atac_U12;5'ss_score=3.4606;bsite_score=1.9083
GATACATACAATATCCTTCTCAATTGCTCTCGCCTCCCTTATACATAATAGTACCTCGTTGACGGTGCACAATTCCTCTG
CT

>p.polycephalum_GT_AG_Contig8534.3_154,400_2
PWM=droso_gtag_U12;5'ss_score=1.6308;bsite_score=1.2343
TGCTACTTTGGTATTCTTTTAACCTTATTACCAACCACATCACTTTTTTTCTAAAAATTGTAAAAATATATTTATTGAGC
TACCTCTATTGCATTATATAAATAAAATTAATTCTTCAATTTAATGAAAGTTAATAAAAGTACGTTGGTATTCTGGCCCT
TATACGACGGTGTAGTGGCGTGTTGCGGTGCGTGTTCTGCCCTGTGACTGATCTCCCATTGAATTTTTTTATCACAGGCA
GATAAAG

>p.polycephalum_GT_AG_Contig35.9_167,293_2
PWM=homo_gtag_U12;5'ss_score=1.6192;bsite_score=2.0170
AATTCAAACCGTATCCCTTCCCCTTTCCTCTAATGTTTACAAGATTTGAGAAAGAGTTTGTATGAGAACTTTAGTTTTAT
GCCTCTTATTCTCTGGGCATCCTTGATACAGCAAAAGAGAAATTAGA

>p.polycephalum_GT_AG_Contig12868.1_492,626_1
PWM=homo_gtag_U12;5'ss_score=1.8660;bsite_score=1.8878
AACATGATATGTATCCTTTATGTTATTACATGAAAATATTACACATCAGTAACTTCGGTTTATTTCAACCCTCGCACCGT
ACCATGCAACAATATCATTTCTTCATCTTCATTGACCATTAAAAGCTCTGGTCCT

>p.polycephalum_AT_AC_Contig2706.4_1367,1601_1
PWM=droso_atac_U12;5'ss_score=3.8094;bsite_score=0.5913
AGGATGCTCAATATCCTTTTTTATTTGTTTCCCATTCGCATCCTTTCATAGTTTATTTTCCATTTAACAACTTGCCATTG
AGGATAGCGCTGCCTTCATTGCCATAGGCAGGTACCCTGAATTTCTATTCTTACCCCCGCATTTATTTTCTCATTTCCAT
TCGAAAAACCTCGTCTTCCTTTCTCGAATATCCTTTTGATTTAGCAATTCCTTGACTCTAGACACATACTATGGC

>p.polycephalum_AT_AC_Contig3918.5_2724,2793_2
PWM=homo_atac_U12;5'ss_score=2.4729;bsite_score=0.7653
TGGCACACCAATATCATAATGTATTTTTCCGTATGTGTGGTCCTCTTTGACTAGAAGTACATATTTTTTG

>p.polycephalum_GT_AG_Contig7778.1_2055,2204_1
PWM=homo_gtag_U12;5'ss_score=1.5482;bsite_score=0.9388
ACGCACCATCGTATCTTTTTTTGTCTTTTCTTTCCATTCATCGATCTGTGCTCTCCTCTCCCCCTTCTCCCTTTTCCTTT
CATTTCCTCCCCCTCCCCCCCCTCTCTCTTCCCTCCCGTCTCCTTTTCATGACATGAAAGATATGCATAC

>p.polycephalum_GT_AG_Contig8689.1_774,1087_2
PWM=droso_gtag_U12;5'ss_score=2.1002;bsite_score=0.9398
ACGTGCACGTGTATCCTTTTTTGACAAAAGATGACGTCATTCAGTAGCGATGACGTCATAGAGCTATATTTGGGATACGG
GCAATTTACAATGCACCATTTGTGGTACTAGACTCATTGGAATCCTGAAAAAATTCCTCATTTCAATTGTGACGAACGAA
ATTTTTCGTGACATCGCAAAAAAAAGGGGGGGGGGGGGGGGGGGATGAAAATAGAAAATACAATTTACAAAAATAATAAT
AACAATAATAATAAAAAAATAAAAATTATGATCCTAACAATTGTTTTATTTGATTAAATTATAGAATTGTAAAT

>p.polycephalum_AT_AA_Contig794.3_4432,5177_1
PWM=homo_atac_U12;5'ss_score=3.2265;bsite_score=1.6472
TGTTGGCTTCATATCCTGAGTAGAGAAGGAAGAGTGAGAGAGTGGAGAGTAAGACACTTAATTTTCAGTGAACAGGGTGA
CCCTAAGTATTATGGCTTGTATATCTTTTGATGCTAGCAGTATTTTTCTTCTAGATTTTGCGCACGTAATTATTGTGCAC
ACAATATTGTGTGGTCACTACAACACAACTTTGCACACAATTTTTTGTACAATTTCACTGAAAATTACAGAAAATTTGGA
GAGTGAAGAGACAAAAAGAAAAAAGAAAAGTACCTTGCCACTCTTTCTTTCCAGTTGGCAGTCCCATACCTCAGTGGGTA
ATCGATTCACAACATCTCTTGCCTTTGGTGACTTCATATGGGCATTGCTAACATGATCTATCATTCAGCAATTTTCGATT
TCTCGGTCAAATTTTGGCCTGAAAATCAGAAAAATTGCTTTAATAAATGAAGAATCGTGGAACTGGATTTATCTCCCTGT
TACACCCCATACAAGCACATGGCCCTCATACACATACACAGGGAAGTTCAGTCAAAACTGGCAGCTTGCCTCGTTTGTAA
ATTGATCAGTAACCAAAACCAAAGAACTATGAGGTCCTAATTTATAAAAAAAAATGAAGGTTGTGACTCATCATGTCAAC
ATATTTTAATATCAACTTGTTCACACAATAACATCATGCCCATTCTTTGGCTTGTTGTGCTCTAGCAAAGACTAGGATTG
ATTGATTATATAAGAAATATGACATG

>p.polycephalum_GT_AG_Contig5501.4_1097,1166_1
PWM=droso_gtag_U12;5'ss_score=1.9040;bsite_score=1.4111
TAACAAACACGTATCCTTTGAGTTAGCTTATTTACTTCACAATCATTGACCATTGGCAAGGAAACACCTA

>p.polycephalum_GT_AG_Contig5331.3_509,593_2
PWM=homo_gtag_U12;5'ss_score=1.4902;bsite_score=1.3676
ATCAGAGGACGTATCCATTTACATTTACTTGAGTTTTTCCTTACCGCGTGTATCATCATATTTGATTTCATTCAGGCATT
CTCCG

>p.polycephalum_GT_AG_Contig2706.2_610,1263_1
PWM=droso_gtag_U12;5'ss_score=1.7737;bsite_score=0.6796
GTAAATGAAAGTATCCTTTGTTCTCTCTTCCCTGTCTCTCCTTATGTCCCCTATCCTCCTTCTATGCCATCTATCTCCCC
TTTTGAGGTACGAATTTGGCTGATTGGTTGTAATTGGCTAATAAAGTTGGTTTTGGGCTGTGTAACCATTTTTAGCTAGT
AAAGGTGGTTTTGGCTGTAAAATTTGGTTTTGGGAAAAAAACTAATTTTGGTTAATTAGATGTTTTTGACTGGTTCAGCC
ATTTTTAGCTGAATCATTTGGTTTTGGTATAAAGTATTTATTTTTCAGGGTTAGAATTATTCAAAATACAAATAATTTTT
TGTTTTTACGTTGAATTAAAAACCAAAAACAGCCGAACCAGCCGAAAATGGCCGAACCAACTGAAAACGGCCGAACCAGC
CAAAAACGCCAACCAGCCAAAAATGGCCAAACAGTTGAAAACGGCTGAACCACCCAAAAACAGCTTAACCAACCACAAAT
GCCTCATGTATCCAAAACCAAATTTGGTTTAATAAACCAAGCTTGCTTAAATAAAGTTAATTTGGCTTAACAAAACGTAT
TTGCTAACATAACCGCACATGGCCAAGTTTTGTCCCTCTAGCACTCCTTCTGATTTGATTCATATTTCCTTTGAGTGAAT
GTAGATATCATATC

>p.polycephalum_GT_AG_Contig527.1_107,1476_1
PWM=droso_gtag_U12;5'ss_score=1.8434;bsite_score=1.1551
TCTTTGAATAGTATCCTTTAATAATTTCATCCATCACTGACACCAGAGTACACTAAGTGATCACCCCTTACTAACCCCCC
GCCGTGTTTCACCTGCACACTTATCTTGCTTGCTATTTCATGTAATAAATATTAATAATTAATTAAATATTCCAAACCCT
TGCACTCATAACAGAATAACACTACCACTGTCAAAGCACTGCAAAAACCTTTAGGTCTTGAGTTGCACTATAAATATGTA
GATTCTTCATATTTTTTTCACTCTACACATCTTACATCTGTGCAATTAAGCACACGCCCAGCAACAAGCAACACGCAACA
CGCAACACGCAACACGCAACACGCAACACGCAACACGCAACACGCAACACGCAACACGCAACACGCAACACGCAACACGC
AACACACAACACGCAACACGCAACACACAACACGCAACACGCAACACACAACACGCAACACGCAACACACAACACACAAC
ACACAACACACAACACGCAACACGCAACACGCACCACGCAACACACAACACACAACACACAACCCGCAACCCACAACCCA
CAACCCACAACCCACAACCCACAACCCACAACCCACAACCCACAACCCACAACCCACAACCCACAACCCACAACCCACAA
CCCACAACCCACAACCCACAACCCACAACCCACAACCCACAACCCACAACCCACAACCCACAACACACAACCCACAACCC
ACAACCCACAACCCACAACCCACAACCCACAACACACAACCCACAACCCACAACCCACAACACACAACACACAACACACA
ACCCACAACACACACAACCCACAACCCACAACACACACAACCCACAACACACACAACCCACAACACACACAACCCACAAC
ACACAACACACAACACACAACCCACAACCCACAACACACACAACCCACAACCCACAACACACACAACCCACAACCCACAA
CACACACAACCCACAACACACACAACCCACAACATGCAACACACACAACACACAACATGCACAACATGCAACACACACAA
CACACAACATGCACAACATGCAACACACACAACACACAACATGCACAACATGCAACACACACAACACACAACATGCAACA
TGCAACATGCAACATGCAACATGCAACATGCAACATGCAACATGCAACATGCAACATGCAACATGCAACATGCAACATGC
AACATGCAACATGCAACATGCAACATGCAACATGCAACATGCAACATGCAATCTAACTACACAGAAAAACAGATATGGTT
CTGGCTTCCCACACAGTTTACCACCACTATTCTTTAATACTTTCCAAACAAACTTTTGTTTCAGCTTTGACACAAATTAG
TATTACACAC

>p.polycephalum_GT_AG_Contig884.5_6099,7013_1
PWM=homo_gtag_U12;5'ss_score=1.5721;bsite_score=1.6373
TTTTCCCGCAGTATCCCTTCCCCCCCCCCCCCCTTTTGAGTAAACCCAAAAATTCAACAAGTTCATGTTTTTTGTAGAT
AAAATTTTAAGCAAAATCATATGGTTTTTCACCCATACAGGTGATTTCTCCACACATGTGTGCAGGGGAAAAAACCATTA
CTCCAAAAATTGCCCTTGGGGGATTTATGAGCCCCCAAAGGGCATATTTTTAGAATGTTGTGGGTGGCAGCTCTTGTTGC
TTCACAATGAGAGCTATTTTCATGATGACACATATAAAAAATTGCACAATGATCATGATGAAATCATGACACAATTGCAA
CCAATTGGAACGTGTTGTGACCACAATAATTATTTGTATGGTTGTGATGCAATTGTGATGTGATTGTGACACAATTGTGA
CATTATGATTACACAACATCGTTATTGTGCACAGCATCATGATTTTTTCACCAATATAAATGCTATTGTGGAACTTTTTT
TGTGTTTGTCAATTTTCAACAGGAGTAGGAGTGTTGGAACACCCACATTTTTCTCCATTTTAATCTATAAAGCTTTTACA
TAGAGACATCAAAAAATTCTGTTTTTTCCTGATGTGGAAACAAAAATTCAATTTTTTTAGTGCTTTTTTTTTAATTTTTT
GCCATTAAAAAAAGATAAAAAATAGGCCCAAATGTAGGGAAGTTGTCATGACGCAAAAATCGTTGTGAAAAAGTCAAAAA
ACCAGTTTTTTAGGCCTAACCGGTGATTAGACCTAAACATGTAATCTAAAAGTGATGTATTTTAGATAAATAATATTCAC
AGCTAAAAATATTTTTATATTTTTATCTGTGACAGGCTTTCCTAAAAAGGAAATTTCAAACAATTTTGCTTAATAAAATT
TATCTTGAAGTCTTGACCACATTAGGTATTATCAA

>p.polycephalum_AT_AG_Contig5217.2_1386,1551_2
PWM=homo_atac_U12;5'ss_score=2.8864;bsite_score=0.9176
AGTGTTGACAATATCTTTACATATTTGGCCCTAGGCTCTTCTAGGCTCTTCTAGGCTATTCTAGGCTTCTAGACTCTTCT
AGACTCTTCTAGGCTCTTCTAGGCTCTTCTAGGATCTTCTAGTATAGGTTCAGGTACCTGAATTTGACCACCTTAGATAC
TAAAAA

>p.polycephalum_GT_AG_Contig3776.2_775,866_2
PWM=mus_gtag_U12;5'ss_score=1.5178;bsite_score=3.2791
ACGGAAAAGTGTATCTTTTATAACTATTTTAACGTGCTAGTTATCTACCTTAACCTGCTCCCCTTATTTTTAGTGAAGAA
AGGTATATTACT

>p.polycephalum_AT_AC_Contig6639.1_794,908_2
PWM=homo_atac_U12;5'ss_score=3.2312;bsite_score=1.5562
AGAATGGGAGATATCCTTTTTGTTAATGCTTTCTACAAGCTCTTTCATAGTATTTCCTCTAATATTTCTCAGCTCTCTTT
CGTCTCTAACTTGTGACCAAACTACAGATTACCTA

>p.polycephalum_GT_AG_Contig810.8_4518,4653_1
PWM=droso_gtag_U12;5'ss_score=2.0611;bsite_score=0.5977
GCCCATTTTCGTATCCTTTGCATAATTTGATGAAATTGTGCAATACATCGCCACACCACACATCACGCACCACACACATG
TCATGTCATACACCAAGTTGCAAATGCTTTTTGACATACTCTTTAGTTGACGAGCC

>p.polycephalum_GT_AG_Contig7429.1_1966,2050_2
PWM=mus_gtag_U12;5'ss_score=1.8120;bsite_score=0.7635
AGGGTCCTCAGTATCCTCTTTCCTCCTTTCCCTCCCTCTTTTGTATCACCTTATTAGCCACTTTGATACTAACAGATATG
ACTTG

>p.polycephalum_AT_AA_Contig8299.5_1368,1540_2
PWM=homo_atac_U12;5'ss_score=3.6345;bsite_score=1.4769
TTTGGGTGGCATATCCTTTTTTATTATTATTATTATTATTATTATTTTTTGTTTGTTTGTTTGTTTGTTTGTTTTTTTTT
TTTTTGTACTTTTGTTTGCATTTGTTTTTCATTTTGTTACCTCTTTTTCTTGTTCCACCTTTATCATCTGTGATAATGTA
AAATTATTTGGCG

>p.polycephalum_AT_AC_Contig2642.5_4037,4111_1
PWM=homo_atac_U12;5'ss_score=2.9049;bsite_score=1.9539
CCAACAACAGATATCCTTAACCTTTTAACACTCGTTACCTTTGAATTCACTTCTACTCCTCATACAAATATGTGC

>p.polycephalum_AT_AG_Contig6639.1_792,908_2
PWM=homo_atac_U12;5'ss_score=3.2312;bsite_score=1.5562
AGAATGGGAGATATCCTTTTTGTTAATGCTTTCTACAAGCTCTTTCATAGTATTTCCTCTAATATTTCTCAGCTCTCTTT
CGTCTCTAACTTGTGACCAAACTACAGATTACCTACT

>p.polycephalum_AT_AC_Contig205.3_835,933_2
PWM=homo_atac_U12;5'ss_score=3.1464;bsite_score=0.7377
AGACATGTACATATCCTCCAGATTATATGTTCATTTTCTCTTTTTGTTGGAATTTCTGAACTTGGGTTCTTTTCTTTAAT
TTTAAATACAAATGTTTGG

>p.polycephalum_GT_AG_Contig3942.7_1496,1592_2
PWM=homo_gtag_U12;5'ss_score=1.5531;bsite_score=0.6374
GAGTACATTTGTATCATTTGCATATTTCTTGACAACCTCCTTCACAATAATAAGAAAATACGTTGCAATTTGTCGTGACA
AAAGAAGCATTACATCT

>p.polycephalum_GT_AG_Contig4762.2_192,273_1
PWM=homo_gtag_U12;5'ss_score=1.5685;bsite_score=0.7627
TATTGTGGATGTATCCTCCTTTTCTTCTTCTTTTTCTTTCATTTGCTCTTTCTTGCTTCTTTCATCCTTAAGATATGCAA
GC

>p.polycephalum_GT_AG_Contig7958.1_193,528_2
PWM=mus_gtag_U12;5'ss_score=1.6722;bsite_score=0.8112
GGTGCTTTTTGTATCCTCGAACAGCGCCACAATGTCACGCATCCACACGCAACACGTAACGCAGGGCACGCAAACTCATG
CGCAAAACTTGAATCCGGAAGTGCTCCATCACCACGAGTAGTTCGTAAATTCAGCACATCTGTTCTCACTAGAGAGGCCA
AGTAACACTGAACCAGGAGGCACATCAAGCACCCCAACACCCCCAGCACCCCCAACGCACCCCACGCCTAGCACCCACTC
ACAGCGCTCCCACAGCGCATCACCAACCATTACCAAGTGCTAGAACCCATATTACAATACTATGCAACTTTGATCTGATT
CACTAGATCTGGGATT

>p.polycephalum_AT_AG_Contig86.3_6039,6142_1
PWM=homo_atac_U12;5'ss_score=3.0262;bsite_score=1.1763
GACAGATAGCATATCTTTCTCCGCCTACTCATTCATTTTGCTTCTGGTCTTCTCTTGGTCATAGCTGTTAATTTCCAGCT
TTTATGCTTTGTAGATATTCACTT

>p.polycephalum_AT_AG_Contig11.8_1939,2058_2
PWM=homo_atac_U12;5'ss_score=3.5287;bsite_score=1.8841
CCCAAAAAACATATCCTTTTCCTTCTCCCTCAATGCTTTATTTCTAGTACTCCTTCCTTATTTGTCTCCCAATTCTTACC
CACCCCTTCACAAGATTTGACATGTACTAGATATACAAGA

>p.polycephalum_GT_AG_Contig2879.3_1778,1878_1
PWM=homo_gtag_U12;5'ss_score=1.6676;bsite_score=0.5693
ATTTTGCATCGTATCCTATCTAAAAGGATTAATTACGCCTCCTATATCCTTCTCGTTTTCATCCCTACGACCATGTAATG
ATTACACATAGATATGGAGGG

>p.polycephalum_GT_AG_Contig2286.11_987,1282_1
PWM=droso_gtag_U12;5'ss_score=2.1493;bsite_score=0.7111
CAGCGACTTTGTATCCTTTTTAGTGCACTTCCATTCACCACACCACGCACGCGCACAATCACGTCCCGCATGCGCACGAC
ACGCGCACGCATGCACACGATCACAAACTAACACTCAGGTAAGAAGTACACGAAAAGCCCCGCACGCAAGAATTTTAAGC
AGTTACAACACACTTGTGCGCGCAAGAATTTTAAGCAGTTACAACACACTTATGCGCGCAAGACGCATTTGTACACCTAT
TCACGCACTCGCATTCACTCTTAAAATGCCAAATTGACTTGAAAAGATTCCAAAAA

>a.castellanii_GC_AG_gnl|ti|858054168_443,553_2
PWM=droso_gtag_U12;5'ss_score=5.3518;bsite_score=2.9555
CTTGGCGGCAGCATCCTTTTACCCCCCCCCCCTACACAACTCAGAACCCATCCTCTTTGGCACAGGCGTTTCTTCTTTCG
TTGCTTCCTTAACGTGGGGAGACATGGGCTT

>a.castellanii_AT_AC_gnl|ti|858048643_376,512_2
PWM=homo_atac_U12;5'ss_score=6.0991;bsite_score=3.0255
CGCACTGCTCATATCCTCTTCCCCCCCCCCCCCAAATCCTCTTTCTCTGCTTCTTTTTATCATTACCACTTGTGCCAACC
ACCACGGTGCGTGGTCGTGTTGTTGTTATTCCTTAACCTGGCCGTACTTCTTCAAGG

>a.castellanii_GT_AG_gnl|ti|858058852_231,408_2
PWM=droso_gtag_U12;5'ss_score=3.1774;bsite_score=0.6749
CACGCTCAAGGTATTCTTTTGTTCCCCCCCCCCAATCCGACACCACTCCCGGCTTCGTTCTCCGGCCGTTTCCGTCATTC
TCCCATTCTTTTCTTTCCCTTCCGGTTTCCTCGACCGGACCTGTGGGCCGACAGGACCACTCACCCGTTATTCTCTTCGG
TGTGACAGGTGAGGCTGC

>t.pseudonana_AT_AG_Thaps3_chr_11a_700847,700969_2
PWM=homo_atac_U12;5'ss_score=5.2124;bsite_score=0.4309
TCGAGTAGAAATACCTTGAGCGCAAGAATGAGCAGAGTGTATTGACGGGGAATCATGAAGGGTGAGTGATGCGGTAGATG
AGAAGGCGAAGAACCGACATAAACATGTTCAAGATACCTTGAG

>p.blakesleeanus_GT_AG_Phybl1_scaffold_1_1854204,1854361_1
PWM=homo_gtag_U12;5'ss_score=4.8439;bsite_score=2.0552
GGTCTTCTTTGTATCCTTTATATATAAATATGGCTGCTTTAAACTGAGTTTCGAGCGAACCTGAAAAGATGAATAACCAT
GAATATAACAGATGATTACAATGTGATACTTTTATTATGCCTTTACTCTTTTAATGTCTTGATTTTAGGTTATTATGT

>p.blakesleeanus_AT_AC_Phybl1_scaffold_1_2350613,2350777_1
PWM=mus_atac_U12;5'ss_score=7.3333;bsite_score=3.5282
TAACTAGCGAATATCCTTTATTGATAAAAATTAGATACATAAATATGTGCATAGATATATACATATATATATATACATGT
ATATATATGTGTGTATGTTTTATATCTAGATTTTAAAGATATTTTATTTTGTCTTATCCTTAACCTTTTTCATACAGCAA
GTGAC

>p.blakesleeanus_GT_AG_Phybl1_scaffold_1_2802457,2802575_2
PWM=droso_gtag_U12;5'ss_score=4.9593;bsite_score=2.7402
TGGAGACTGAGTATCCTTTTTCATAATGATATTTAGATGCCCCGGATGTACACTATACAAAGCCAATTCTCTTCCTATCC
TTCACTCTTACCCTTTTTTCTCCCGTTAGCTATGGAAGA

>p.blakesleeanus_AT_AC_Phybl1_scaffold_2_1420276,1420429_1
PWM=homo_atac_U12;5'ss_score=7.1568;bsite_score=2.5196
GAGCTGGCAAATATCCTTTTTATAAAAGAAAAAGAGAAAGAAAGAAATTATCATTATCATTAATAATGTCTGTTGTGATT
GTAATTGTGGATATTACTCTTTATATATATTTACTATTGTGTATTTCCTTTACCATCTATTTACATCATGATAT

>p.blakesleeanus_AT_AC_Phybl1_scaffold_25_553821,553914_1
PWM=homo_atac_U12;5'ss_score=7.1568;bsite_score=1.6576
GCATTTTCAAATATCCTTTTTTCTTATACATACATAGCACATAAATTGAACGAACAATAGCTGTTAGCCTTGACTTCTTT
GTACAGTATTGGGA

>p.blakesleeanus_GT_AG_Phybl1_scaffold_10_664842,665014_2
PWM=droso_gtag_U12;5'ss_score=5.2961;bsite_score=2.0025
GAAAGGAGTCGTATCCTTTTTATTATATTTACACACCAATACCAATACTAACACTGTTATAATAGCAATATCAATATACA
GCTTGACATTATTATTATTATTATTATTATTTATCCTTCACTTTGAATTATTGTATCTTCATTCATTCTTTACCAAATAA
TAGCTCTCGAGGA

>p.blakesleeanus_GT_AG_Phybl1_scaffold_1_1467245,1467535_2
PWM=droso_gtag_U12;5'ss_score=5.1989;bsite_score=3.1388
AAGACTATGCGTATCCTTTTTATATACTTGTATGCTTGATATTACATATACAGCATCATCTTTTTAGCCAAAAAACACCC
TGGTCTAGATATGAACCAGAAAAAAAGAAGGATATTTCAATAAGTAAATCTGACATATCACGCCTACGTACATAGGAAAA
GACGCCAAATTACAAATACGATCTTTTATTCATGACGCGTGTGACATGATTCGCACCATGTTCGGCATTCAAGCCTTCTC
TTTATCCTTGACTTCCTTCTACTTTGCTATCTTTATTTTAGTACATATCTT

>p.blakesleeanus_AT_AC_Phybl1_scaffold_2_2756437,2756544_2
PWM=droso_atac_U12;5'ss_score=7.6558;bsite_score=3.6049
ATTGTTGATCATATCCTTTTTTTTTATTGCTGTAATAATTTGAGAGGTGTAATTTTCACAAAGCTCAATACACTATGTCC
TTAACAGTGTTTGGTTACATATGCATTT

>p.blakesleeanus_GT_AG_Phybl1_scaffold_28_246055,246172_1
PWM=droso_gtag_U12;5'ss_score=4.8995;bsite_score=2.8894
ATGGAAGTCAGTATCCTTTTGAATAATAGATATAATCGAAAAGTTGTGTGTACAGGTTCACACACAAATAATATCCTTGA
CTTCACTTTTGTTTTCCCCTCATTACAGACACAGCGCG

>p.blakesleeanus_GT_AG_Phybl1_scaffold_49_94326,94470_2
PWM=droso_gtag_U12;5'ss_score=5.2047;bsite_score=3.2133
CTACGAATTCGTATCCTTTTTTTAAGTGTGTGTATTGTTTGTATAATGGATAGATATACGAATATGTGGAAATGCCAACT
ACAATTGAGTGCCCACATCCTTGACTCTTGTATTTCATGTCGATTCGTATTTTAGACACAGGGAT

>p.blakesleeanus_GT_AG_Phybl1_scaffold_2_2386232,2386450_1
PWM=droso_gtag_U12;5'ss_score=4.9068;bsite_score=4.0801
CCTGGAAATAGTATCCTTTTTTACTTAACAGCTTGAGATCTGAGGGAAAGTGGTTTAGAATCACTTTGGCTTATCTACAG
ATGATGCCATTGGTTTTTGTGACCACTAAAACAACGATTTTTCTATATTGGACATTATGACAGAGCAAAATGCTTTCTGC
ATCGCTCGGTAACTGTCTGCCTTAACCTCTTTCTTTTTTATTTTTATAGTGAGTCTTCC

>p.blakesleeanus_AT_AC_Phybl1_scaffold_37_273516,273662_2
PWM=homo_atac_U12;5'ss_score=7.2035;bsite_score=2.3425

GATTCTTAAAATATCCTTTTTATTATTATTGCATTACTACAAAAACTAGTTTAACAGAGAAAACAAATCATTTACTCTCT
CGAGAAAGAGTTTCTCTCTCTCTCTTATTTTCACAAACATTCTTAACCACTCTGTACATATTCACCC

>p.blakesleeanus_AT_AG_Phybl1_scaffold_9_53574,53890_2
PWM=homo_atac_U12;5'ss_score=4.7987;bsite_score=2.9932
CGCGACGAAGATTTCATGAAGATCTGGGAAGATGTGTTGGAAAAGAACGGATTTTGCATGCGCCACAAGCTGGAGCGCAA
CAGTCTGACGGATGCGACCATCGCGCCCTTGAGTCCGTCTTCATTTGTTGGTGAAACACCTGTGCACATCAAAGTTATCG
GAAGACACATGAACGGCTCCTTCCGATGCCACATCGGGACCGGCAAGGCATCCCGCCCCATTCGGTCCACCGAACTGTGT
CAGCAGATGCGACTCCTTGCAGTGTTCTTCAAAGCCGAGGGCAGCCGAAGCCAACTGATCTTGCCAGATTTCATTCT

>p.blakesleeanus_AT_AC_Phybl1_scaffold_3_1653934,1654069_1
PWM=homo_atac_U12;5'ss_score=6.8277;bsite_score=1.7296
GCCTATGCAGATATCCTTTTATATATATATACATATGTTATGGTCGCTTGTCTTTTGTTCTTTAACATACATGAAAAAAT
AACTAAAAAAGAACAGACCTTTATTACATCCTTGACCTAAACTTACATTCCTCAGG

>p.blakesleeanus_AT_AC_Phybl1_scaffold_1_648131,648213_1
PWM=mus_atac_U12;5'ss_score=3.9619;bsite_score=1.0454
ACACACACGCATATATATATATATATATATAGACGTTAATATATTATTATATTCTTATCATTATCATTATTACCTTTGGA
GCT

>p.blakesleeanus_GT_AG_Phybl1_scaffold_5_1782789,1782946_1
PWM=droso_gtag_U12;5'ss_score=4.8679;bsite_score=2.6529
ACTGCTGGAAGTATCCTTTTATATGCACATATGGCATTAAATCTTCTTTTGACTATCATTTGTGTATATTGGTGTATAGA
TATATATGCATATATATATACACGTATACACGTATACTCCTTTACTCTTTTAATGTGTCTATTTCTAGATGTCAAATT

>p.blakesleeanus_AT_AA_Phybl1_scaffold_22_166814,166906_2
PWM=mus_atac_U12;5'ss_score=5.6984;bsite_score=1.3361
GGCTCTTTGAATATCTATCAAGGAAATTCAAGTCTGATTAGAAGAATGTAAACAAAGCTATTAAAGCGACCAAGCCGATA
TAAATACCTTTTT

>t.spiralis_GT_AG_Contig12_1086031,1086157_2
PWM=droso_gtag_U12;5'ss_score=3.3622;bsite_score=2.0318
GTTGAAAATGGTATTCTTTTTCTTTTTCTTTTTTCTTTTTGCTTTTGACGACATCCTTGATTGTGATTGTTATATTTAAA
TTTCCATTACAAAATTACAACCGCGTTGTTCTTTCAGTGCCGGATTA

>t.spiralis_AT_AC_Contig9_356821,357022_2
PWM=homo_atac_U12;5'ss_score=6.4289;bsite_score=4.8240
GCAGCGTATCATATCCTTTCAATATTTCTTAATTGGTTGTTTTATTTTTATTTAATTTGTAGAGTAATTGGTTGTACAAT
TTAAAATTAATTTTGTTAAGTTAATAATTTAATGCATTTTTAATATTTTAATTATATTGATATATTTAATGAACGTATTG
TTTGTATTTTCCTTAACTTCATATGTTTTTACGTACTTCGTG

>t.spiralis_AT_AC_Contig2_3210581,3210694_2
PWM=homo_atac_U12;5'ss_score=6.2125;bsite_score=4.4284
TTTGGAAATTATATCCTTTCATTGGAATAATTTTTGTTCTATTATAATTAAGTTTATTATTTGTGAAATTTCATTTCCTT
AACGTTAGATTTTTGTTGTTTTACTGATGTTCCT

>t.spiralis_AT_AC_Contig0_12048829,12050943_1
PWM=mus_atac_U12;5'ss_score=6.2488;bsite_score=4.4284
CTTACATAGAATATCCTTTCAATTATTACATTTTATTGCAGGAGGTTGAACTGATAAAGCATTTATGCTTAGCAATGTAA
ATTGATTATTTTCTTCAAATTTTTTGCATTTTTATGTTCAGATGAAATTTTTTTTTGTTTTCATTACTTCACATTTCATT
CAGATTTTACTTACTTATTATTCCTCAACACTAATAGGATAGAGCGTTTTTATCACAATTTCGATTGTTTAGATAGTCTG
CGCTAGTAATTTCAAATCACTAGGCACTCGCGTAGTTTATGGTCAGTTATTTTTTCGTGCTTTTCATCCTAGGGGCTTTG
CGTGAATCTTCGCATTGCTACTATTGGCTGAACTAATTTTTTGGTTCTAGCAAAAGGGCGTTTAAAAGACGTAAAGCGAG
AATGAAAGAATTTCTTTCGTCAATTTGTTGGTGTGTAAAGTAAAAGATTCGCTCGCTCGCGACTAGTGACAAAAAAGTCA
AGATGCAATAAAGTCAAAGACCAAGACTAAAGCATACTTTAAACCAGAAGACAGAGACAAGAAAGATAACTATATTTAAA
AGCTTTTGGCAATCAGGTAGCAGCCCAAAAATTTCTGCTGTTTACTTTCCACAACAAAAGGGGGAAGTGATAATAATTAG
CTTCTTACTGTAATTAGGATAGGTGAATTATATTGATAACATAATAGTGTACAATGTAAATAGTTTACATAAGCCTCGTT

TGGGTATATCGTGCGTTTGGTCGGTTAATTATTTGCTGCAACATTCACAGCAAGCTTGTCTTGTGTAATTTAACTCTATG
TGATGCATATTCAATCGAAATGTTCATTTTAAATGAAACATCGCAAAATTATGTTTAAAACGAGCAGAAATTAAATGACT
ATTTTATAAATGCATATTCAAATGAAAATTATGAATGGCATTCAATTTTATTAATTTGTATTTGTAATCGAATCACCATA
GAATGTAAATGTTATACAGAAATTTAAATTGTAGATTGTTTGAGAAACTTTTAGAAATATATTTCTTGATTGATATTTTC
TGATGAATCTGCTTTTGTAATTGTTATTCGGGTTCCTTTTTGTTTGTGGGTTTTTATTGGTATCAAACATGAAAGCATTG
CAAGATGGTTTATCCGTGATGTAAACAATAAATAGTACTGTACTGTACTTGTGTTTTTCGATTGAATTGCAAGCGGCTGT
TCTTTGAATTGCGTGGGTTTTGATGTTTGTTGTTTTGGCGATATAAAATTTTCTTTTGAAACTTTTCGTTGACAATGTAA
AAATCGAAGAAATCATTAGGATGACACTAATGATAATTTAATCACCTTTTTATCATTTGTTTGACAGCTGGTGGTGTTTA
TTTAATTTCGATAATTTTACGAAGAATCCTGCTACCTGATGCAAATGTTCATTTTACATCATTTTTATTTAGGCCTTTAT
CGTTGTTAATAATCATTTTAATCGTAAAATTGCAGAGTAGAACTTATGTAATATGCGGTGACGTCTAGCGGTTGTTTTAA
GAGGTACATAGAGATTGAATTGTGGGATTGAAATGCGGTGAATGGGCGCATATGGAAAAAAGTTTTATGAAATATACCTT
CATTATATAAAAATTTTAATAAAACAAGTATTATTATGAACGATTTCAACGAATAATTTTCTTTCTTTCCACAAACAAAT
TGTAATTGGTCTGATTTATTTTTTAGTTGGTTGTTTTATGTGGTTTTGTAGATTATTAATTTGTTTTAATGTTAAATTCG
TAGAGATTGTAATTTTTTATATTTCAAAGTGTTTTACATAAGCATAGCACATATAATAATTGCCGCTGGAAACATAGAAT
TCTTTATCCGTAATGTATACATATGTTATTGCATTTCATTGATCTATTTATGTGATGGGTACAATGTGGAAGTTATTTTT
TTTTACGAAAATACCTGCATTTCAAGCATTATGGTAATGTTTATTACGTAAATTTGAAAAAAGAATCGTGCAGTGGGTAA
AACCTGAATGAAATGTGTAGTATGAAATCTTTTTTTAATTATTACCTAGCAGTTATCGAGTTAGAAATTGGGCATTGTTA
TATTTCCTTAACGGGTATGGTTTACGTTCGAAGAG

## Multiple sequence alignment of three genes in *R. oryzae* encoding s13

```
gi|203801194_19724-20173      GGTCGTATGCACACTCCTGGTAAGGGTATCTCTAGCAGTGCCCTCCCTTA
140710-141126                 GGTCGTATGCACACTCCTGGTAAGGGTATCTCTAGCAGTGCCCTCCCTTA
gi|203801322_89048-89464      GGTCGTATGCACACTCCTGGTAAGGGTATCTCTAGCAGTGCCCTCCCTTA
                              **************************************************

gi|203801194_19724-20173      TCGCCGCACTCCTCCTTCTTGGGTCAAGACCACTTCTGAAGAAGTTGTTG
140710-141126                 TCGCCGCACTCCTCCTTCTTGGGTCAAGACTACTTCTGAAGAAGTTGTTG
gi|203801322_89048-89464      TCGCCGCACTCCTCCTTCTTGGGTCAAGACCACCTCTGAAGAAGTTGTTG
                              ****************************** ** ****************

gi|203801194_19724-20173      ACATGATCTGCAAGAATGCCAAGAAGGGTCTTACTCCCTCTCAAATCGGT
140710-141126                 ACATGATCTGCAAGAATGCCAAGAAGGGTCTTACTCCCTCTCAAATTGGT
gi|203801322_89048-89464      ACATGATCTGTAAGAACGCCAAGAAGGGTCTTACTCCCTCTCAAATTGGT
                              ********** ***** ***************************** ***

gi|203801194_19724-20173      GTCATCCTTCGTGACTCTTATGGTATTCCTCAAGTCAGATCCATCACTGG
140710-141126                 GTCATCCTTCGTGACTCTTATGGTATTCCTCAGGTTAGATCCATCACTGG
gi|203801322_89048-89464      GTCATCCTTCGTGACTCTTACGGTATTCCTCAAGTCAGATCCATCACTGG
                              ******************** *********** ** **************

gi|203801194_19724-20173      TAACAAGGTCCTCCGTATCTTGAAGTCCAGCGGTCTCGCTCCTGAAGTTC
140710-141126                 TAACAAGGTCCTCCGTATCTTGAAGTCCAGCGGTCTCGCTCCCGAAGTTC
gi|203801322_89048-89464      TAACAAGGTTCTCCGTATTTTGAAATCTAGTGGTCTCGCTCCTGAAGTCC
                              ********* ******** ***** ** ** *********** ***** *

gi|203801194_19724-20173      CCGAAGACTTGTACCATCTCATCAAGAAGGCTGTCTCCATCCGTAAGCAC
140710-141126                 CCGAAGACTTGTATCATCTTATCAAGAAGGCTGTCTCCATCCGTAAGCAC
gi|203801322_89048-89464      CCGAAGATTTGTACCATCTCATCAAGAGGGCCGTCTCTATCCGTAAGCAC
                              ******* ***** ***** ******* *** ***** ************

gi|203801194_19724-20173      TTGGAACGTAACAGAAAGGACAAGGACTCCAAGTACCGTTTGATTCTTAT
140710-141126                 TTGGAACGTAACAGAAAGGACAAGGACTCCAAGTATCGTTTGATTCTTAT
gi|203801322_89048-89464      TTGGAACGTAACAGAAAGGACAAAGATTCAAAGTACCGTTTGATTCTTAT
                              *********************** ** ** ***** **************

gi|203801194_19724-20173      CGAATCTCGTATTCACCGTCTTGCTCGTTACTACAAGACCTCTGGTCAAC
140710-141126                 TGAGTCTCGTATTCACCGTCTTGCTCGTTACTACAAGACCTCTGGCCAAT
gi|203801322_89048-89464      TGAATCTCGTATTCACCGTCTTGCTCGTTACTACAAGACCTCTGGTCAAT
                               ** ***************************************** ***

gi|203801194_19724-20173      TTCCTCCTACCTGGAAGTATGAATCTGCCACTGCTTCTGCTATGGTTGCT
140710-141126                 TGCCTCCCACCTGGAAA---------------------------------
gi|203801322_89048-89464      TGCCTCCCACCTGGAAA---------------------------------
                              * ***** ********
```

## EST sequences of two of the S13 genes in *R. oryzae* with the same intron position

EE001485.1 upper
EE010126.1 lower

```
  1 CTCAAGTCAGATCCATCACTGGTAACAAGGTTCTCCGTATTTTGAAATCTAGTGGTCTCG 60
  1 CTCAGGTTAGATCCATCACTGGTAACAAGGTCCTCCGTATCTTGAAGTCCAGCGGTCTCG 60
      Q  V  R  S  I  T  G  N  K  V  L  R  I  L  K  S  S  G  L  A
      Q  V  R  S  I  T  G  N  K  V  L  R  I  L  K  S  S  G  L  A

 61 CTCCTGAAGTCCCCGAAGATTTGTACCATCTCATCAAGAGGGCCGTCTCTATCCGTAAGC 120
 61 CTCCCGAAGTTCCCGAAGACTTGTATCATCTTATCAAGAAGGCTGTCTCCATCCGTAAGC 120
      P  E  V  P  E  D  L  Y  H  L  I  K  R  A  V  S  I  R  K  H
      P  E  V  P  E  D  L  Y  H  L  I  K  K  A  V  S  I  R  K  H

121 ACTTGGAACGTAACAGAAAGGACAAAGATTCAAAGTACCGTTTGATTCTTATTGAATCTC 180
121 ACTTGGAACGTAACAGAAAGGACAAGGACTCCAAGTATCGTTTGATTCTTATTGAGTCTC 180
      L  E  R  N  R  K  D  K  D  S  K  Y  R  L  I  L  I  E  S  R
      L  E  R  N  R  K  D  K  D  S  K  Y  R  L  I  L  I  E  S  R


181 GTATTCACCGTCTTGCTCGTTACTACAAGACCTCTGGTCAATTGCCTCCCACCTGGAAAT 240
181 GTATTCACCGTCTTGCTCGTTACTACAAGACCTCTGGCCAATTGCCTCCCACCTGGAAAT 240
      I  H  R  L  A  R  Y  Y  K  T  S  G  Q  L  P  P  T  W  K  Y
      I  H  R  L  A  R  Y  Y  K  T  S  G  Q  L  P  P  T  W  K  Y

     ' =splice site A/T
241 ATGAGTCTGCTACTGCCTCTGCTATGGTCGCTTAAACAAGCTGTTTGTCGCATCGATTTT 300
241 ATGAGTCTGCTACTGCTTCTGCTATGGTTGCTTAAACAAGAAGGTTATTGCATCGATTTA 300
      E  S  A  T  A  S  A  M  V  A  *
      E  S  A  T  A  S  A  M  V  A  *
```

## Alignment of the introns of the S13 genes above, including 10 nt of flanking exons.

```
supercontig_3.5_3082913_308308      CCTGGAAATA GTATCCTTTTTTGATTATACACTAACATTTCTTAGAGAGA
supercontig_3.10_1679086_16792      CCTGGAAATA GTATCCTTTTTTGATTATGTACTAACGCTT--TAGAAAGA
                                    ********** ******************  ******  **  **** ***

supercontig_3.5_3082913_308308      GAGCGTGTATCCATATTT--ATTGCCTATATTGGACATTACTTGGAGTTG
supercontig_3.10_1679086_16792      GAACGTGTATTCATTTTTTTATTGCCTATATTGGACATTACTTGGAGTTG
                                    ** ******* *** ***  ******************************

supercontig_3.5_3082913_308308      CTTTTTCGAAGAAAAACTGCTCGGGTAACTGCCCTCCTTAACCCAATTCT
supercontig_3.10_1679086_16792      TTTT-----AGAAAAACTGCTCGGGTAACTGCCCTCCTTAACCCAATTCT
                                     ***     *****************************************

supercontig_3.5_3082913_308308      TTGTTTTTTTATAGTG AGTCTGCT
supercontig_3.10_1679086_16792      TTGTTTTTTTATAGTG AGTCTGCT
                                    **************** ********
```
